# Efficient Reverse-Engineering of a Developmental Gene Regulatory Network

**DOI:** 10.1371/journal.pcbi.1002589

**Published:** 2012-07-12

**Authors:** Anton Crombach, Karl R. Wotton, Damjan Cicin-Sain, Maksat Ashyraliyev, Johannes Jaeger

**Affiliations:** 1 EMBL/CRG Research Unit in Systems Biology, Centre for Genomic Regulation (CRG) and Universitat Pompeu Fabra (UPF), Barcelona, Spain; 2 Department of Mathematics and Computer Sciences, Bahçeşehir University, Istanbul, Turkey; University of Tokyo, Japan

## Abstract

Understanding the complex regulatory networks underlying development and evolution of multi-cellular organisms is a major problem in biology. Computational models can be used as tools to extract the regulatory structure and dynamics of such networks from gene expression data. This approach is called reverse engineering. It has been successfully applied to many gene networks in various biological systems. However, to reconstitute the structure and non-linear dynamics of a developmental gene network in its spatial context remains a considerable challenge. Here, we address this challenge using a case study: the gap gene network involved in segment determination during early development of *Drosophila melanogaster*. A major problem for reverse-engineering pattern-forming networks is the significant amount of time and effort required to acquire and quantify spatial gene expression data. We have developed a simplified data processing pipeline that considerably increases the throughput of the method, but results in data of reduced accuracy compared to those previously used for gap gene network inference. We demonstrate that we can infer the correct network structure using our reduced data set, and investigate minimal data requirements for successful reverse engineering. Our results show that timing and position of expression domain boundaries are the crucial features for determining regulatory network structure from data, while it is less important to precisely measure expression levels. Based on this, we define minimal data requirements for gap gene network inference. Our results demonstrate the feasibility of reverse-engineering with much reduced experimental effort. This enables more widespread use of the method in different developmental contexts and organisms. Such systematic application of data-driven models to real-world networks has enormous potential. Only the quantitative investigation of a large number of developmental gene regulatory networks will allow us to discover whether there are rules or regularities governing development and evolution of complex multi-cellular organisms.

## Author Summary

To better understand multi-cellular organisms we need a better and more systematic understanding of the complex regulatory networks that govern their development and evolution. However, this problem is far from trivial. Regulatory networks involve many factors interacting in a non-linear manner, which makes it difficult to study them without the help of computers. Here, we investigate a computational method, reverse engineering, which allows us to reconstitute real-world regulatory networks *in silico*. As a case study, we investigate the gap gene network involved in determining the position of body segments during early development of *Drosophila*. We visualise spatial gap gene expression patterns using *in situ* hybridisation and microscopy. The resulting embryo images are quantified to measure the position of expression domain boundaries. We then use computational models as tools to extract regulatory information from the data. We investigate what kind, and how much data are required for successful network inference. Our results reveal that much less effort is required for reverse-engineering networks than previously thought. This opens the possibility of investigating a large number of developmental networks using this approach, which in turn will lead to a more general understanding of the rules and principles underlying development in animals and plants.

## Introduction

Elucidating the regulatory structure and dynamics of gene networks is a major objective in biology. The inference of regulatory networks from gene expression data is known as reverse engineering [1]–[7]. It is being widely and successfully applied, from microbes to animals (see, for example, [8]–[16]). Many reverse engineering studies aim to determine regulatory structure from large-scale perturbation- or time-series data based on microarray or transcriptome-sequencing technology (reviewed in [5], [17]). This approach has two significant limitations: first, spatial information on gene expression is lost, since homogenised tissue samples or disaggregated cells are studied. And second, most resulting models are of a static and probabilistic nature, which cannot be used to investigate network dynamics (for example [18]–[20]). If dynamical models are used, they are often linear (for example [21]–[23]). Network inference using complex non-linear dynamical models is deemed a considerable technical challenge [6], [17], [24]. However, there are many important biological questions that absolutely require consideration of non-linear and spatial aspects of a system. Here we discuss such a case, and show that reverse engineering can be used for its study with a reasonable amount of experimental and computational effort.

Our research focuses on how developmental gene regulatory networks produce spatial patterns in multi-cellular organisms, and how these patterns evolve through changes in the underlying structure of the network [25], [26]. In this context, reverse engineering is implemented by fitting non-linear systems of differential equations to quantitative, spatial gene expression data (reviewed in [7]). There are many network modelling formalisms [27]–[29], and a number of powerful global non-linear optimisation methods [7], [30], [31], which are suitable for this task.

So far, only a small number of developmental systems have been reverse-engineered using dynamical models (see, for example, [32]–[34]). One of those is the (trunk) gap gene network of the vinegar fly *Drosophila melanogaster* (reviewed in [35]). This regulatory network consists of four genes—*hunchback* (*hb*), *Krüppel* (*Kr*), *giant* (*gt*) and *knirps* (*kni*)—which all encode transcription factors. Gap genes are involved in establishing the segmented body plan of the animal. They are active during a very early period of *Drosophila* development, called the blastoderm stage, which occurs before the onset of gastrulation. At this stage, the embryo consists of a multi-nucleate syncytium allowing transcription factors to diffuse through the tissue. Gap genes are expressed in broad, overlapping domains along the embryo's major, or antero-posterior (A–P) axis. They are regulated by long-range gradients of transcription factors encoded by maternal co-ordinate genes *bicoid (bcd)*, *hunchback (hb)*, and *caudal (cad)*, and are repressed by the terminal gap genes *tailless (tll)* and *huckebein (hkb)* in the pole regions of the embryo. Maternal co-ordinate and gap genes form the first two tiers of the segmentation gene hierarchy in *Drosophila*. Together they regulate pair-rule and segment-polarity genes, the latter forming a molecular pre-pattern that leads to morphological segmentation at later stages of development (see, [36], [37], for review).

**Figure 1:**
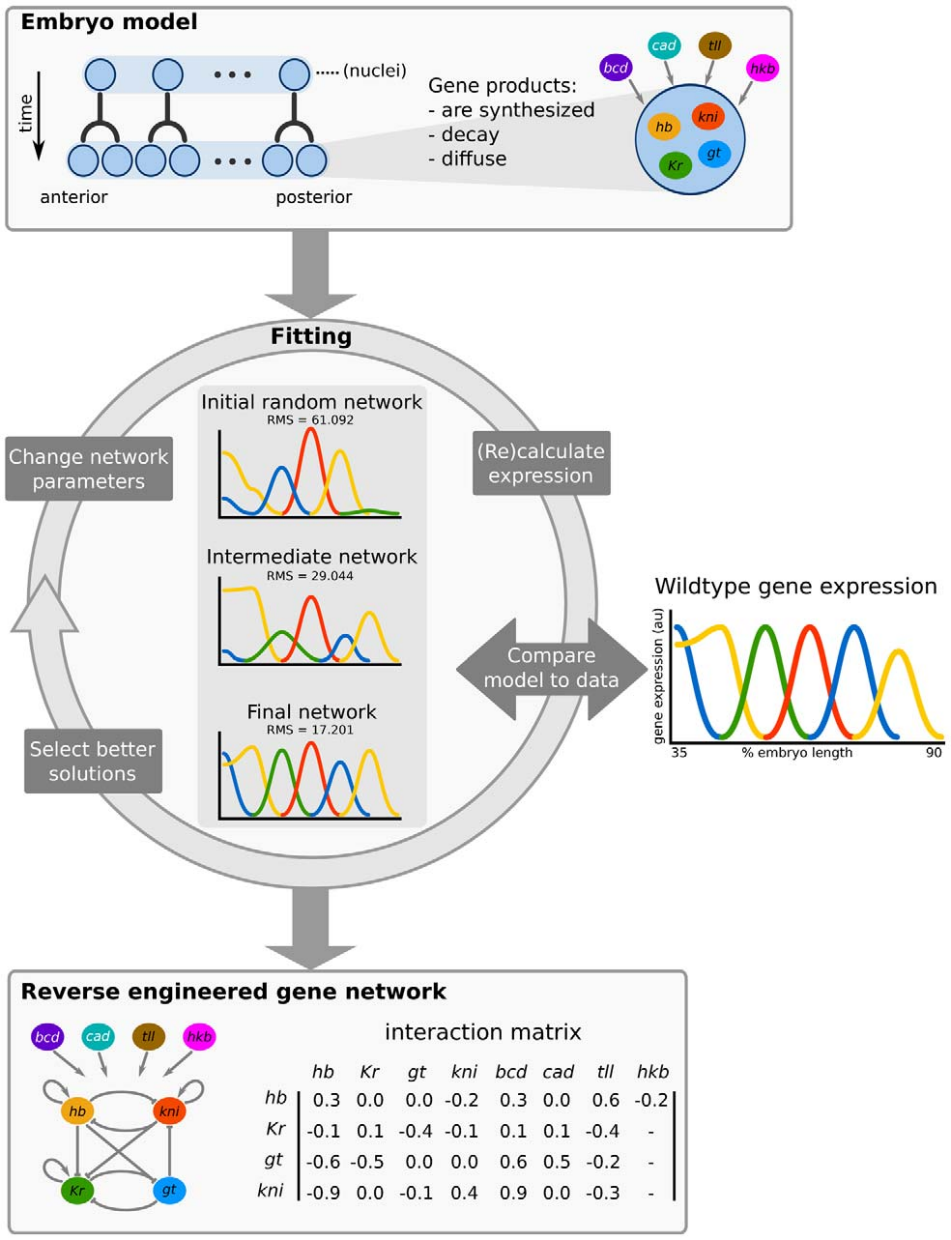
The gene circuit method. Top panel: a *Drosophila* embryo is modelled as a row of nuclei that undergo mitosis. Each nucleus contains four gap genes: *hunchback* (*hb*), *Krüppel* (*Kr*), *giant* (*gt*) and *knirps* (*kni*). The products of these genes diffuse, decay, and interact with each other to regulate gene expression. In addition gap genes are regulated by four external inputs, provided by the gene products of *bicoid* (*bcd*), *caudal* (*cad*), *tailless* (*tll*) and *huckebein* (*hkb*). Central circle: fitting the model to quantitative, spatial expression data is done via an iterative optimisation algorithm. A gene circuit with random regulatory parameters is used as the starting point for an exploration of parameter space. This is achieved by repeatedly changing parameter values to yield new gene circuit solutions. For each of these solutions model output is calculated and compared to the data by evaluating the sum of squared differences between the two. New gene circuit solutions are selected according to a global optimisation strategy (see Materials and Methods). The aim is to improve the fit to the gene expression data over many iterations until no further improvement can be achieved. Bottom panel: gene circuits that accurately reproduce expression data contain parameter estimates that encode a specific regulatory network structure. These models are analysed to yield insights into the regulatory dynamics and function of each interaction in the network.

The particular reverse-engineering approach we use to investigate the gap gene network is called the gene circuit method [1], [38], [39] (Figure 1). It uses mathematical models called gene circuits that represent the basic properties of the embryo and the transcriptional regulatory interactions underlying the network. Gene circuits are described in detail in Materials and Methods. Here we provide a brief overview of the model, which consists of a row of dividing nuclei (Figure 1, top left) each harbouring an identical version of the regulatory network. There are three processes that occur within and between nuclei: (1) regulated gene product synthesis, (2) Fickian gene product diffusion, and (3) linear gene product decay (Figure 1, top middle). Regulatory interactions that direct synthesis are represented by a genetic interconnectivity matrix: each regulatory weight in this matrix can represent activation, repression, or no interaction depending on whether it is positive, negative or (close to) zero (Figure 1, bottom panel, right). The interconnectivity matrix can also be displayed as a network diagram (Figure 1, bottom panel, left). Note that the values of regulatory weights are not set *a priori*. Instead, they are estimated by fitting the model to quantitative gene expression data. Those model solutions that fit the data well are analysed to characterize the regulatory structure and dynamics of the network (Figure 1, bottom). In this way, gene circuits act as tools to extract regulatory information from quantitative data.

Previous reverse-engineering studies of the gap gene network were based on quantitative expression data obtained by visualising the distribution of gap gene mRNA [40], or protein products [41]–[43] using fluorescent whole-mount *in situ* hybridisation, or antibody staining (immunofluorescence) respectively. Stained embryos were imaged using confocal laser-scanning microscopy, and the resulting expression profiles were quantified using a processing pipeline that includes image segmentation to identify nuclei, time classification, removal of non-specific background staining, data registration to remove embryo-to-embryo variability, and data integration (reviewed in [44]). It took years of effort by several researchers to establish the protein data set [41], [43]. mRNA data, on the other hand, were acquired by one of the authors of this paper in considerably less time [40]. However, these mRNA data remain incomplete in that they only cover a subset of gap genes (*Kr, kni*, and *gt*) during the earliest stages of expression.

These previous reverse-engineering studies have yielded many new insights into gap gene regulation, which would have been difficult to obtain by experimental approaches alone. An early pioneering study predicted a co-operative effect between maternal factors Bcd and Hb on the regulation of gap gene expression [45]. Later efforts uncovered a mechanism for the dynamic anterior shift of gap domains over time [46], [47], removed ambiguities in the published experimental evidence [48], [49], identified core mechanisms for gap gene regulation [48], [50], and explained the robustness of the system against variable levels of maternal inputs [47], [51]. Taken together, these studies clearly demonstrate the utility and feasibility of the approach: over the past two decades, reverse engineering has contributed significantly to our understanding of gap gene regulation.

It would be extremely interesting to apply the gene circuit method to other developmental systems. In our view, reverse engineering has tremendous potential for the study of gene regulatory networks in development and evolution. For instance, gene circuits could be used to reconstruct homologous developmental regulatory networks across a range of species, to compare their regulatory structure and dynamical behaviour [26]. This could be used to map which regulatory changes in a network correspond to which changes in gene expression during evolution. Alternatively, it would also be highly interesting to compare network structures and dynamics between different developmental processes.

Despite this potential, the application of dynamic, non-linear reverse-engineering approaches beyond gap genes in *Drosophila* has been very limited. The main reason for this, we suspect, is the following: collection of high-quality data sets—such as the spatio-temporal profiling of gap genes described above—is costly both in terms of time and resources. It is clearly the bottleneck of the approach. Protocols based on immunofluorescence require antibodies, which are difficult and expensive to obtain. Confocal microscopy is time-consuming and laborious, since a large number of embryo images need to be scanned. Moreover, while protocols for data acquisition and quantification work efficiently in *Drosophila*, their application to less well-established experimental models is not trivial. In particular, it is often difficult to adapt fluorescent staining protocols to non-model species.

Thus, in order to make the gene circuit method more widely applicable—and hence useful for the study of developmental gene regulatory networks—it is imperative that we simplify the method. We address an important question which applies to reverse-engineering approaches in general: how much, and what kind of data are required to successfully infer a gene regulatory network? Answering this question in the context of the gap genes will allow us to minimise the cost of data acquisition and processing. This, in turn, will decrease the barrier for applying reverse-engineering methodology to other developmental systems, many of which are similar in kind and complexity to the gap gene network.

The quality of a gene circuit model depends directly on the quality of the data it was fit to. What matters most in this regard is the timing and position of expression domain boundaries with respect to each other. The relative level of expression in each domain is less crucial. For instance, early gap gene circuit models did not capture the formation of the abdominal *kni* domain correctly (see Figure 2 in [45]). This was due to the incorrect relative position of this domain in the data resulting in a large gap between it and the posterior *hb* domain (see Figure 1, *ibid*.). This defect is no longer present in more recent models based on data with the abdominal *kni* domain positioned accurately while still only measuring relative levels of protein concentration [46], [48]–[50].

**Figure 2:**
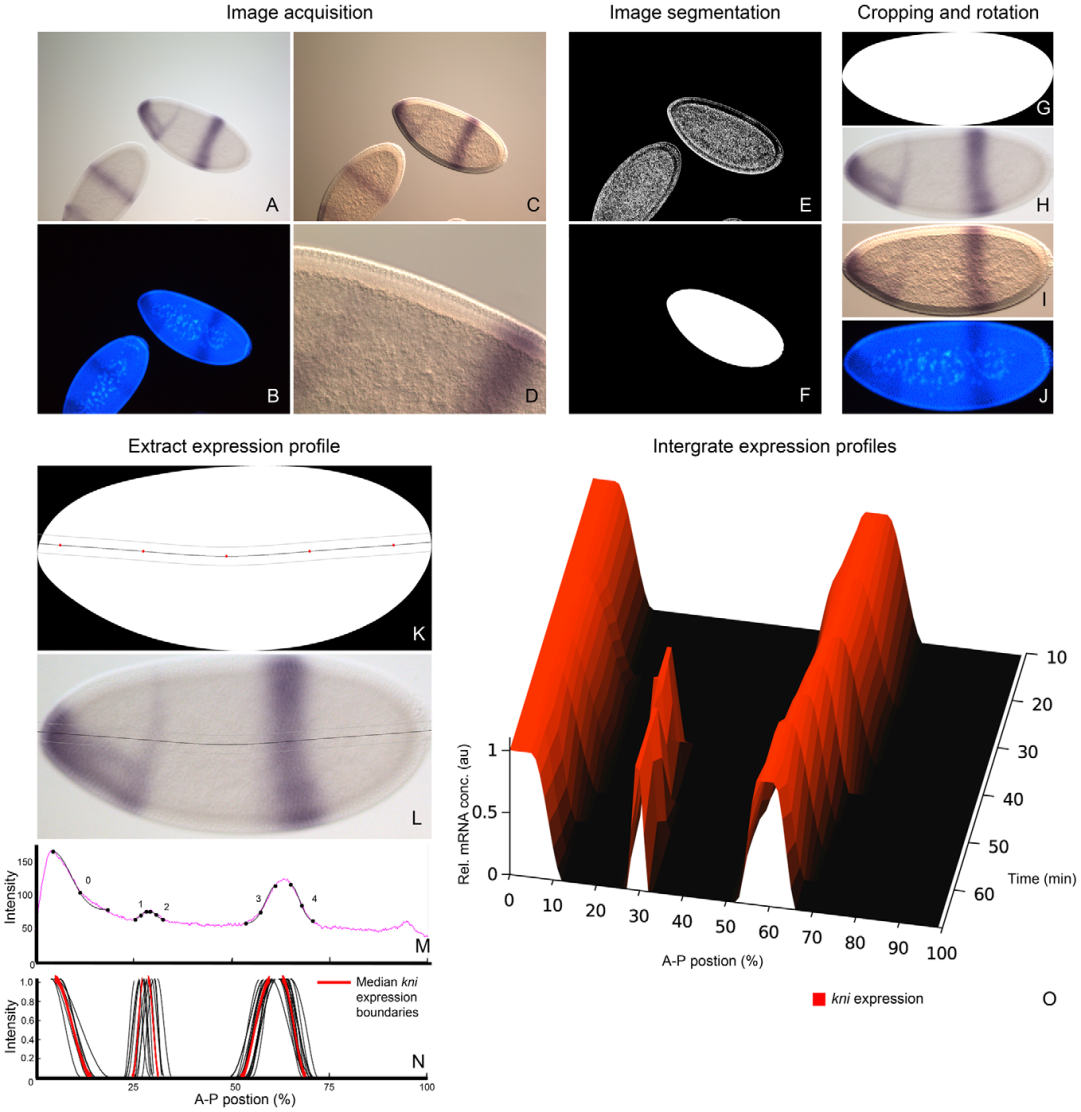
Image acquisition and processing. As an example we show quantification of *kni* expression. (A–D) Embryo images are acquired using wide-field microscopy: unprocessed bright-field (A), DAPI counter-stain (B), and DIC images (C), as well as higher-resolution details of membrane morphology using DIC (D). Whole-embryo DIC images (as shown in C) are subjected to a sequence of image segmentation steps: 1. convert the image to gray-scale, 2. adjust gamma, 3. invert image, 4. apply Sobel edge detection (E), followed by 5. dilation operations, 6. filling of holes, and 7. removal of blobs touching the image border. Only the largest blob is kept, and Gaussian smoothing is applied to generate a binary mask covering the embryo (F). Whole-embryo masks are used to crop bright field (A), DAPI (B) and DIC (C) images, which are then rotated and flipped to orient them anterior to the left and dorsal to the top (G–J). The midline of the embryo is identified by using the skeleton of the whole-embryo mask, which is then approximated by a spline curve for smoothing and pruning of superfluous skeleton branches (K). We establish a band of 10% mask height along the midline of the embryo (K), which is overlain on the bright-field image (H) as shown in (L). A raw expression profile is extracted from this band (M), which shows high and irregular non-specific background. To eliminate this background, profiles are manually annotated and expression boundaries are approximated by cubic spline curves (M). We calculate the median position of extracted boundary positions for each expression profile per time stage and normalise the data (N: boundaries from individual embryos in black, median boundary in red). Median boundaries from multiple time classes are integrated to create an expression profile along the A–P axis though developmental time (O: contour plot with interpolated data between time classes). See main text for details.

In this study, we present a simplified reverse-engineering protocol and apply it to a new, quantitative data set of gap gene mRNA expression in *Drosophila*. We demonstrate how mRNA expression data derived from a colorimetric (enzymatic) protocol for *in situ* hybridisation can be used to infer the regulatory structure and dynamics of the gap gene network. We compare our results with those obtained in previous studies based on protein expression data, and show that they predict equivalent regulatory mechanisms that are consistent with experimental evidence. In addition, we show that our simplified data set can be reduced even further while still yielding correct predictions. In this way, we define a set of minimal requirements for the successful inference of gap gene regulatory network structure and dynamics. These minimal requirements suggest that the adapted gene circuit method can be applied to a variety of developmental systems with a reasonable amount of effort. Such wider application of reverse-engineering methods will enable us to carry out systematic and comparative analyses of developmental gene regulatory networks.

## Materials and Methods

### *In Situ* Hybridisation

cDNA clones were ordered from the *Drosophila* Genomics Resource Center (DGRC; dgrc.cgb.indiana.edu) and used to make riboprobes labelled with DIG and/or FITC. Wild-type blastoderm-stage *Drosophila* embryos were collected after 4 hrs of egg laying and stained with a colorimetric *in situ* hybridisation protocol adapted from [52] and [53]. In brief, fixed and dehydrated embryos were re-hydrated by washing 1x in PBT/methanol (embryos are allowed to sink before the solution is removed), 2x in PBT, and 1×5 min in PBT. Embryos were incubated in PBT containing 0.179U proteinase K for 1 hr on ice, then washed 2x in ice-cold PBT. Embryos were post-fixed for 25 min with 5% formaldehyde in PBT with mild shaking, then washed 1x followed by 2×5 min in PBT. Embryos were pre-hybridised by washing 1×10 min in equal volumes PBT and hybridisation buffer (50% formamide, 5xSSC, 5 µg/ml yeast tRNA, 100 mg/ml salmon-sperm DNA, 50 µg/ml heparin, 0.1% tween-20 in DEPC-treated water), 1×2 min in hybridisation buffer, and 1×1 hr in hybridisation buffer at 56°C. Hybridisation was carried out overnight: 0.5–1 ng/µl of probe(s) were added after heating at 80°C in a small amount of hybridisation buffer for 3 minutes. Post-hybridisation, the embryos were washed 1×15 min and 2×30 min in hybridisation buffer, 1×15 min in equal volumes of PBT and hybridisation buffer, and 4×15 min in PBT. Blocking steps were carried out using 5% heat-treated goat serum in PBT for 30 min, followed by incubation with anti-DIG or anti-FITC antibodies conjugated with alkaline phosphatase (Roche) at 1∶2000 in 5% heat-treated goat serum in PBT for 1 hr. Unbound antibody was removed with washes of 3x followed by washes of 4×15 min in PBT. To prepare for staining, embryos were washed 2×5 min in AP buffer (100 mM NaCl, 50 mM MgCl, 100 mM Tris pH 9.5, 0.1% tween-20). Staining was carried out in the dark by the addition of AP buffer containing 0.1 mg/ml NBT and 0.05 mg/ml BCIP. Staining was stopped with 3x washes in PBT. For single staining (one probe), embryos were washed a further 3×10 min then counter-stained (see below). For double staining (two probes), alkaline phosphatase was inactivated by washing 1×1 min, then 1×10 min in glycine buffer (0.1 M glycine 0.1% tween pH 2) followed by 3×10 min in PBT. Blocking, antibody incubation and washing steps were carried out as described above. To prepare for staining, embryos were washed 2×5 min in Fast Red buffer (100 mM Tris pH 8.2, 0.1% tween-20). Staining was carried out in the dark by the addition of Fast Red solution (1 Fast Red tablet (Roche) dissolved in 2 ml Fast Red buffer). Staining was stopped with 3×1 min followed by 3×10 min washes in PBT. Nuclei were counter-stained by a 10-min incubation in PBT containing 0.3 µM DAPI, followed by washes of 3x followed by 3×10 min in PBT. Embryos were cleared through a series into 70% glycerol:PBT, of which 30 µl were mounted per slide. All washes were done on a nutator, except for those in proteinase K.

### Image Acquisition and Processing

An overview of image acquisition and processing steps is shown in Figure 2. For each embryo, four images were acquired using a compound, wide-field, fluorescence microscope: (A) a bright-field image (Figure 2A), (B) a fluorescent image of the DAPI nuclear counter-stain (Figure 2B), (C) a differential interference contrast (DIC) image (Figure 2C), and (D) a DIC image of membrane morphology on the dorsal side of the embryo (Figure 2D). Images A–C were acquired using a 10x objective, image D using a 40x objective. Images A and B are focused on the surface, images C and D on the sagittal plane of the embryo. All images were taken at 8-bit accuracy, thus setting the range per RGB channel to [0,255].

Only laterally oriented embryos were selected for processing. Gene expression patterns were extracted from embryo images as follows. Binary masks covering the whole embryo are calculated using a sequence of image segmentation steps on the DIC image (Figure 2C). Intermediate steps are shown in Figure 2E/F: 1. the RGB image is converted to gray-scale, 2. gamma correction is applied to increase contrast, 3. the image is inverted, 4. Sobel edge detection is carried out, 5. dilation operations are applied to the resulting binary image, and 6. holes are filled [54], [55]. This results in a number of contiguous binary blobs in the mask image. All blobs touching the image border are removed, and only the largest blob is retained. Finally, a smooth whole-embryo mask is created by applying a Gaussian filter to the remaining blob. This mask and all raw images (A–C) were rotated and cropped as described in [56] such that the embryo's major, or antero-posterior (A–P) axis is horizontal. If necessary, embryo images were flipped manually to a canonical orientation such that anterior is to the left, and dorsal is up (Figure 2G–J).

To extract gene expression profiles from an embryo, a smooth cubic spline was generated with five equidistant knots through the main branch of the skeleton of the embryo mask (Figure 2K; [55], [57]). Along the spline, we extract average RGB-values over a band of 10% mask height (5% above and below the spline curve), resulting in raw profiles for each RGB channel of the bright-field image (Figure 2H) along the A–P axis (Figure 2K,L). Values for FastRed or NBT/BCIP staining were then calculated as follows: FastRed = green − red, and NBT/BCIP = red, where ‘red’ and ‘green’ refer to inverted RGB colour channels extracted from the bright-field image. Inspecting 1D-graphs of the resulting profiles (Figure 2M), boundaries for gene expression domains were extracted manually for each embryo: each boundary was labelled with a unique identification number (see Supplementary Material), and two points (x_0_, y_0_) and (x_2_, y_2_) were determined that indicate the beginning and end of the boundary, where staining levels approach background and maximum levels respectively. A middle, third control point (x_1_, y_1_) was automatically calculated from the other two points by taking the average for x, and locating the corresponding expression level y. Hence 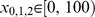 represent relative position along the A–P axis (in percent, where 0% is the anterior pole), and 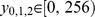 represent the relative intensity level of the staining. Points x_0,2_ and y_0,2_ were used as anchor points for cubic splines with fixed zero-derivatives at their end knots. Finally, splines were normalised such that the expression level at the starting point was 0, and the expression level at the end point was 1 (Figure 2N).

Integrated time-series of gene expression were prepared as follows. Embryos were staged into separate cleavage cycles (defined as the period between mitotic divisions *n-1* and *n*; e.g. cycle 13 spans the time between mitoses 12 and 13) based on nuclear density and number of nuclei in images showing DAPI nuclear counter-stains (Figure 2B; time classification for early embryos was described previously in [40]). C14A was further subdivided into eight equally spaced time classes (T1–8) based on membrane morphology from high-resolution DIC images (Figure 2D; time classification for late embryos as described in [44]). Expression domain boundaries were grouped by gene, stage and boundary identification number (see above). Average boundary positions were determined by calculating separately the median start and end points for each group, which were then used for fitting a median-boundary spline as described for individual boundaries above. Finally, we combined different domain boundaries for each gene at each time class into an integrated, normalised expression profile along the A–P axis (Figure 2O). Spatial registration of domains was performed by checking the integrated expression profiles against double stained embryos (i.e. embryos stained for two gap genes, for instance *hb* and *Kr*) to verify the relative spatial order of gap gene expression domains (data not shown).

### Additional Data Processing for Model Fitting and Comparison

The following post-processing steps had to be applied to our data to make them suitable for model fitting and comparison (see Results, and Figure S1 of the online Supporting Information): (1) We collected our normalised, integrated mRNA expression data into 50 (C13) or 100 (C14A) bins to reflect the approximate number of nuclei along the A–P axis [46], [48], [49]. (2) We scaled the intensity of our expression data linearly along the A–P axis from 50% A–P position (mid-embryo; ×1.0) to both termini (poles of the embryo; ×0.5) to reflect the higher intensity of central versus more terminal gap domains [43]. For *Drosophila* expression data, this is a reasonable assumption, but not an essential requirement. Omitting this post-processing step resulted in qualitatively equivalent results (data not shown). (3) We also scaled our expression data along the time axis by a second-degree spline with a peak of expression during early C14A, to capture the gradual accumulation (during C12, C13 and early C14A) and degradation (during late C14A) of gap gene mRNA ([40], [43], and our unpublished data). Normalised boundaries were scaled to 0.1 at the onset of expression (early C13, *t = 0.0 min*), 1.0 at around T5 (*t = 48.0 min*), and 0.7 at gastrulation time (*t = 71.1 min*), which is the final time point. (4) We multiplied our mRNA expression data by a constant factor of 200. This makes the scale of both mRNA and protein data match as closely as possible, and therefore facilitates comparison to models obtained with *Drosophila* protein data [46]–[51].

The posterior *Kr* domain, which arises in late C14A, was removed from the data used for model fitting to avoid modelling artefacts. This domain is known to be under regulatory control of the terminal gap genes with additional inputs from the Forkhead (Fkh) transcription factor (not included in this study), and it does not participate in segment determination [58].

Fitting models using a weighted least squares (WLS) protocol (see below) requires a weight for each data point indicating its associated variation. As our mRNA expression data do not provide such information, weights were created from normalised, integrated mRNA expression data according to the formula: 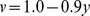 with 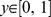 being the normalised staining intensity and *v* the corresponding weight. This proportionality of variation with expression level reflects the fact that gap domains (showing high levels of expression) show more variation than those regions of the embryo in which a gene is not expressed [40], [43].

Gene circuits used for model fitting require external inputs based on data for maternal gradients (Bcd and Cad) as well as terminal gap genes (Tll and Hkb). Depending on the scenario we wanted to test (see Results), expression profiles for these inputs were taken from previously published quantitative protein data [43], [49], or were approximated as follows. Bcd: a time-independent (i.e. constant) anterior gradient was created by fitting an exponential curve to all available Bcd data across time and space. Cad: an artificial posterior morphogen gradient was created using 2D thin-plate splines [59] based on the following minimal set of features of the Cad protein gradient: (1) the gradient should be complementary to the anterior (Bcd) gradient; (2) as development progresses, there should be increasing repression in the abdominal region (∼50–80% A–P position); and (3) a posterior stripe should develop from time class 6 in C14A onwards at around 80% A–P position. Tll/Hkb: we replaced protein expression data with mRNA expression data for some of our reverse-engineering runs (see Results). Due to the relative constancy of *tll* and *hkb* expression patterns over time [43], [49], we created time-invariant expression profiles for these genes by averaging boundary positions across all cleavage cycles and time classes.

### Mathematical Models: Gene Circuits

We use gene circuits for model fitting (reverse engineering) as described in [1], [38], [39], [46]–[51]. In brief, a gene circuit is a hybrid dynamical model incorporating discrete mitotic divisions of nuclei, as well as continuous gene regulatory dynamics within each nucleus. Each cleavage cycle consists of interphase, mitosis and division. During interphase, the change in gene product concentration 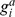 (representing mRNA or protein, depending on the simulation) for gene *a* in nucleus *i* over time *t* is governed by the following of ordinary differential equation (ODE)
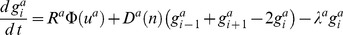
(1)The three terms on the right-hand side of the equation define regulated gene product synthesis, diffusion and decay respectively. 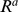 is the maximum synthesis rate; 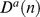 the diffusion rate (dependent on the distance between nuclei, which halves at every cleavage division: *n* defines the number of previous divisions); 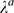 is the decay rate for the product of gene *a*. The sigmoid regulation-expression function 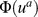 captures the basic regulatory dynamics, and is defined as follows:
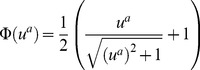
(2)where
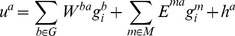
(3)with the set of trunk gap genes defined as 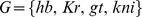, and the set of external inputs as 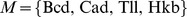. Matrices *W* and *E* define the interactions between, respectively, the trunk gap genes themselves, and between the external inputs and trunk gap genes. The elements of these matrices, *w^ba^* and *e^ma^*, are called regulatory weights. These weights define the effect of *b* on *a* (or *m* to *a*) which can be (1) positive (activating gene product synthesis), (2) negative (inhibiting synthesis), or (3) (close to) zero (no regulatory interaction). *h^a^* is a threshold parameter representing uniformly distributed maternal factors. During mitosis, gene product synthesis is set to zero. After mitosis follows division, which is instantaneous. At division, gene product concentrations are copied equally to both daughter nuclei. Finally, diffusion is implemented with no-flux boundary conditions.

Our models cover the trunk region of the embryo, from 35 to 87% A–P position. This region is somewhat reduced compared to 35 to 92% for earlier protein models [46]–[51], but covers the same set of gap domains, due to the slightly more anterior position of mRNA vs. protein domains. This results in gene circuits consisting of systems of 108 ODEs at C13, and 212 ODEs at C14A. Gap gene circuits were solved numerically from the beginning of C13 (*t = 0 min*) when gap proteins reach detectable levels, to the onset of gastrulation and the end of C14A (*t = 71.100 min*). We use the same division schedule as in [46], [48], [49]: mitosis occurs from 16.0 min to 21.0 min. At the end of mitosis, nuclear division takes place. Initial conditions for gap genes were calculated by interpolation between data points at C12 (*t = −6.200 min*) and C13 (*t = 10.550 min*) using the same temporal scaling scheme as described in the previous section. Initial conditions of the external inputs were taken from [49]. Time classes in C14A correspond to the following time points (in minutes): T1, *t = 24.225*; T2, *t = 30.475*; T3, *t = 36.725*; T4, *t = 42.975*; T5, *t = 49.225*; T6, *t = 55.475*; T7, *t = 61.725*; T8, *t = 67.975* (see Figure 2 in [48]).

### Model Fitting

We follow a reverse engineering protocol as described in [46], [48], [49]. To estimate the values for parameters *W, E, R, h, D* and *λ* of the gene circuit model we performed global optimisation by means of parallel Lam Simulated Annealing (pLSA) [60]–[62] on the Mare Nostrum supercomputer at the Barcelona Computing Center (BSC; http://www.bsc.es). Per optimisation run we used 50 processor cores for an average duration of about 7 hours.

Simulated Annealing requires that candidate solutions have an associated cost (or energy) function that is minimised during the optimisation. We adapt the cost function from [49] as follows:
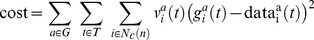
(4)with *T* the set of time points (C13, C14A: T1–8) at which expression data is available, *N_c_(n)* the number of nuclei after n divisions (50 at C13, 100 at C14A), 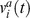 representing positive weights associated with each data point, and 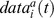 referring to the expression level of gene product *a* at nucleus *I* and time *t* as derived from the experiments. If weights 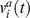 are all set to 1.0, the ‘cost’ equation represents a fit by ordinary least squares (OLS), which is the cost function previously used with gene circuit models [46]–[51], [63]. For fitting by weighted least squares (WLS), we use variable weights, which are inversely related to the level of expression (calculated as described in ‘Additional Data Processing for Model Fitting and Comparison’). This penalises ectopic gap gene expression, and improves frequency and quality of good fitting solutions as reported in [49].

Based on previous studies using gap gene circuit models, we fix certain parameters without negatively affecting the quality of the fits [46], [48], [49]. In the gene interaction matrix *E* we fix interactions of Hkb to zero, with the exception of Hkb→*hb*. Furthermore, we take *h^a^* = −2.5, for all gap genes 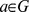, and 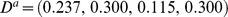 for 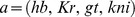 respectively [49].

To report the goodness of a fit, we use the root mean square (RMS), defined by
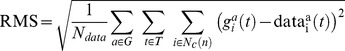
(5)with 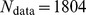 the total number of data points in our data set. Since the RMS is independent of weights 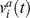 and the number of nuclei (data points) in the model, it allows us to compare WLS and OLS, as well as mRNA- and protein-based solutions. Solutions were selected for further analysis by several tests. Firstly, gene circuits were tested for numerical stability with respect to the solver (also known as solver sensitivity) and with respect to minute changes in parameter values (or the ‘brittleness’ of a solution). Subsequently, gene circuits were checked for visible gene expression patterning defects by means of visual inspection (see Text S1 for a categorisation of commonly encountered defects).

### Statistical Analysis of Parameter Estimates

Statistical analysis of parameter estimates was performed as described in [49], [64]. Here we only provide a short description of the calculation of parameter confidence intervals.

Reverse engineering results in a vector of estimates 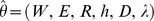. Once the parameter vector 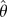 is found, a posteriori identifiability analysis [65]–[67] reveals how reliable the obtained estimate is. The ellipsoidal region around 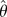 in which the true parameter vector 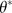 lies with a certain probability 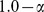 (we set 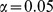) is defined by
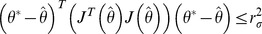
(6)where
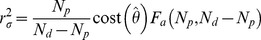
(7)and 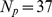 and 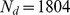 the number of parameters and data points, respectively. 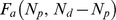 is the upper 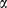 part of Fisher's distribution with 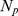 and 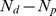 degrees of freedom. *J* is the Jacobian matrix of size 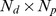 defined as 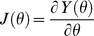, where 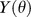 is the vector of weighted discrepancies between model output and data. From equation 6 one can derive dependent and independent confidence intervals for each parameter estimate 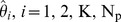. These are, respectively,
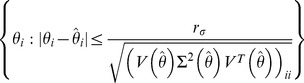
(8)and
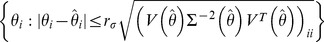
(9)Here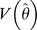 and 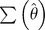 are obtained from the singular value decomposition of 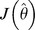. It is well known that in the presence of strong correlations between parameters, the dependent confidence intervals underestimate the confidence region while the independent confidence intervals overestimate it. For detailed explanations of these statistical quantities and their derivations we refer the reader to [49], [64], and references therein.

### Database and Code Implementation

Image processing and extraction of expression domain boundaries were performed using a custom-made processing pipeline with a graphical user interface developed in Java (using the ImageJ API; http://rsbweb.nih.gov/ij). Intermediate processing steps and domain boundary positions were stored in a MySQL database, with a web interface (SuperFly) developed by the CRG Bioinformatics Core Facility. SuperFly is available online at: http://superfly.crg.es. We used scripts written in Python, Perl and R for the preparation of integrated data sets, for the generation of artificial external inputs, and for analysis of gene circuit models. Code for numerical solution and optimisation of gene circuits by pLSA [39], [45]–[49], [51], [62] is implemented in C, using the GNU Scientific Library (GSL, www.gnu.org/software/gsl), the Sundials ODE Solver Library [68], and the Open MPI message-passing interface (www.open-mpi.org). For numerical integration of ODEs, we use an implicit variable-order, adaptive-stepsize, multi-step method; a band-direct solver calculates the set of equations that is generated at each integration step [68].

## Results

### Gap Gene Expression Patterns: mRNA vs. Protein

Over the last two decades, the potential of reverse engineering has been demonstrated by a pioneering case study—led by John Reinitz and colleagues—where gene circuits have been used to characterise and analyse the gap gene network in *Drosophila melanogaster* [40], [45]–[51]. Despite this, the gene circuit method has not yet been applied more widely. One reason for this is that it took many years to establish the required quantitative data set of spatial gap protein expression patterns [41]–[43].

We have developed a simplified protocol for data acquisition and processing, which allows us to create a quantitative data set of spatial gene expression patterns in a time span of months rather than years. Instead of using protein expression data, we have quantified mRNA expression patterns by colorimetric (enzymatic) *in situ* hybridisation, imaged using a wide-field, compound fluorescence microscope. The resulting data set is of reduced quality compared to the original protein data. Here, we address the question whether it can still be used to reconstruct the regulatory structure and dynamics of the gap gene system in a manner which is consistent with previous efforts based on modelling, as well as genetic and molecular approaches to study gap gene regulation.

**Figure 3:**
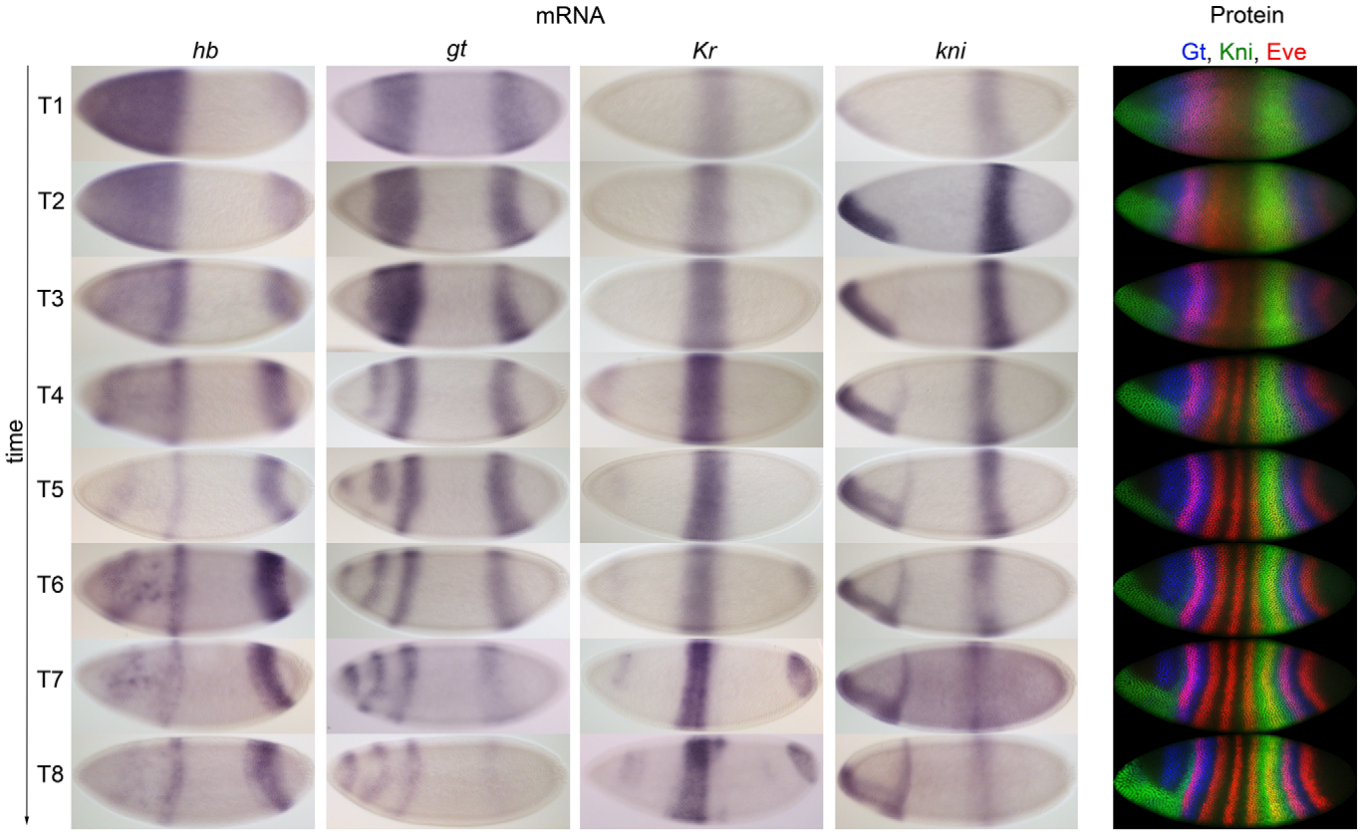
Comparison of mRNA and protein expression data: embryo images. Columns 1–4: time series of mRNA expression patterns of the trunk gap genes *hb, gt, Kr* and *kni* at cleavage cycle 14A (C14A) visualised by colorimetric (enzymatic) *in situ* hybridisation using wide-field microscopy. Column 5: time series of protein expression patterns of Gt, Kni and the pair-rule gene Even-skipped (Eve) at the equivalent developmental stages visualised by immunofluorescence using confocal laser scanning microscopy. Early stages at the top, time progresses downwards. Embryos are arranged anterior to the left, dorsal up. Embryos in column 5 are from the FlyEx database: http://urchin.spbcas.ru/flyex [69], [70]. T1–8 indicate time classes subdividing C14A (see Materials and Methods for details).

**Figure 4:**
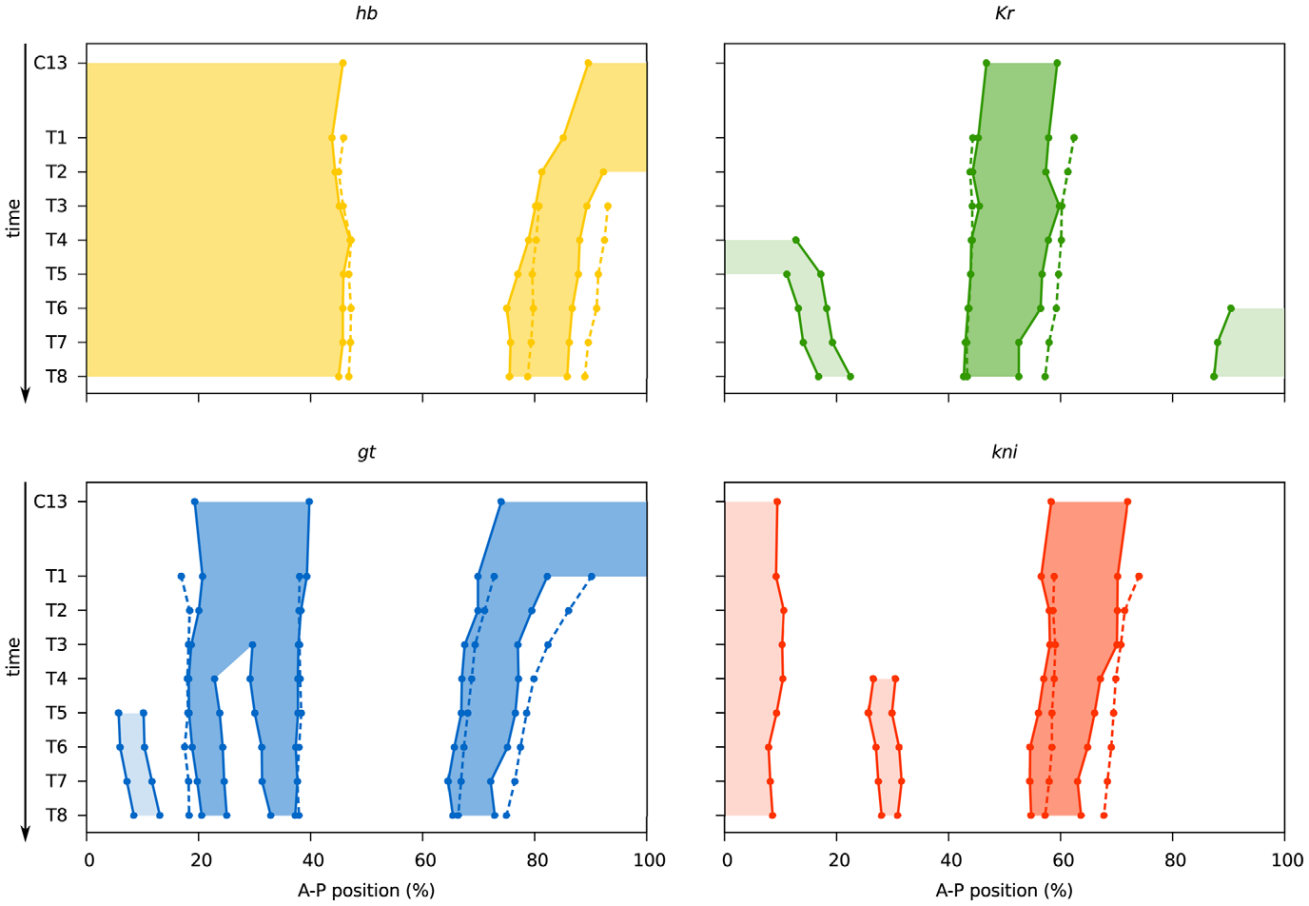
Comparison of mRNA and protein expression data: quantitative graphs. Space-time plots are shown indicating gap domain boundary positions based on mRNA (solid lines, shaded background) and protein data (dashed lines). Boundary positions for mRNA domains were determined as described for Table 2 (see also Materials and Methods). Boundary positions for gap protein domains are taken from [43]. Light shaded background indicates domains (the anterior domain of Kni, the anterior and posterior domains of Kr, and the head patch of Gt), which do not play a role in segment determination [35], and where not quantified from protein data in [43]. See also Table 2, and Tables S1 and S2.

Before we present our modelling results, we provide a quantitative characterisation of our mRNA data, and compare them to the gap gene protein expression data described in [43]. Table 1 shows the number of embryos on which our mRNA data are based. Figure 3 illustrates the quality and resolution of embryo images underlying the two data sets. It shows time series of mRNA expression patterns for the trunk gap genes *hb, gt, Kr*, and *kni* produced with a colorimetric (enzymatic) *in situ* hybridisation protocol (columns 1–4 on the left), in comparison to protein expression data for Gt, Kni and the pair-rule protein Even-skipped (Eve) from the FlyEx database (http://urchin.spbcas.ru/flyex; [69], [70]; column 5 to the right). We used images such as the ones shown in columns 1–4 of Figure 3 to quantify the position of gap gene domain boundaries across space and time. This was done by applying the image-processing pipeline as described in Materials and Methods. In brief, we used texture-based image segmentation to create whole-embryo masks, which were used to rotate and crop embryo images. The developmental stage of each embryo was determined based on numbers of nuclei and membrane morphology as previously described [44] (see also Materials and Methods). We then extracted raw profiles of gene expression within a 10% strip along the embryo's lateral midline, and we determined the position of expression domain boundaries. The results of this analysis are shown in Table 2 and Figure 4. A detailed description of gap gene mRNA patterns can be found in Text S2.

**Table 1:** Overview of the full mRNA data set.

Time class	Domain and Boundary										
	*hb* anterior	*gt* anterior		*Kr* central		*kni* abdominal		*gt* posterior		*hb* posterior	
	P	A	P	A	P	A	P	A	P	A	P
**C13**	31	8	8	9	6	16	14	11	-	1	-
**T1**	15	14	14	10	7	15	13	11	10	5	-
**T2**	13	8	10	6	5	19	16	8	9	8	1
**T3**	16	11	15	9	5	17	15	14	17	11	6
**T4**	7	11	12	10	4	18	17	9	13	10	7
**T5**	12	16	17	16	11	18	17	14	14	10	8
**T6**	9	14	14	17	15	15	15	14	12	11	14
**T7**	5	8	8	11	11	5	5	8	6	10	9
**T8**	8	12	12	6	6	6	6	7	6	9	8

The number of embryos used to calculate median positions for each expression boundary at each time point is shown. A: indicates anterior, P: posterior boundary of a domain. T1–8 indicate time classes subdividing C14A. See Materials and Methods for details.

**Table 2:** Comparison of mRNA and protein expression data: gap domain boundary positions.

Time class	Domain and Boundary										
	*hb* anterior	*gt* anterior		*Kr* central		*kni* abdominal		*gt* posterior		*hb* posterior	
	P	A	P	A	P	A	P	A	P	A	P
**C13**	45.8	19.3	39.8	46.7	59.4	58.3	72.0	74.0	-	89.5	-
	––	––	––	––	––	––	––	––	––	––	-
**T1**	43.8	20.7	39.3	45.3	57.8	56.5	70.1	69.8	82.2	85.0	-
	45.9	16.9	38.0	44.3	62.4	58.8	74.0	72.7	90.1	––	-
**T2**	44.4	20.0	38.2	44.3	57.3	57.9	70.1	69.8	79.4	81.2	92.2
	45.0	18.4	37.9	43.8	61.3	58.6	71.4	71.0	86.0	––	––
**T3**	45.1	18.7	37.8	45.5	59.8	58.0	70.0	67.5	76.9	80.1	89.3
	45.8	18.2	38.0	44.2	60.2	59.0	70.7	69.3	82.3	80.7	93.0
**T4**	47.0	18.2	37.7	44.0	57.8	56.9	67.1	67.0	77.0	78.9	88.0
	47.2	18.0	38.1	44.2	60.1	58.8	69.8	68.7	79.8	80.2	92.4
**T5**	45.9	18.3	37.7	43.9	56.7	56.0	66.0	66.8	76.5	76.9	87.7
	46.8	18.1	38.3	43.9	59.6	58.4	69.4	68.0	78.5	79.5	91.3
**T6**	45.8	18.8	37.3	43.5	56.4	54.5	64.8	65.6	75.1	75.0	86.6
	47.2	17.5	37.9	43.6	59.2	58.4	69.0	67.3	77.5	79.7	91.0
**T7**	45.8	19.7	37.6	43.0	52.5	54.5	63.0	64.5	72.1	75.6	86.1
	47.1	18.2	37.6	43.2	57.9	57.9	68.3	66.8	76.4	79.3	89.5
**T8**	45.0	20.5	37.2	42.7	52.5	54.7	63.6	65.3	72.8	75.5	85.8
	46.8	18.3	37.9	43.3	57.2	57.2	67.7	66.3	74.9	78.7	88.9

This table shows mRNA (grey rows) and protein (white rows) boundary locations through developmental time in percent A—P position (where 0% is the anterior pole). A: indicates anterior, P: posterior boundary of a domain. T1—8 indicate time classes subdividing C14A. Boundary positions for mRNA domains correspond to the starting points of approximating splines as described in Materials and Methods. Boundary positions for protein domains are taken from [43], and correspond to the position where the level of gene expression reaches a threshold of 50% maximum fluorescence intensity. Single dashes indicate boundaries that are not present at a give time point. Double dashes indicate boundaries that are observable, but were not measured in [43].

As mentioned in the Introduction, we are mainly interested in the dynamics of gap domain boundary positions across space and time. Figure 4 compares those dynamics between mRNA and protein data. It is evident that mRNA expression patterns resemble those of proteins closely. The relative position of all domains with respect to each other is preserved at all time points. Anterior shifts in domain positions over time also mirror each other between mRNA and protein data: while mRNA domains are always more anterior than protein domains at equivalent stages (as reported in [46], Figure 3), the extent of the domain shifts is similar between the two data sets (see Table S1). Finally, domain sizes are similar as well (see Table S2), although protein domains of the gap genes are slightly larger than those of their transcripts.

However, there are also notable differences between mRNA and protein data. First, all mRNA patterns arise earlier than those of their corresponding proteins: mRNA expression of all gap genes is initiated before C13 ([40], and references therein), and gap mRNA domains are well established at early cycle 13. In contrast, protein levels have only just begun to be detectable, and increase rapidly, during that stage [43]. This is due to the delay caused by mRNA processing, nuclear export, and translation. Similarly, there is an evident lag between shifting positions of gap domains (see also Figure 3 in [46]), again indicating a significant delay between the dynamics of mRNA and protein patterns. In summary, while overall expression dynamics are similar between mRNA and protein, both the timing of expression and the absolute positions of gap domain boundaries differ between the two data sets.

### Gap Gene Circuits from mRNA Expression Data

The main aim of this study is to show that the gene circuit method—originally developed for protein expression data—can be adapted to work successfully with expression profiles derived from mRNA. To achieve this, gene circuit models were fit to mRNA expression patterns derived from our data set of boundary positions for *hb, gt, Kr*, and *kni*. External inputs to the model—regulatory contributions from maternal gradients Bcd and Cad, as well as terminal gap genes *tll* and *hkb*, which are not themselves regulated by trunk gap genes—were calculated based on protein data as described in Materials and Methods.

Our models run from early cleavage cycle 13 (C13), when gap proteins start to accumulate, to the end of C14A, when gastrulation starts. They span the trunk region of the embryo from 35 to 87% A–P position. This region is located slightly more anteriorly and is somewhat smaller than that used previously for protein models (35–92% A–P position [46], [48]–[50]). However, it covers the equivalent set of gap gene expression patterns, since mRNA domains are located more anteriorly and are slightly less wide than their corresponding protein domains (see Figure 4, and Tables 2 and S2). Fitting gap genes to the original range of 35–92% A–P position yielded equivalent results to those reported below (data not shown).

The quality of a gene circuit depends crucially on the relative position and dynamics of gap domain boundaries, while levels of expression are of secondary importance (see Introduction). However, there are two problems with the loss of information on gap gene product concentrations due to the way our data were processed. The first is that, after data normalisation, boundaries in our data set appear suddenly, without gradual build-up of gene product levels (see, for example, Figure 2O). This is clearly unrealistic, and leads to problems with the numerical stability of our gene circuit models, since very short time scales (and hence, very high production rates) will be favoured by the model-fitting procedure (data not shown). We have addressed this problem by re-scaling the data over time using a second-degree spline with a peak of expression at early C14A, capturing the gradual accumulation of mRNA during C13 and early C14A, as well as its degradation during late C14A ([40], [43], and our unpublished data). The second problem concerns the relative timing and intensity of expression in different gap gene domains. While central gap domains arise early, and show intense staining levels by C14A, more terminal ones arise later, and show less intense staining [43]. For instance, the anterior mRNA domain of *hb* arises at C9 or C10 from the maternal Hb gradient, while the posterior *hb* domain only appears at C13, with much lower intensity of expression [71]–[74]. We have addressed this problem by re-scaling the data along the A–P axis, using a scaling factor of 1.0 in the middle of the embryo (at 50% A–P position) that linearly decreases to 0.5 at the poles (0 and 100% A–P position). Note that this scaling step is justified by our prior knowledge of the gap gene system, but is not strictly required to generate the results we present below (see Discussion). Finally, data were multiplied by a factor of 200, and collected into 50 bins (at C13) and 100 bins (at C14A) along the A–P axis to facilitate comparison with models based on protein data (see Materials and Methods and Figure S1 for details).

We used both ordinary (OLS) and weighted least-squares (WLS) fits to our full mRNA data set (see Materials and Methods). On one hand, the WLS method has the advantage of penalising ectopic expression outside the observed gap domains, and has been shown to be more effective than OLS for protein-based circuits [49]. On the other, it requires us to calculate the variance of each data point in our data set, which is not an obvious task given our data-processing methods. For this reason, we had to estimate weight values for the WLS cost function based on the observation that variance is higher in regions with high levels of gene expression than in those that show low, or now expression at all (see Materials and Methods for details).

**Figure 5:**
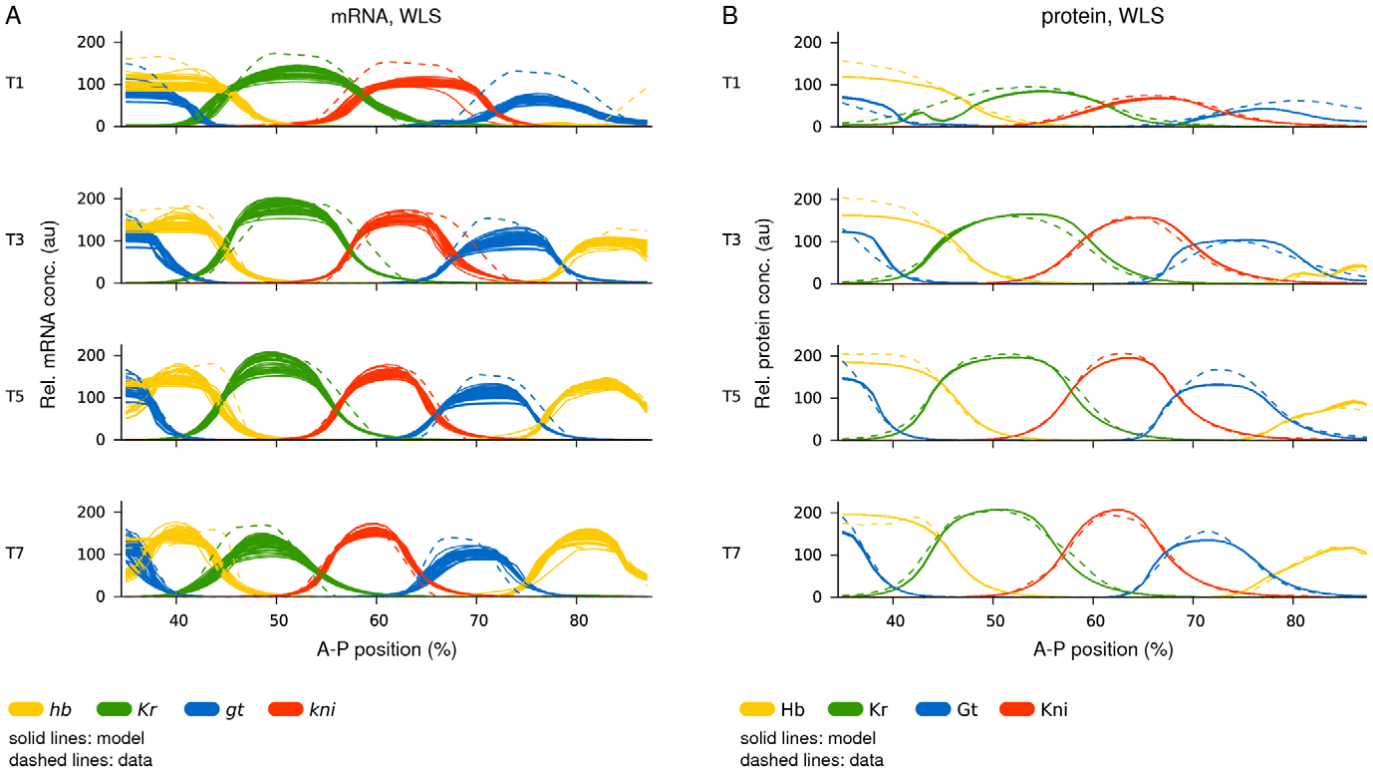
Gene circuit fits: model output versus data. Plots show model output (solid lines) versus expression data (dashed lines). (A) Gene circuits fit with WLS to mRNA data. All 52 selected gene circuits are shown. (B) Gene circuits fit with WLS to protein data (from [49]). All 66 selected gene circuits are shown. Horizontal axes represent A–P position (where 0% is the anterior pole). Relative mRNA and protein concentrations are in arbitrary units (au). T1/3/5/7 represent time classes during C14A; time progresses downwards.

We performed 150 fitting runs each with OLS and WLS cost functions respectively. Because of potential artefacts caused by overfitting, one cannot simply select those circuits with the lowest residual scores for further analysis. Instead, we inspected all solutions visually to detect obvious patterning defects (observed defects are described in Text S1). Only solutions without defects were selected. Using the WLS cost function resulted in a much higher fraction of runs with good fits to the mRNA expression data compared to OLS. For OLS solutions, only 10/150 (6.7%) were suitable for further analysis (Figure S2). Their residual errors—as measured by the RMS score (defined in Materials and Methods)—range from 16.6 to 20.9, with a median value of 17.453. For WLS solutions, 52/150 (34.7%) could be retained (Figure 5A). As is usual for WLS fitting [49], they show slightly higher RMS scores—between 20.0 and 21.7, with a median value of 21.052.

### Equivalence of mRNA- and Protein-Based Circuits

Overall, the selected gene circuits are extremely similar for both OLS and WLS (compare Figure 5A to Figure S3). Both show correct relative timing and positioning of gap domains, and reproduce the positional shifts of posterior domains towards the anterior. However, there are slight inaccuracies concerning the appearance and placement of domain boundaries. The formation of all gap domains, but particularly the posterior *hb* domain, is slightly delayed (Figure 5A, T1), and several boundaries are offset by 1–3 nuclei at specific points in time compared to the data (see, for example, the anterior boundary of *kni* at T1, and its posterior boundary at T3 in Figure 5A). These slight defects result in patterns that reproduce the data less faithfully than those obtained by WLS fits to protein data (Figure 5B; models from [49]). This is reflected in the corresponding RMS scores: 10.5 for protein data versus 16.6, the best score from our mRNA fits. In addition, our mRNA-based circuits show increased variability in model output between solutions compared to protein-based models (Figure 5, Figure S3).

**Figure 6:**
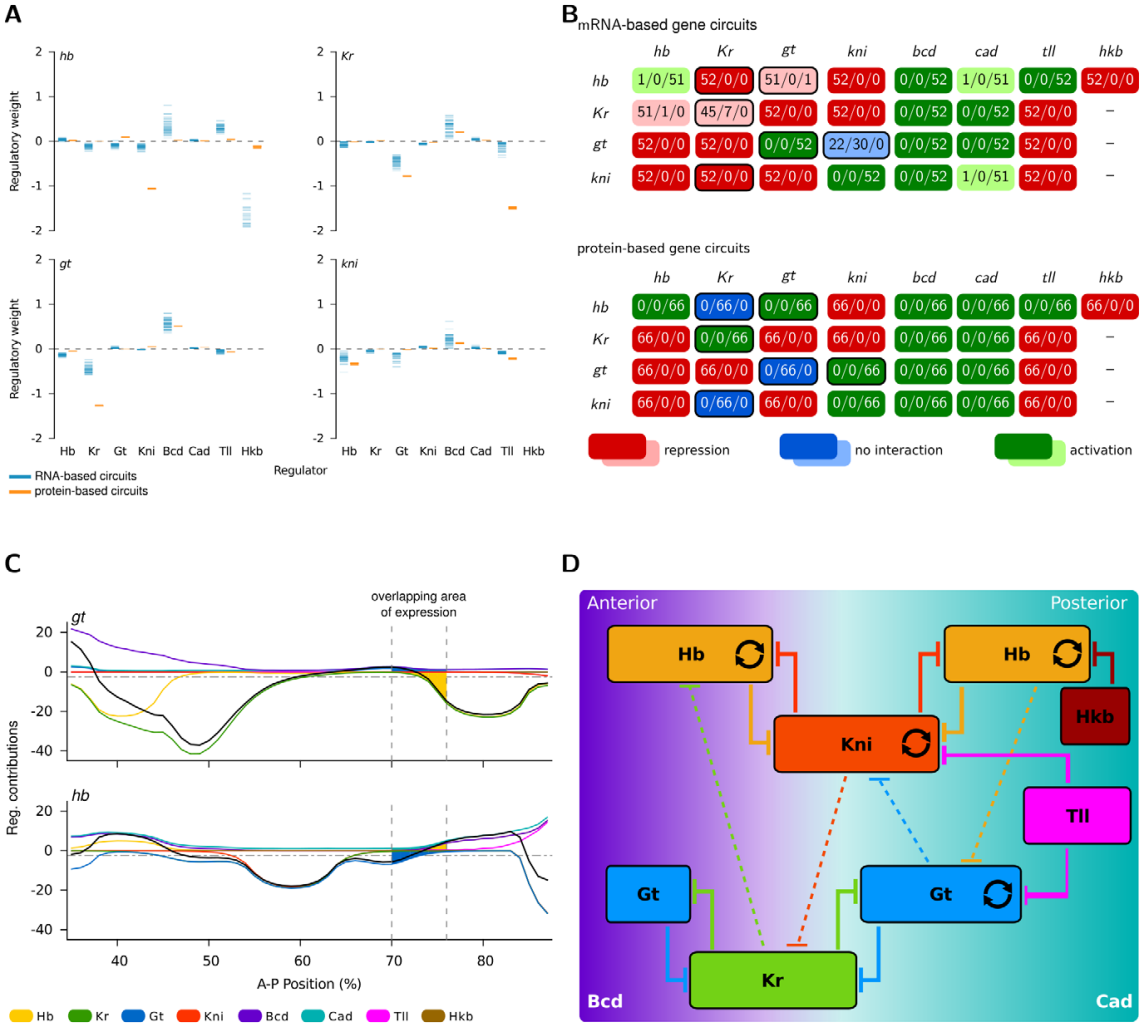
Gene circuit analysis: mRNA- versus protein-based models. (A) Distribution of estimated parameter values (shown as scatter plots) for regulatory weights. Horizontal bars indicate the strength and type of interaction between regulators (indicated along the x-axes of the plots) and their target genes (sorted into separate plots). Blue represents parameter values from mRNA-based circuits, orange parameter values from protein-based circuits. (B) Genetic interconnectivity matrices indicating repressive (red), activating (green), or no (blue) interactions for WLS mRNA- (top) and protein-based gene circuits (bottom). Number triplets indicate the number of solutions with repressive/no/activating interactions. As in earlier studies [48], [49] a cut-off of 0.005 was used below which an interaction was considered to be absent. In cases where all gene circuits show identical behaviour, a dark colour is used; otherwise a lighter shade is applied. A black border highlights differences in interactions between the mRNA and protein circuits. (C) Regulatory contributions (calculated as regulator concentrations 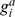 multiplied by regulatory weights 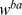 or 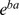 see equation 3) of gap genes and external inputs on *gt* (upper panel) and *hb* (lower panel) along the A–P axis (0% is the anterior pole). The horizontal dashed lines indicate threshold values (parameter 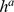 in equation 3). The sum of positive contributions (above dashed line) is plotted separately from the sum of negative ones (below dashed line). The black curve indicates total regulatory effect: it is activating above the dashed line, and repressing below the dashed line. The posterior area of overlap (between vertical dashed lines) indicates where *gt* and *hb* have overlapping expression domains (the anterior overlap is omitted for clarity). To calculate the net interaction between *gt* and *hb*, the respective regulatory contributions are integrated over this area and the difference is calculated. The size of the coloured areas indicates a stronger repressive effect of Hb on *gt* than the other way around. (D) Consensus gap gene network recovered by both mRNA- and protein-based circuits. The position of gap domains is shown schematically along the A–P axis (coloured boxes). Background colour indicates predominant maternal activators. Circular arrows indicate auto-activation, T-bar connectors represent major repressive interactions, and dashed T-bar connectors show asymmetric repressive interactions determined by net effect (see panel C). See text for details.

Apart from these minor differences, however, there is significant agreement between all three sets of gene circuits. This similarity in expression patterns is reflected in the parameter values of our models. Distributions of estimated parameter values for regulatory weights of WLS-mRNA and WLS-protein solutions are shown as scatter plots in Figure 6A. Corresponding genetic interconnectivity matrices are compared in Figure 6B. It is clear from inspecting these matrices that a large majority of interactions are qualitatively the same in mRNA- and protein-based circuits (although increased variability in model output is reflected in increased variability of estimated parameter values for mRNA circuits). Repression corresponds to repression, and activation to activation, for a majority of circuits in both cases. Only six gap-gap cross-interactions are predicted to be different (emphasised by black frames in Figure 6B, and discussed in detail in Box 1), while all external inputs (*bcd*, *cad*, *tll* and *hkb*) interact with gap genes in the same manner for both data sets.

**Box 1. Detailed Analysis of Differences Between mRNA- and Protein-Based Circuits**

We analysed the differences between mRNA- and protein-based gene circuits in detail. Surprisingly, one of the differences is an improvement: our mRNA circuits predict repression of *hb* by Kr, which is in accordance with the experimental literature [77], [95]–[97]. This interaction had been missing in protein-based circuits. On the other hand, the two differences affecting the auto-regulatory terms of *Kr* and *gt* are of no functional significance, since auto-regulation is dispensable for correct gap gene expression [50].

The final three differences require more explanation: mRNA circuits predict Kr to inhibit *kni*, Kni to not interact with *gt*, and Gt to repress *hb*, while protein-based circuits predict no interaction for Kr on *kni*, and activation in the other two cases (Figure 6B). Of the mRNA interactions, only the model outcome of no interaction between Kni and *gt* is supported by experimental evidence, as the anterior boundary of the posterior *gt* domain is not affected in *kni* mutants [98]–[100]. With respect to the other two interactions, we know the effect of Kr on *kni* is an indirect one via the de-repression of *gt*. Also, the posterior *hb* domain appears normal in embryos over-expressing or mutant for *gt* [99], [101], [102]. In accordance with this, both mRNA- and protein-based circuits predict that the effect of Kr on *kni*, Kni on *gt*, as well as that of Gt on *hb*, is extremely weak (Figure 6A, and [48], [49]). Note that about 40% of mRNA-based circuits show an inhibitory effect of Kni on *gt* (Figure 6B). This suggests that it does not matter too much whether these interactions are activating or repressing as long as their strength is severely limited.

Interactions between overlapping gap domains are involved in regulating the dynamic anterior shifts of posterior expression boundaries over time. Jaeger *et al.* [46] suggested that the observed shifts are due to asymmetric repressive interactions, where the neighbouring domain at the posterior represses its adjacent domain at the anterior, but not *vice versa*. Therefore, repression of posterior by anterior neighbours (*gt* by Kni, and *hb* by Gt) poses a problem for this postulated mechanism. Still, our mRNA circuits exhibit correct shifting dynamics of posterior gap gene expression (Figure 5A). Graphical analysis of our mRNA-based models (Figure 6C) reveals the following relaxed condition for the shift mechanism: instead of complete absence of anterior-to-posterior repression it only requires an asymmetry in repressive strength. To be more precise, we define the net effect of an interaction as its regulatory weight multiplied by the concentration of the regulator integrated over the relevant region of the embryo (Figure 6C). Anterior shifts occur as long as the net effect of the anterior-to-posterior interaction is less repressive than the reciprocal posterior-to-anterior effect. This implies that the anterior-to-posterior interaction can be either activating or repressing, as long as it does not cancel out repression by the posterior neighbour. This allows the posterior repressor to become expressed in the posterior region of its anterior neighbour's domain, down-regulating the latter, and thus leading to the observed anterior shift of the latter's boundary.

In summary, none of the differences between mRNA- and protein-based circuits shown in Figure 6B are inconsistent with regulatory mechanisms for gap gene regulation postulated previously [46], [48]–[50] (see Box 1). The network recovered from both data sets is essentially equivalent. Our models—just as those obtained with protein data—predict that gap genes are regulated by (1) broad activation by maternal gradients, (2) auto-activation terms, which are non-essential, but serve to maintain sharply defined domain boundaries [50], (3) strong mutual repression between non-overlapping gap genes (*hb* and *kni, Kr* and *gt*; we call this mechanism ‘alternating cushions’ for reasons explained in [35]), (4) weak repression between overlapping gap domains showing posterior dominance which leads to dynamic anterior shifts in domain position, and (5) additional repression of gap genes at the posterior pole by terminal gap genes *tll* and *hkb* (Figure 6D; see [35] for review).

### Determinability of Gene Circuit Parameters

Sets of parameter estimates based on reduced-quality mRNA data show increased variability compared to protein-based circuits—both in terms of the distribution of parameter values (Figure 6A), and the regulatory categories they fall into (Figure 6B). Nevertheless, we have shown that we can recover consistent regulatory mechanisms from these estimates, if we consider the consensus network structure, that is, those regulatory categories into which a majority of the estimated parameter values fall into.

In this section, we examine if our parameter estimates are also determinable in the statistical sense defined by Ashyraliyev *et al.* [49], [64]. This is also known as practical parameter determinability analysis. It is achieved by assuming that our fitting problem has a single optimal solution, which we call the ‘true’ solution. We can then calculate confidence intervals around each one of our parameter estimates. These intervals determine a range of parameter values, which include—with a given probability of 95%—the true solution to the problem. A parameter is determinable, if its confidence interval lies entirely within one of our three regulatory categories: repression (parameter value less than *−0.005*), no interaction (between *−0.005* and *0.005*), or activation (greater than *0.005*). It is weakly determinable if its interval intersects two of these categories, but excludes the third. There are two different ways to calculate these confidence intervals: dependent intervals tend to underestimate the extent of the confidence region, while independent intervals have the tendency to overestimate it (see Materials and Methods for details). As in previous studies [49], [64], we use independent intervals to estimate the determinability of a parameter.

Under specific conditions, gap gene circuits fit to protein data can yield parameter estimates, which are very well determined (Table 3, top row). Specifically, estimates from WLS fits are much more determinable than those obtained by OLS, if parameter values for diffusion rates (*D*), threshold parameters (*h*), and certain regulatory weights (the effect of Hkb on *gt, Kr*, and *kni*) are fixed during optimisation [49]. In contrast, determinability of parameter estimates is poor in both OLS and WLS fits to mRNA data (Table 3, rows 5 and 6). This loss of determinability could be due to three reasons: (1) the use of mRNA instead of protein data; (2) our data processing method (and approximation of variances for WLS); and (3) the smaller embryo region covered by mRNA-based circuits.

**Table 3:** Parameter determinability in mRNA- and protein-based gene circuits.

Description of Scenario	Data	Scoring Function	Weights for WLS	Trunk Region (Nuclei)	Independent Confidence Intervals	Determinability
Gene circuits using protein data from [49]	protein	WLS	derived from protein data	58	20/5/4	good
Gene circuits using protein data and mRNA-style weights	protein	WLS	mRNA-style	58	16/6/7	reasonable
Gene circuits using protein data approximated by mRNA-style boundary extraction	protein	WLS	mRNA-style	58	11/3/15	reasonable
Gene circuits from mRNA and WLS, with 58 nuclei	mRNA	WLS	mRNA-style	58	0/3/26	poor
Gene circuits from mRNA and WLS	mRNA	WLS	mRNA-style	53	0/2/27	poor
Gene circuits from mRNA and OLS	mRNA	OLS	not used	53	0/1/28	poor

Each row represents the results of a series of optimisation runs to data described in columns 2–5: mRNA- or protein-based fits, OLS or WLS cost function, variance-based or approximated (mRNA-style) weights for WLS, and region covered by models (53 or 58 nuclei). Column 6 (‘Independent Confidence Intervals’) shows triplets, which represent the number of regulatory parameters in fitted models that are determinable/weakly determinable/non-determinable. Determinable parameters are those whose confidence intervals fall exclusively into one regulatory category (activating, no interaction, or repressing). Weakly determinable parameters are those where one regulatory category is excluded from the confidence interval (‘not repressing’, or ‘not activating’). Confidence intervals for regulatory weights in all scenarios are shown in Text S4. Overall determinability of parameters is summarised in column 7.

To distinguish between these three possibilities, we performed several series of optimisation runs. First, we used the original protein data set with approximated variances that only depend on expression level (as described for our mRNA data in Materials and Methods). This yielded a level of parameter determinability, which was somewhat worse, but still comparable to the original protein data set (Table 3, row 2). We then approximated expression boundaries in the integrated protein data set by our spline-based method, to emulate domain and boundary shapes equivalent to the mRNA-based data. Again, this yielded decreased but still reasonable parameter determinability (Table 3, row 3). Based on this, we conclude that neither the use of approximated weights, nor the use of approximated boundaries can account for all the loss of determinability observed in mRNA-based circuits. Finally, we performed fits to mRNA data on an extended region of 58 nuclei, equivalent to the region used for protein-based gene circuits. As for other mRNA-based solutions, determinability was poor, significantly decreased compared to any of the protein fits (Table 3, row 4). Taken together, the above evidence indicates that the most relevant factor for the loss of statistical determinability is the use of mRNA- instead of protein-based expression data.

### Reverse Engineering with Reduced Data Sets

**Figure 7:**
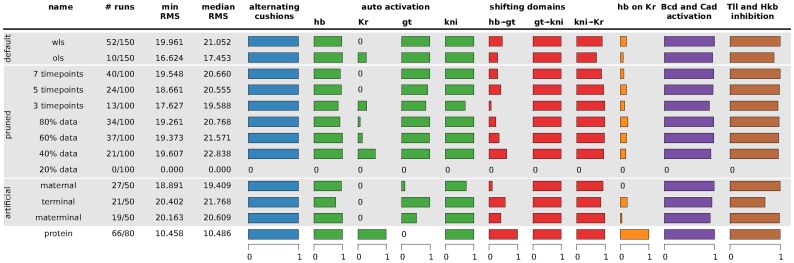
Summary of gene circuit performance under various scenarios of data availability. Scenario names are described in Table 4. Column 1–4: for each scenario, the number of selected/total runs is given; the minimum and median RMS score of the selected runs is indicated. The horizontal bar graphs to the right represent fractions of the selected runs in which specific regulatory mechanisms were found to be present. Smaller bars correspond to single interactions, larger to summaries of multiple interactions. Blue column: alternating cushions refers to the inhibiting interactions of *hb* and *kni*, as well as *gt* and *Kr*. Green columns: auto-activation is defined as a gene having an activating interaction with itself. Red columns: shifting domains are defined in terms of the following net effects between overlapping gap domains (see main text for the definition of net effects): posterior *hb* represses posterior *gt*, *gt* represses *kni*, and *kni* represses *Kr* (cf. Figure 6C). Orange column: the net repressive interaction between *hb* and *Kr*, as calculated for other overlapping gag domains above. Bars indicate net repression of *Kr* by Hb, which is the standard situation in protein-based circuits, while most mRNA-based circuits show net repression of *hb* by Kr. Purple column: Bcd and Cad activation is defined as the fraction of gene circuits in which Bcd activates all four gap genes and Cad activates *gt* and *kni*. Brown column: Tll and Hkb inhibition is defined as Hkb inhibiting *hb*, while Tll inhibits the other three gap genes. See text for details. The results summarised here are based on the interconnectivity matrices shown in Text S3.

In the previous sections, we have established that it is possible to reverse-engineer a developmental gene regulatory network from mRNA expression profiles. Even though these mRNA-based networks lack statistical determinability, the observation that we consistently recover a qualitatively equivalent network generates a certain confidence in the methodology. Next, we wanted to define the minimal requirements for gap gene circuits in terms of experimental data. As mentioned in the Introduction, the most time-consuming and hence limiting step involved in this method is the acquisition and processing of the quantitative experimental data required for model fitting. Thus, we would like to reduce and simplify our data as much as possible. To this end, we have performed a series of model fitting runs with reduced data sets (either by decreasing the number of boundaries, or the number of time classes), or with artificially generated data for external inputs (to simulate the case where quantitative protein data on maternal gradients and terminal gap genes would be unavailable). Table 4 presents an overview of the distinct data sets used for model fitting, while the analysis of the resulting gene circuits is summarised in Figure 7 (based on interconnectivity matrices shown in Text S3).

**Table 4:** Data sets used for model fitting.

Scenario name	Description
OLS	Default settings (full data set), using the ordinary least squares cost function.
WLS	Default settings (full data set), using the weighted least squares cost function.
80%, 60%, 40%, 20% data	Individual boundaries were eliminated randomly from the full data set such that only a fraction of 80% (or 60, 40, 20%) is used to calculate median boundary positions. For each identified boundary and time point, at least one individual data point is retained, to avoid artifactual fusion or ectopic extension of domains. For each fraction we generated 5 different data sets, on which we ran 20 optimisation runs each, resulting in a total of 100 runs. See Figure S4.
7, 5 and 3 time classes	Intermediate time classes were eliminated randomly such that only 7 (or 5, or 3) of a total of 9 time classes are present. The initial and final time classes are never removed from the data. Again, we generated 5 different data sets per time class reduction, on which we ran 20 optimisation runs each, resulting in a total of 100 runs. See Figure S5.
maternal	An artificial Bcd protein gradient is generated by fitting an exponential curve to expression data across all time points. The resulting profile is constant in time. The artificial Cad protein gradient is created by splines maintaining 3 salient features of the original pattern: (1) gradient in the central region of the embryo, constant high level of protein in the posterior, (2) abdominal expression levels decay over time, and (3) a posterior stripe is formed between T6 and T8. See Materials and Methods, and Figure S6.
terminal	Tll and Hkb protein expression data are replaced by time-invariant data based on integrating mRNA expression profiles (see Materials and Methods).
materminal	Combines the maternal and terminal data sets, such that all external inputs (Bcd, Cad, Tll and Hkb) are replaced by artificial variants as described.

This table lists reduced or artificial data sets which were tested and compared to fits to the full data set, and protein data (see Figure 7). Fits to full mRNA data were done using both OLS and WLS cost functions. Fits to reduced/artificial data sets were only performed with WLS. Names of data sets correspond to those used in Figure 7 and the main text.

Our results show that a smaller fraction of solutions obtained by fitting to reduced data sets are usable for analysis. This applies to both reduction of the number of boundaries and time points present in a data set (Figure 7, 2nd column). In contrast, the optimum and median RMS scores of these fits do not show any clear trend, although RMS scores slightly increase as boundaries are removed (Figure 7, 4th column). Using artificial external inputs did not significantly affect the number of usable solutions or the RMS score. We conclude that our approach is surprisingly robust to the reduction in quality of our data set. Only in one case—reduction of the number of boundaries to 20%—did we completely fail to get any solutions suitable for further analysis.

While a good fit to the data is important, it is even more crucial that gene circuits represent consistent gene network structures and regulatory mechanisms. For this reason, we examined whether models fit to reduced or artificial data incorporate the five basic mechanisms of gap gene expression described in the previous section.

First, we looked at mutual repression of non-overlapping gap genes (alternating cushions). This mechanism is recovered extremely robustly: it is present in all selected solutions (Figure 7, blue column). In agreement with previous analyses [50], we conclude that it lies at the core of gap gene regulation.

With respect to auto-activation, we observe that all mRNA-based circuits behaved similarly, with the exception of the gene circuits from fits with artificial maternal gradients (Table 4, and Figure 7, green columns). In general, *hb*, *gt* and *kni* were auto-activating, while *Kr* was not (or not consistently, at least). In addition fits with artificial maternal gradients show reduced presence of *gt* auto-activation. The variability in these results can be explained by the fact that auto-activation is not essential for positioning gap domains in gene circuit models [50].

To analyse the presence or absence of the domain shift mechanism [46], we examined net effects of repression between overlapping gap domains as described in the previous section (Figure 7, red columns; see also Figure 6C). We observed that two domain interactions were consistently present in our models: the net repression of *Kr* by Kni, and that of *kni* by Gt. However, net repression of *gt* by Hb was only recovered in about 10–50% of solutions. In those circuits that do not show this effect, Gt represses *hb* in the anterior to reproduce the peak of *hb* expression in the middle of the embryo. We have shown elsewhere that this is a modelling artefact [48]. Repression of *hb* in the posterior is overcome in these circuits by strong activation of *hb* by Tll (data not shown), an interaction which is likely to be indirect, and therefore not supported by experimental evidence [75].

Unlike the interactions described above, mutual repression between the overlapping domains of *hb* and *Kr* does not contribute to gap domain shifts [46]–[48]. If we consider the net effect of these reciprocal interactions, *hb* is repressed by Kr in the majority of cases (Figure 7, orange column). In contrast, most protein-based circuits show net repression of *Kr* by Hb. We explain this as follows: the border between *hb* and *Kr* is maintained at the same A–P position over time (Figure 4; see also [43], [46]). There are several regulatory mechanisms that can accomplish this. Protein-based circuits exhibit auto-activation on both sides plus a minimal net repressive effect of Hb on *Kr*, while mRNA-based circuits favour auto-activation of *hb* only, plus stabilisation of the *hb/Kr* border by a slight net repression of Kr on *hb* (Figure 7, green and orange columns). The experimental evidence on which of these alternative mechanisms applies in the embryo remains inconclusive: while the potential presence of *Kr* auto-activation [76] favours the first, the absence of any defect in the posterior *hb* boundary in *Kr* mutants supports the second mechanism [77], [78].

Next, we looked at activation of gap genes by the maternal factors Bcd and Cad. This mechanism is recovered very robustly (Figure 7, purple column), in agreement with previous studies [46], [48]–[50]. Note that the summary bar graphs of Figure 7 (purple column) omit the effect of Cad on *hb* and *Kr*, as these interactions are an artifactual aspect of the model ([48], and references therein).

Finally, the terminal gap genes Tll and Hkb repress gap genes at the posterior pole of the embryo. In general, we recover this mechanism robustly (Figure 7, brown column). However, if Tll and Hkb protein gradients are substituted by mRNA profiles (see Table 4), about 20% of the solutions fail to show repression of Tll on *gt*. In these circuits, repression of *gt* by Hb is sufficient for the retraction of its posterior domain from the pole (data not shown). Interestingly, recovery of this interaction is rescued in those fits in which both maternal and terminal external inputs were replaced by ‘artificial’ patterns (see Figure 7).

In summary, we have managed to recover surprisingly correct and accurate gap gene regulatory mechanisms even with data sets of severely reduced coverage and/or quality. This does not imply that we can reconstitute specific gene networks with arbitrary data. On the contrary, successful network inference requires very specific conditions for the data used in model fitting. We will revisit this important point in the Discussion.

## Discussion

Earlier studies using reverse-engineering approaches were based on quantitative protein expression data [46], [48]–[50]. These protein data capture the precise shape of each expression domain, as well as differences and changes in relative protein levels both between domains of the same gene, and specific gap domains over time. In this paper, we have demonstrated that we can correctly infer the regulatory structure and dynamics of the gap gene network in *Drosophila melanogaster* using spatial mRNA-based expression data of reduced quality and coverage. Our mRNA data only capture the dynamics of boundary positions, and even that at a reduced spatial resolution. Furthermore, our simplified data processing pipeline leads to a loss of information on relative expression levels, which need to be approximated based on straightforward biological assumptions on transcriptional regulation and gene expression (gradual increase and decrease of levels over time).

On the other hand, this type of mRNA data can be acquired and processed within a fraction of the time and effort it takes to obtain high-quality protein-based expression patterns: instead of years, it now only takes a few months to establish quantitative expression profiles for a complete set of genes involved in a developmental process. Our approach avoids having to raise antibodies against regulators, which is both technically challenging and expensive. It uses a robust colorimetric (enzymatic) staining protocol instead of fluorescence. It avoids laborious scanning of embryo images using confocal microscopy (which is not available everywhere). In addition, we show that much fewer embryos need to be processed and stained, and fewer developmental stages need to be represented in the data than previously thought. And finally, our approach simplifies data processing, further reducing the effort required for data quantification.

### Minimal Data Requirements for Reverse Engineering

Does the fact that we still recover the correct gap gene network using mRNA data imply that our method lacks specificity? Would it infer the same network with any kind of data? The following evidence demonstrates that the answer to these questions is a clear no, and suggests minimal conditions for the expression data that must be met for inference to be specific and consistent.

First of all, gap gene circuit models fail to correctly predict gap gene expression in the head region of the embryo (anterior of 35% A–P position) [45], [48]. In this region, additional regulators (the head gap genes which are not included in our models; see [35] for review) are required for correct regulation and expression. This implies that the model-fitting procedure is specific: it fails when relevant regulatory factors or mechanisms are missing.

In contrast, we have mentioned earlier that accurate measurements of absolute expression levels do not seem to be crucial for correct network inference (see Introduction). Our results suggest that we can recover the structure of the gap gene network even if relative levels are only approximated in the data. This confirms that the most important expression feature for network inference is the dynamic positioning of expression boundaries.

This is further corroborated by the following: early attempts at reverse-engineering the gap gene system using gene circuits exhibited various patterning defects and showed inconsistent network structures between independent model fits [45]. In contrast, we are able to infer correct network structure using data sets with spatial and temporal resolution similar to that used in [45] (Figure 7). Moreover, our data sets approximate gap protein domain positions with mRNA data (Figure 4). This suggests that the failure of inference in the earlier study was not caused by a lack of accuracy or resolution, but rather by qualitative errors regarding the relative placement of domains in the data used for model fitting. As mentioned in the Introduction, the abdominal *kni* domain was placed too far anterior in those data, leading to an exaggerated overlap with the central *Kr* domain, and an artefactual gap between the domains of *kni* and *hb* in the posterior of the embryo (see Figure 1 in [45]). Similar problems affected an early study of the *eve* gene: in the data used for fitting, the position of the fifth expression stripe was shifted compared to the domains of its gap gene regulators, which led to inaccurate prediction of the regulatory mechanism underlying its expression [39].

What differs between these early attempts and more recent reverse-engineering studies is that both protein- [43] and mRNA-based data used in the latter capture the relative arrangement and timing of gap domains correctly. Both data sets show qualitatively equivalent expression patterns (see Results, Figures 3 and 4). The order in which gap domains are arranged along the A–P axis, as well as the order in which they appear relative to one another are the same for mRNA and protein data. Evidently, this temporal and spatial order is determined by the specific regulatory structure of the network (given suitable kinetic parameters). This, in turn, allows us to robustly recover the specific regulatory structure of the gap gene network as long as our data capture their particular kind of spatio-temporal dynamics.

Obviously, there are limits to the amount of inaccuracy the data can contain with regard to absolute positions and variability of the patterns. This is shown by the fact that we were unable to recover any usable gap gene circuits with data sets reduced to 20% of the original number of measured domain boundaries (Figure 7). These data sets only contain about 1–3 embryos per boundary per time class, while the smallest successful data set (40% data) has about 1–7 embryos on average.

In general, the number of embryos that need to be processed will depend on the natural variability of the patterns and the quality of the experimental protocols: noisy expression data require larger sample sizes. Similarly, temporal resolution will depend on the time scale of patterning dynamics. In our case, only 3 time classes (each about 25 min apart) were sufficient to recover a correct regulatory network. This is probably due to the fact that gap domains shift and develop smoothly over time (see Figure 4). Systems with more abrupt or uneven changes in expression patterns will require a higher temporal resolution.

Our investigation of minimal data requirements for model fitting is of an empirical nature. It should be corroborated and extended in the future by more systematic and rigorous approaches based on methods for optimal experimental design (OED, reviewed in [79]). OED uses algorithms for global optimisation and calculation of confidence intervals similar to those used here to predict which measurements in a data set are most relevant to accurately estimate parameter values (see, for example, [80], [81]). This could be used for a more rational design of data sets used for reverse-engineering by guiding the choice of observables and time points that are most informative to infer network structure and dynamics. However, applying OED to real-world, complex, non-linear systems remains challenging, and has only be achieved in exceptional cases (see [82]). Therefore, it is beyond the aim and scope of our current study.

In summary, our results demonstrate that the amount of data required for reverse engineering is much lower than previously thought. The necessary data sets can be acquired and processed by a single researcher within a few months, without the need for expensive equipment. The precise number of embryos to be processed, and the required temporal resolution need to be adapted according to the features of the system under study, the main condition being that the data capture the relative timing and spatial arrangement of expression domains correctly.

### Weighted Least Squares and Artificial Variances

The results reported in this study—together with earlier evidence from protein-based circuits [49]—indicate that weighted least squares (WLS) fits perform much better than ordinary least squares (OLS). In principle, however, WLS fits require the accurate measurement of variances in expression levels to calculate the weights for the sum of squares. This is not possible in our current data quantification framework. Moreover, in general it increases the amount of work required for data acquisition and processing considerably. First, methods that measure the relative level of expression accurately are technically more challenging and require more work than those presented here (see previous section). And second, enough individual expression patterns need to be quantified for the measured variance to be reliable.

We avoid this complication by using approximated weights for WLS, which are simply proportional to the expression level of a gene. This assumption is mainly based on methodological reasoning (although it is also supported by experimental evidence on protein expression patterns [40], [43]). It is crucial to avoid small ectopic expression domains in non-expressing regions that can exert significant regulatory effects if interaction weights are sufficiently large. Several such ectopic domains have been observed in fits using OLS (our data and [47], [49], [51]). WLS fits effectively suppress these modelling artefacts, as long as the penalty for ectopic expression imposed by small variances in non-expressing regions are sufficiently high. This suggests that our choice of approximated variances is justified for practical reasons, since it emphasises the importance of boundary positions during the fitting process.

### Variability of Predictions: mRNA vs. Protein

Although we do recover the same network from our mRNA-based models as that predicted by protein-based circuits, there is much increased variability in the estimated parameter values (Figure 6A,B). This is also reflected in the loss of parameter determinability we observe in our results (Table 3). In practice, both of these phenomena imply that our mRNA-based models do not predict one, but rather a small set of possible network structures, while protein-based models predicted a specific, single network (see Figure 6B). This is not surprising, since the quality of a gene circuit model reflects the quality of the data it was fit to. But of course it is a problem, although, we would argue, not a fundamental one. It can easily be addressed by combining the reverse-engineering approach with experimental (genetic and molecular) verification.

Let us illustrate this with an example: there are several alternative network variants that occur in our circuits. They are all minor in that they differ from each other in one or two interactions at most. One of these variants occurs in models in which the posterior domain of *hb* arises due to strong and direct activation by Tll, rather than the absence of repression by Gt, its immediate anterior neighbour (see Results). This alternative mechanism is a plausible explanation for the observed expression dynamics. However, it is not compatible with experimental evidence, which enabled us to classify it as an artefact of the model.

Note that both the experimental evidence (reviewed in [35]) and predictions based on gene circuits [48], [49] contain such ambiguities. Fortunately, these unresolved (and potentially not resolvable) cases have only a limited overlap, since genetic and reverse-engineering approaches are complementary to each other: one inferring regulatory interactions from mutants, the other from wild-type expression patterns [48]. In other words, interactions which are not clearly supported by experimental evidence are mostly unambiguous in our gene circuits, while others that are ambiguous in our models (as, for example, the repression of *hb* by Gt), are clearly supported (or excluded) based on experimental evidence.

But what about systems which have been less well studied than *Drosophila*? In such systems, we do not have a comprehensive experimental literature to compare our results to. Instead, model predictions will have to be tested using genetic approaches such as mutant analysis, over-expression assays, or gene knock-down by RNA interference (RNAi). Again, the reverse-engineering method is most powerful when used in conjunction with complementary experimental approaches.

### Limitations and Future Potential of the Method

By minimising the amount of quantitative data required for reverse-engineering a developmental gene regulatory network, we have removed a major bottleneck for applying the method more widely. Still, this method is unlikely to be scalable to systems that are orders of magnitude larger than the one studied here. Microscopy and image acquisition remain labour-intensive, and our quantification pipeline still requires a series of manual interventions, such as positioning the splines that are fit to expression boundaries, or time classification of embryos. It remains a major challenge to fully automate these steps. Therefore, the effort required to quantify hundreds or thousands of spatial gene expression patterns is still considerable, even if robust and fast methods are used (see, for example, [83], [84]). Moreover, global non-linear optimisation is computationally intensive, and may not yield unique solutions in large regulatory systems.

On the other hand, many pattern-forming networks are similar in complexity and nature to the gap gene system. One example in *Drosophila* is the dorso-ventral patterning system in the early embryo [85]–[87]. Other developmental networks occur in the context of cellularised tissues and often involve more than just one spatial dimension. It is straightforward to extend the gene circuit method to such systems. Complicating factors, such as post-transcriptional regulation, cell-to-cell signalling, or tissue movements and growth, can readily be accommodated in the gene circuit modelling formalism [38]. There are several examples of tractable cellular patterning systems in *Drosophila*: mesoderm and heart development [88], [89], morphogen-based patterning in wing imaginal discs [87], [90], or the thoracic bristle patterning system [91], [92]. Examples beyond *Drosophila* include vulval induction in the roundworm *Caenorhabditis elegans* (see, for example, [93]), or the dorso-ventral patterning system of the vertebrate neural tube [94].

Generalisation of the method is further facilitated by the fact that the approximations we have used are based on straightforward assumptions that do not require any in-depth understanding of the system under study. Our approximation of relative gene expression levels assumes smooth increase and decrease of gene product levels. The scaling of expression levels from the centre to the terminal ends of the embryo is a reasonable assumption in the case of *Drosophila*, but may be omitted in any other system as it is not strictly required for inferring the correct network structure. Approximated variances simply assume low levels of variability in non-expressing regions. Artificial external inputs were based on qualitative inspection of maternal gradients and expression of terminal gap genes. All of these assumptions can be applied to systems other than the gap gene network. The only potentially problematic assumption concerns the use of mRNA expression profiles. Obviously, one should have some qualitative evidence that mRNA patterns indeed resemble protein profiles before relying on such data.

Application of reverse engineering to a large number of developmental systems in different organisms would allow us to investigate the dynamics of pattern-forming gene networks in a quantitative and systematic manner [26]. Our simplification of the method indicates that this is achievable within a reasonable amount of effort. The potential benefits of such a research programme are significant. Only through quantitative investigation of specific, experimentally accessible, gene networks will we be able to better understand the principles that govern development and pattern formation.

A particularly interesting application of the gene circuit method is the comparative analysis of homologous gene networks across different species [26]. Such a comparative analysis allows us to identify conserved and divergent regulatory interactions in an evolving network. Moreover, changes in regulatory mechanisms can be mapped—rigorously and unambiguously—to observable differences in gene expression between species. We are currently performing such an analysis between gap genes in *Drosophila* and other dipteran species. It will allow us, for the first time, to study the evolution of a real developmental gene regulatory network in a detailed and quantitative manner. This is a crucial step towards a more general investigation into the causal relation between evolution at the genotypic and the phenotypic level.

## Supporting Information

Figure S1 **Post-processing steps.** (A) Our data quantification method results in normalised mRNA expression data. All boundaries are approximated by splines, and all domains have the same standard expression level. (B) Relative mRNA concentrations are scaled along the A-P axis to reflect higher expression levels in the middle of the embryo. (C) Relative concentrations are also scaled over time, taking the basic biological assumption that mRNA levels are low at the start of C13, peak at C14-T5 and have diminished by C14-T7. (D) To easily compare the protein and mRNA gene circuits concentration levels are scaled by a factor 200. Horizontal axes represent A–P position, where 0% is the anterior pole. Relative mRNA concentrations are in arbitrary units (au). C13 is cleavage cycle 13, T3/7 represent time classes during C14A. Time progresses downwards. Our models only include the trunk region of the embryo, ranging from 35% to 87% (grey background in D).
(PDF)

Figure S2 **Model fitting solutions selected for further analysis.** Histograms show root-mean-square (RMS) scores for solutions obtained using OLS (A) and WLS (B) cost functions. White bars constitute the histogram of 150 total runs performed in each setting. Blue bars indicate solutions selected for further analysis (based on visual inspection): 10 for OLS and 52 for WLS. Note that selection of sub-optimal runs in (A) indicates over-fitting of the data. This effect is greatly reduced in (B). See main text for details.
(PDF)

Figure S3 **OLS mRNA fits: model output versus data.** Plots show model output (solid lines) versus expression data (dashed lines). All 10 selected gene circuits fit with OLS to mRNA data are shown. Horizontal axes represent AP position, where 0% is the anterior pole. Relative mRNA concentrations are in arbitrary units (au). T1/3/5/7 represent time classes during C14A. Time progresses downwards.
(PDF)

Figure S4 **Data sets based on reduced numbers of boundaries.** The first column shows the full data set at three time classes (T1/4/7) during C14A. This reference data set is also represented by dashed lines in the middle and right column to facilitate comparison. The two other columns show reduced data sets based on 60% (middle) and 20% (right) of randomly selected individual boundaries (at least one data point was always retained for each boundary position). Increasing deviations from the full data set can be observed as the number of boundaries is reduced. Horizontal axes represent A–P position, where 0% is the anterior pole. Only the trunk region of the embryo included in our models is shown. Relative mRNA concentrations are in arbitrary units (au). Time progresses downwards.
(PDF)

Figure S5 **Examples of mRNA gene expression data sets with reduced numbers of time classes.** The first column shows the full data set, followed by three columns of reduced data. We randomly select time classes in the range T1–T7 for elimination, which maintains the starting (C13) and end point (T8) of the time series. Random se- lection of time classes was performed 5 times, resulting in 5×20 optimisation runs for each category. Horizontal axes represent A–P position, where 0% is the anterior pole. Only the trunk region of the embryo included in our models is shown. Relative mRNA concentrations are in arbitrary units (au). See Materials and Methods for definition of time classes. Time progresses downwards.
(PDF)

Figure S6 **Measured and artificial maternal inputs.** Plots show a comparison of protein-based expression (data) and approximated, artificial expression profiles (art) for the maternal gradients of Bcd and Cad. (A) Quantitative Bcd data (solid) was approximated using a (time-invariant) exponential function (dashed line). (B) Cad data (solid) was approximated using splines (dashed line; see Materials and Methods for details). Horizontal axes represent A–P position, where 0% is the anterior pole. Only the trunk region of the embryo included in our models is shown. Relative protein concentrations are in arbitrary units (au). T1/4/7 represent time classes during C14A. Time progresses downwards.
(PDF)

Table S1 ***Drosophila*** **mRNA vs. protein expression: boundary shifts.** This table shows shifts in boundary positions from a starting point at T1, or, from the earliest appearance of the boundary domain. Numbers are in % A–P position. mRNA expression is shown in black, protein expression in grey. (−) indicates a shift to the anterior (+) a shift to the posterior. *Drosophila* values are calculated from [43]. Single dashes indicate boundaries that are not present at a given time point. Double dashes indicate boundaries that are observable, but were not measured in [43].
(PDF)

Table S2 ***Drosophila*** **mRNA versus protein expression: domain width.** This table shows domain widths for trunk gap genes. Numbers are given in % egg length. mRNA expression is shown in black, protein expression in grey. *Drosophila* values are calculated from [43]. Single dashes indicate boundaries that are not present at a given time point. Double dashes indicate boundaries that are observable, but were not measured in [43].
(PDF)

Text S1 **Common patterning defects in Gap Gene Circuits.**
(PDF)

Text S2 ***Drosophila*** **Segmentation Gene Expression.**
(PDF)

Text S3 **Genetic Interconnectivity Matrices.**
(PDF)

Text S4 **Dependent and independent parameter confidence intervals.**
(PDF)
