# Supplementary material for: Efficient Reverse-Engineering of a Developmental Gene Regulatory Network
Source: PLoS Comput Biol. 2012 Jul 12;8(7):e1002589. doi: 10.1371/journal.pcbi.1002589 (PMC3395622; doi:10.1371/journal.pcbi.1002589)
Supplement: Figure S1 — Post-processing steps. (A) Our data quantification method results in normalised mRNA expression data. All boundaries are approximated by splines, and all domains have the same standard expression level. (B) Relative mRNA concentrations are scaled along the A-P axis to reflect higher expression levels in the middle of the embryo. (C) Relative concentrations are also scaled over time, taking the basic biological assumption that mRNA levels are low at the start of C13, peak at C14-T5 and have diminished by C14-T7. (D) To easily compare the protein and mRNA gene circuits concentration levels are scaled by a factor 200. Horizontal axes represent A–P position, where 0% is the anterior pole. Relative mRNA concentrations are in arbitrary units (au). C13 is cleavage cycle 13, T3/7 represent time classes during C14A. Time progresses downwards. Our models only include the trunk region of the embryo, ranging from 35% to 87% (grey background in D). (PDF) [file pcbi.1002589.s001.pdf]

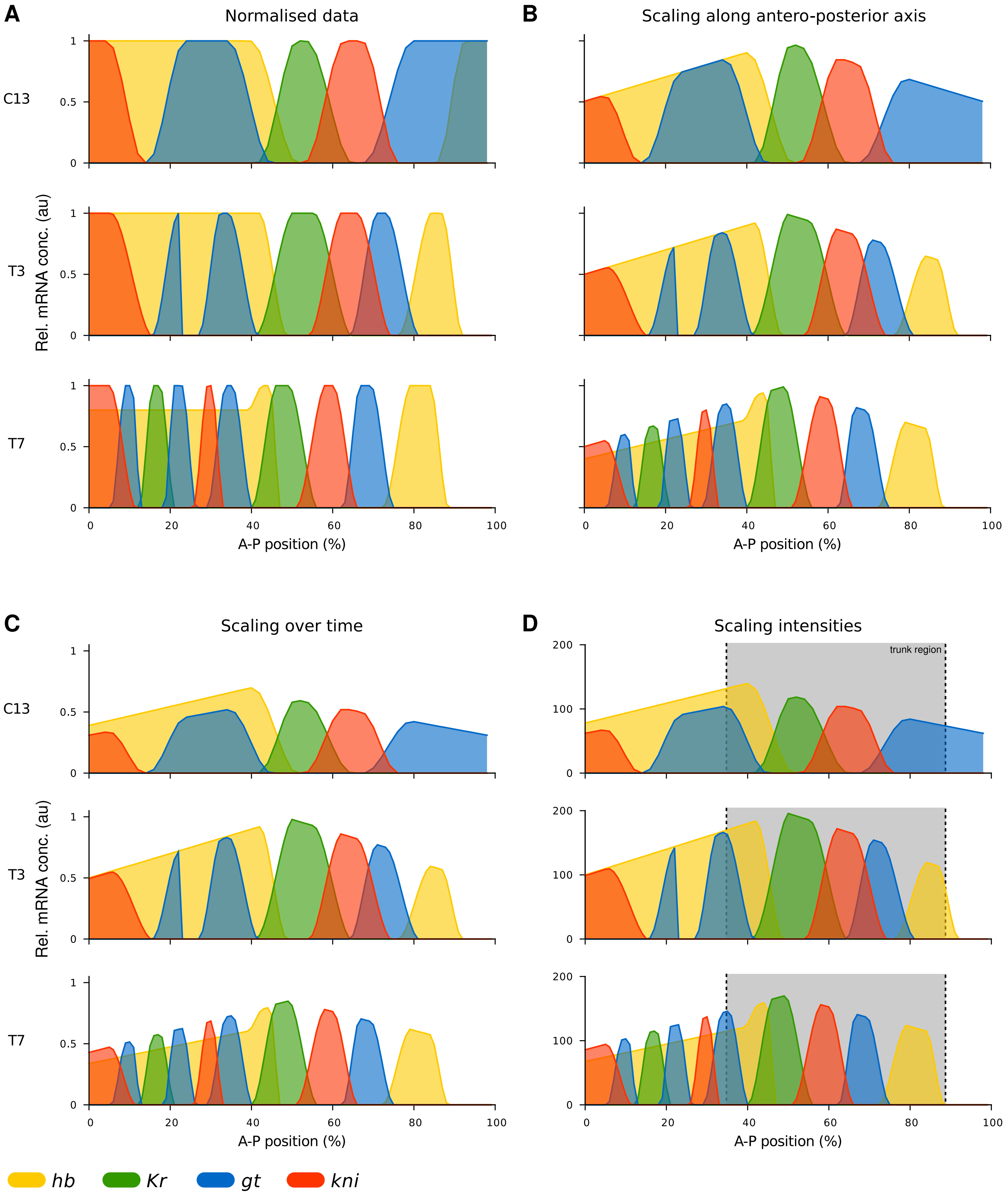

Figure S1. Post-processing steps. (A) Our data quantification method results in normalised mRNA expression data. All boundaries are approximated by splines, and all domains have the same standard expression level. (B) Relative mRNA concentrations are scaled along the A-P axis to reflect higher expression levels in the middle of the embryo. (C) Relative concentrations are also scaled over time, taking the basic biological assumption that mRNA levels are low at the start of C13, peak at C14-T5 and have diminished by C14-T7. (D) To easily compare the protein and mRNA gene circuits concentration levels are scaled by a factor 200. Horizontal axes represent A–P position, where 0% is the anterior pole. Relative mRNA concentrations are in arbitrary units (au). C13 is cleavage cycle 13, T3/7 represent time classes during C14A. Time progresses downwards. Our models only include the trunk region of the embryo, ranging from 35% to 87% (grey background in D).
