# Supplementary material for: Efficient Reverse-Engineering of a Developmental Gene Regulatory Network
Source: PLoS Comput Biol. 2012 Jul 12;8(7):e1002589. doi: 10.1371/journal.pcbi.1002589 (PMC3395622; doi:10.1371/journal.pcbi.1002589)
Supplement: Figure S2 — Model fitting solutions selected for further analysis. Histograms show root-mean-square (RMS) scores for solutions obtained using OLS (A) and WLS (B) cost functions. White bars constitute the histogram of 150 total runs performed in each setting. Blue bars indicate solutions selected for further analysis (based on visual inspection): 10 for OLS and 52 for WLS. Note that selection of sub-optimal runs in (A) indicates over-fitting of the data. This effect is greatly reduced in (B). See main text for details. (PDF) [file pcbi.1002589.s002.pdf]

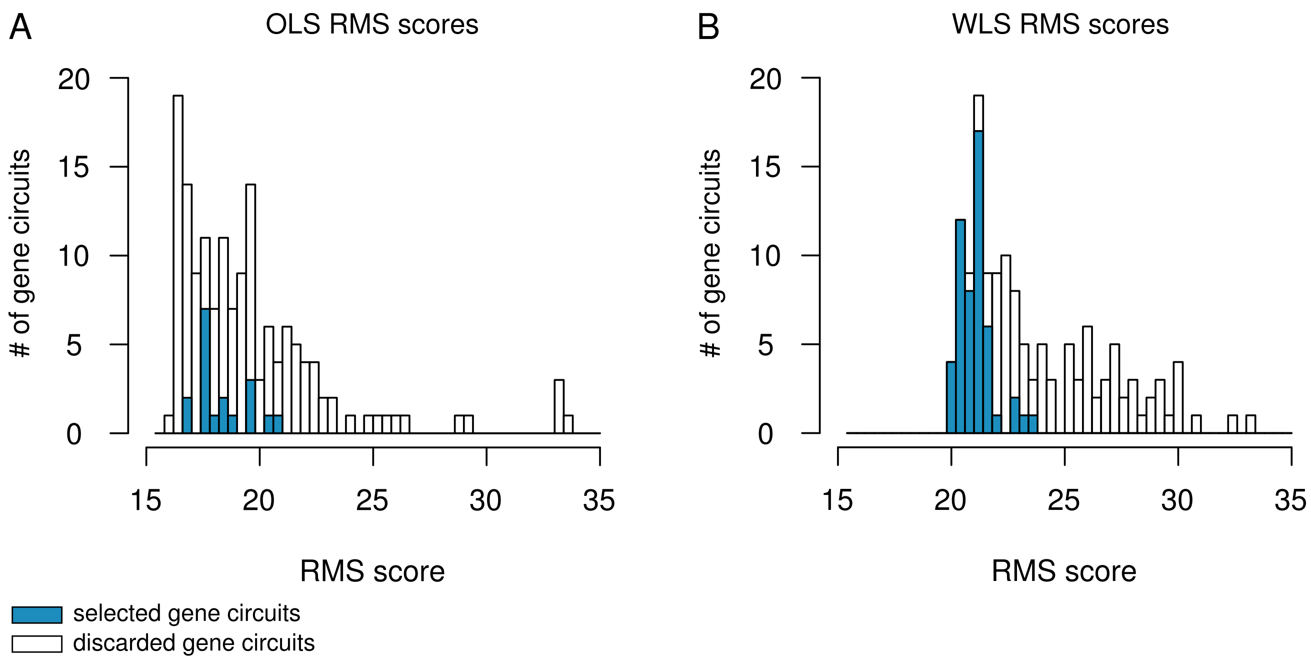

Figure S2. Model fitting solutions selected for further analysis. Histograms show root-mean-square (RMS) scores for solutions obtained using OLS (A) and WLS (B) cost functions. White bars constitute the histogram of 150 total runs performed in each setting. Blue bars indicate solutions selected for further analysis (based on visual inspection): 10 for OLS and 52 for WLS. Note that selection of sub-optimal runs in (A) indicates over-fitting of the data. This effect is greatly reduced in (B). See main text for details.
