# Supplementary material for: Efficient Reverse-Engineering of a Developmental Gene Regulatory Network
Source: PLoS Comput Biol. 2012 Jul 12;8(7):e1002589. doi: 10.1371/journal.pcbi.1002589 (PMC3395622; doi:10.1371/journal.pcbi.1002589)
Supplement: Figure S3 — OLS mRNA fits: model output versus data. Plots show model output (solid lines) versus expression data (dashed lines). All 10 selected gene circuits fit with OLS to mRNA data are shown. Horizontal axes represent AP position, where 0% is the anterior pole. Relative mRNA concentrations are in arbitrary units (au). T1/3/5/7 represent time classes during C14A. Time progresses downwards. (PDF) [file pcbi.1002589.s003.pdf]

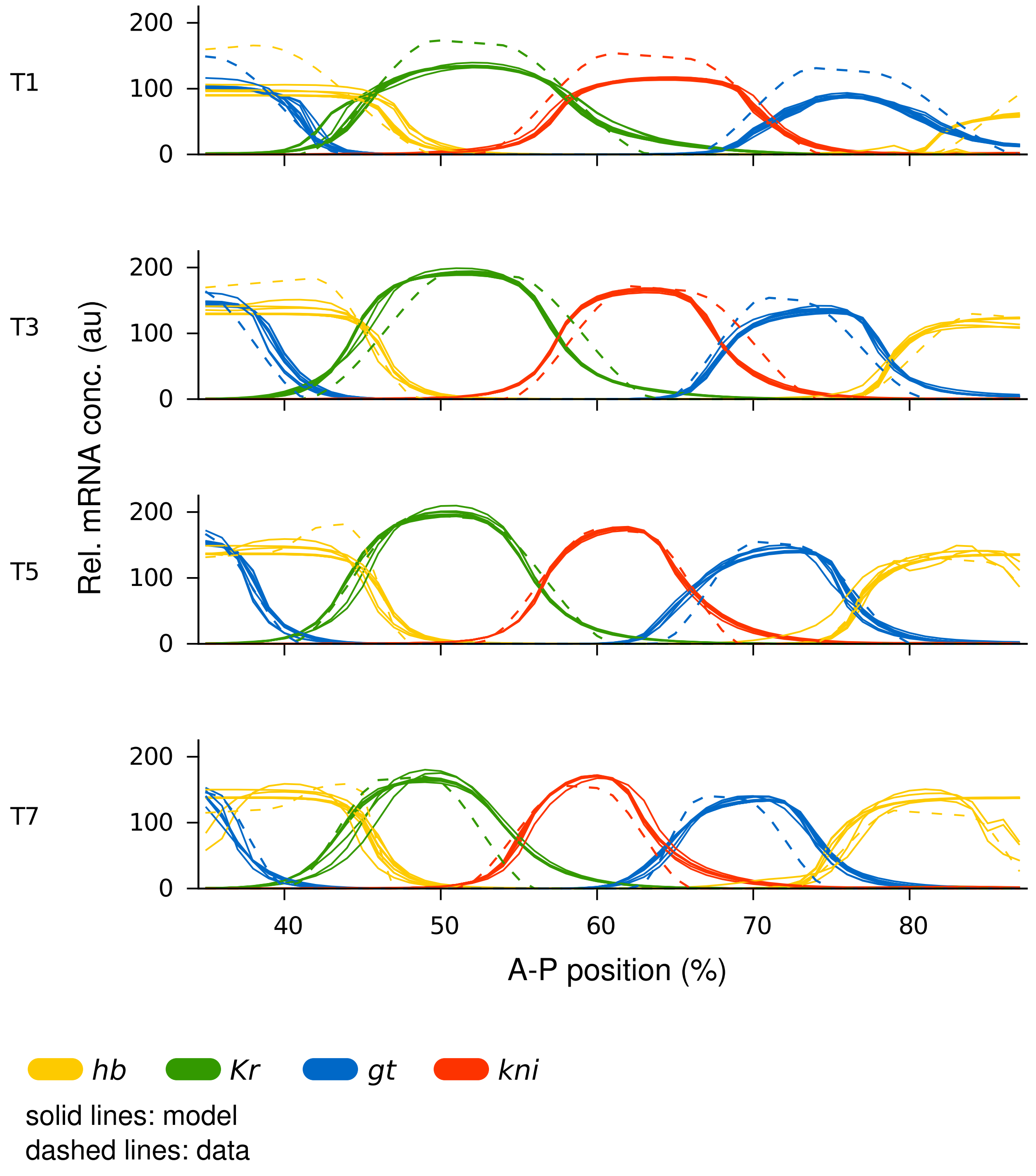

Figure S3. OLS mRNA fits: model output versus data. Plots show model output (solid lines) versus expression data (dashed lines). All 10 selected gene circuits fit with OLS to mRNA data are shown. Horizontal axes represent AP position, where 0% is the anterior pole. Relative mRNA concentrations are in arbitrary units (au). T1/3/5/7 represent time classes during C14A. Time progresses downwards.
