# Supplementary material for: Efficient Reverse-Engineering of a Developmental Gene Regulatory Network
Source: PLoS Comput Biol. 2012 Jul 12;8(7):e1002589. doi: 10.1371/journal.pcbi.1002589 (PMC3395622; doi:10.1371/journal.pcbi.1002589)
Supplement: Figure S4 — Data sets based on reduced numbers of boundaries. The first column shows the full data set at three time classes (T1/4/7) during C14A. This reference data set is also represented by dashed lines in the middle and right column to facilitate comparison. The two other columns show reduced data sets based on 60% (middle) and 20% (right) of randomly selected individual boundaries (at least one data point was always retained for each boundary position). Increasing deviations from the full data set can be observed as the number of boundaries is reduced. Horizontal axes represent A–P position, where 0% is the anterior pole. Only the trunk region of the embryo included in our models is shown. Relative mRNA concentrations are in arbitrary units (au). Time progresses downwards. (PDF) [file pcbi.1002589.s004.pdf]

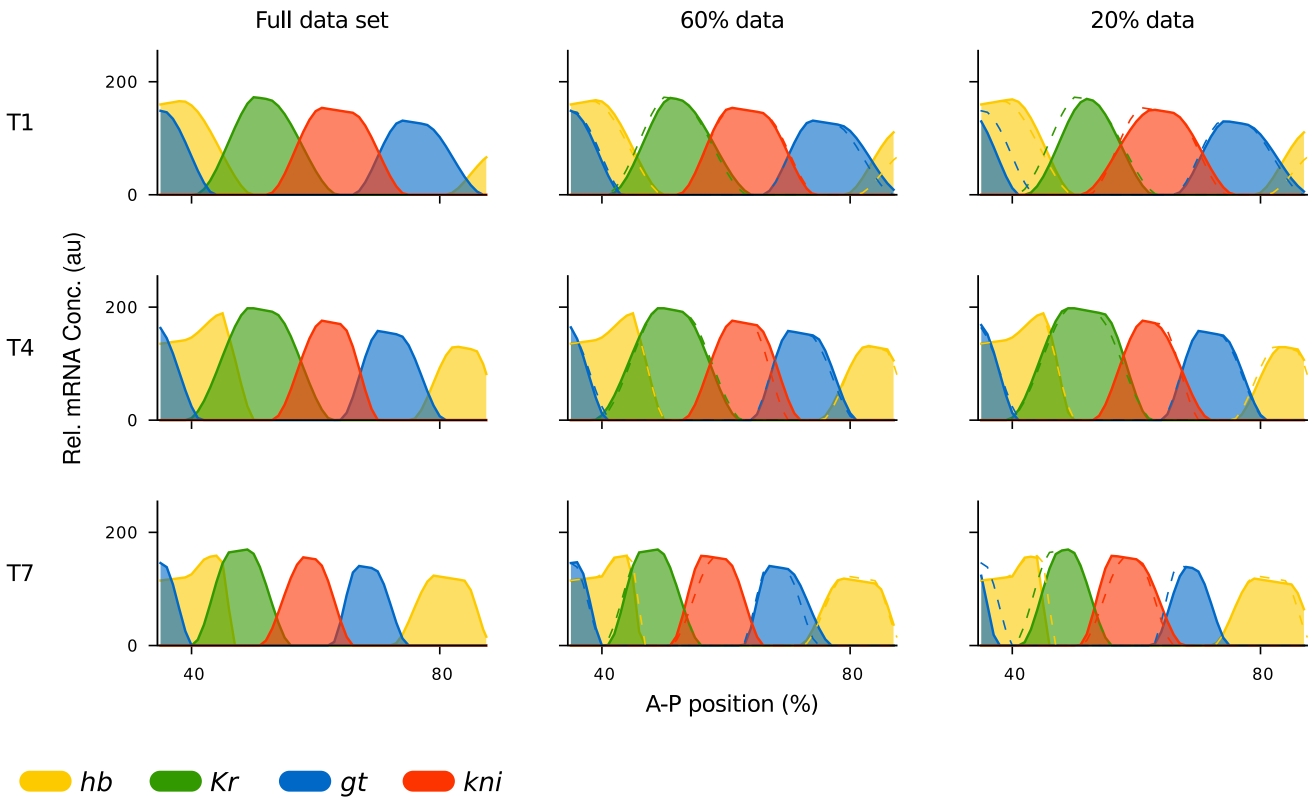

Figure S4. Data sets based on reduced numbers of boundaries. The first column shows the full data set at three time classes (T1/4/7) during C14A. This reference data set is also represented by dashed lines in the middle and right column to facilitate comparison. The two other columns show reduced data sets based on 60% (middle) and 20% (right) of randomly selected individual boundaries (at least one data point was always retained for each boundary position). Increasing deviations from the full data set can be observed as the number of boundaries is reduced. Horizontal axes represent A–P position, where 0% is the anterior pole. Only the trunk region of the embryo included in our models is shown. Relative mRNA concentrations are in arbitrary units (au). Time progresses downwards.
