# Supplementary material for: Efficient Reverse-Engineering of a Developmental Gene Regulatory Network
Source: PLoS Comput Biol. 2012 Jul 12;8(7):e1002589. doi: 10.1371/journal.pcbi.1002589 (PMC3395622; doi:10.1371/journal.pcbi.1002589)
Supplement: Figure S5 — Examples of mRNA gene expression data sets with reduced numbers of time classes. The first column shows the full data set, followed by three columns of reduced data. We randomly select time classes in the range T1–T7 for elimination, which maintains the starting (C13) and end point (T8) of the time series. Random se- lection of time classes was performed 5 times, resulting in 5×20 optimisation runs for each category. Horizontal axes represent A–P position, where 0% is the anterior pole. Only the trunk region of the embryo included in our models is shown. Relative mRNA concentrations are in arbitrary units (au). See Materials and Methods for definition of time classes. Time progresses downwards. (PDF) [file pcbi.1002589.s005.pdf]

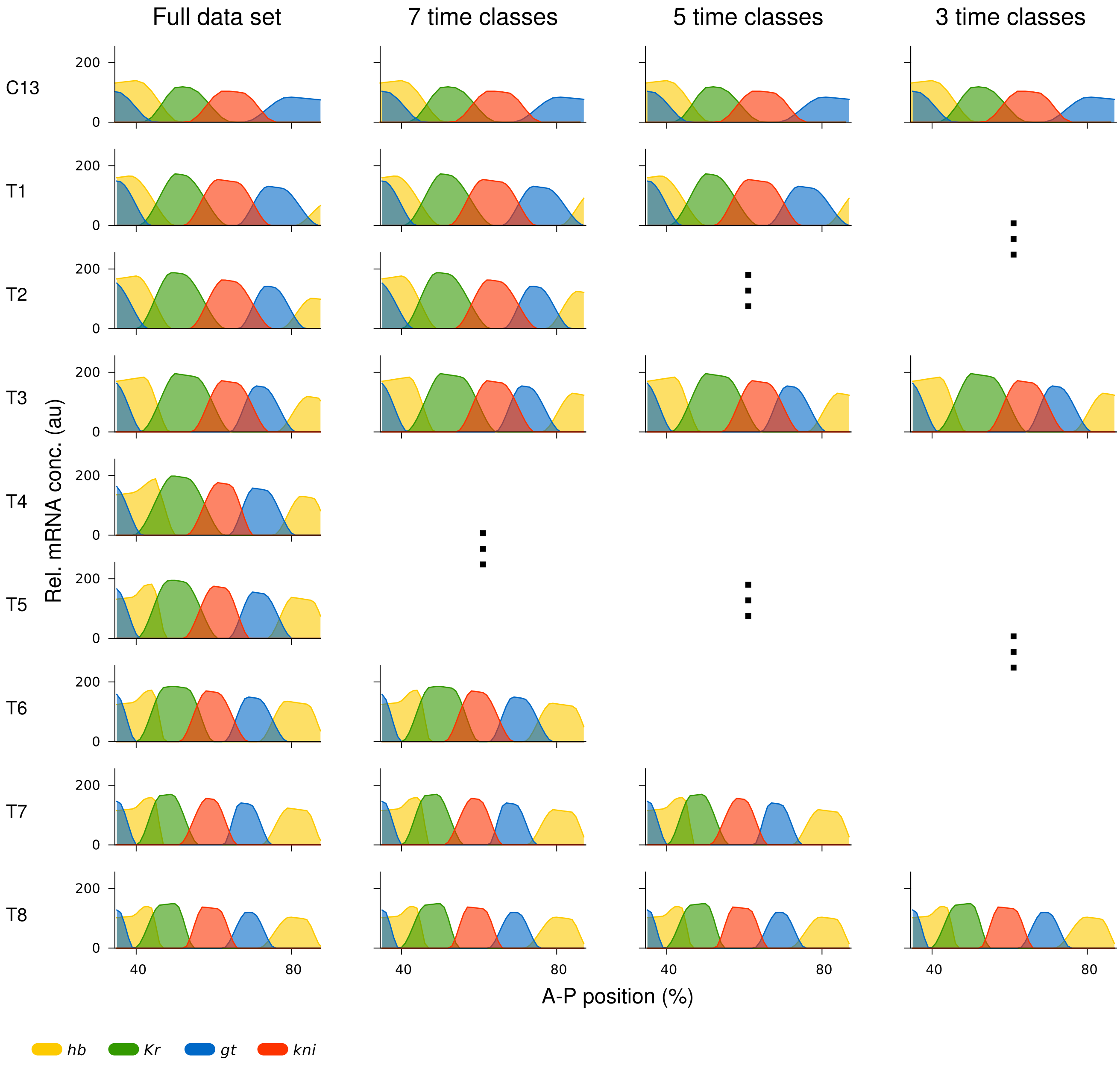

Figure S5. Examples of mRNA gene expression data sets with reduced numbers of time classes. The first column shows the full data set, followed by three columns of reduced data. We randomly select time classes in the range T1–T7 for elimination, which maintains the starting (C13) and end point (T8) of the time series. Random selection of time classes was performed 5 times, resulting in 5 $\times$ 20 optimisation runs for each category. Horizontal axes represent A–P position, where 0% is the anterior pole. Only the trunk region of the embryo included in our models is shown. Relative mRNA concentrations are in arbitrary units (au). See Materials and Methods for definition of time classes. Time progresses downwards.
