# Supplementary material for: Efficient Reverse-Engineering of a Developmental Gene Regulatory Network
Source: PLoS Comput Biol. 2012 Jul 12;8(7):e1002589. doi: 10.1371/journal.pcbi.1002589 (PMC3395622; doi:10.1371/journal.pcbi.1002589)
Supplement: Figure S6 — Measured and artificial maternal inputs. Plots show a comparison of protein-based expression (data) and approximated, artificial expression profiles (art) for the maternal gradients of Bcd and Cad. (A) Quantitative Bcd data (solid) was approximated using a (time-invariant) exponential function (dashed line). (B) Cad data (solid) was approximated using splines (dashed line; see Materials and Methods for details). Horizontal axes represent A–P position, where 0% is the anterior pole. Only the trunk region of the embryo included in our models is shown. Relative protein concentrations are in arbitrary units (au). T1/4/7 represent time classes during C14A. Time progresses downwards. (PDF) [file pcbi.1002589.s006.pdf]

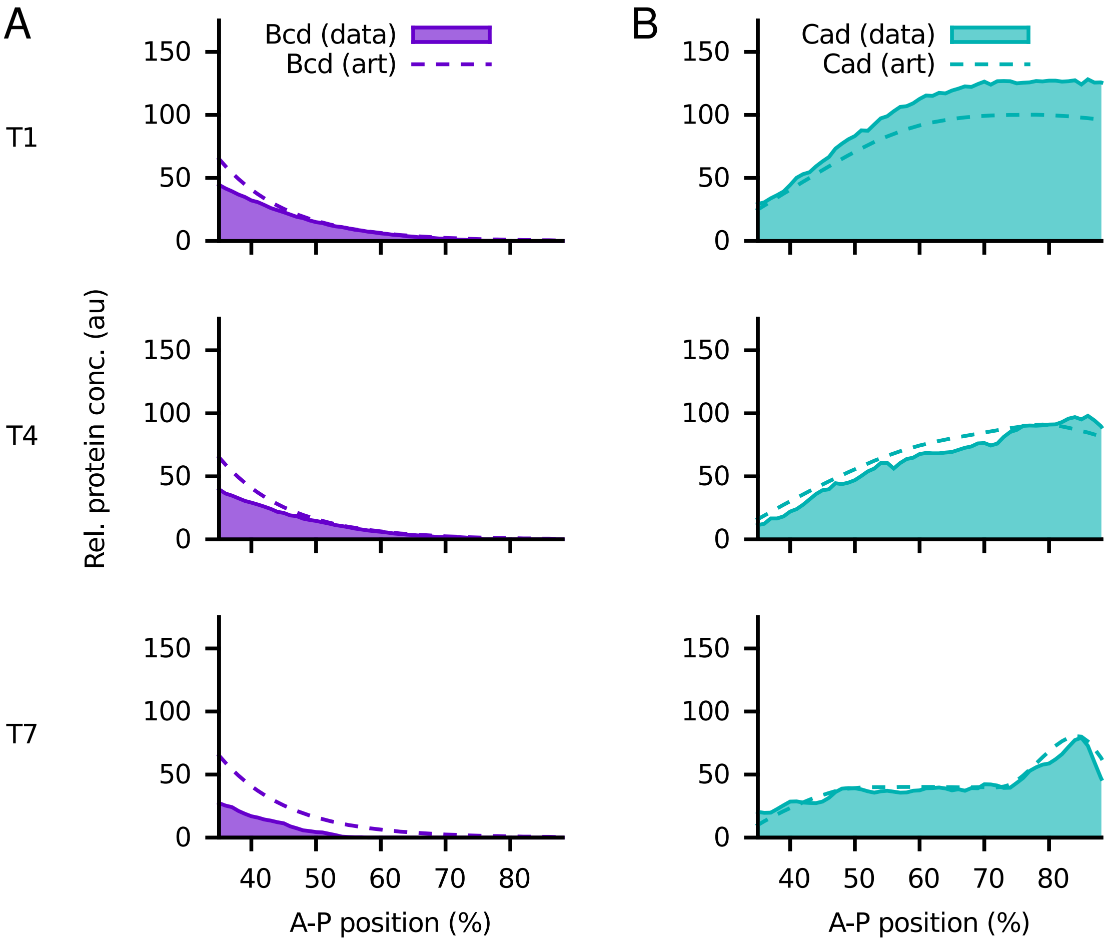

Figure S6. Measured and artificial maternal inputs. Plots show a comparison of protein-based expression (data) and approximated, artificial expression profiles (art) for the maternal gradients of Bcd and Cad. (A) Quantitative Bcd data (solid) was approximated using a (time-invariant) exponential function (dashed line). (B) Cad data (solid) was approximated using splines (dashed line; see Materials and Methods for details). Horizontal axes represent A–P position, where 0% is the anterior pole. Only the trunk region of the embryo included in our models is shown. Relative protein concentrations are in arbitrary units (au). T1/4/7 represent time classes during C14A. Time progresses downwards.
