# Supplementary material for: Efficient Reverse-Engineering of a Developmental Gene Regulatory Network
Source: PLoS Comput Biol. 2012 Jul 12;8(7):e1002589. doi: 10.1371/journal.pcbi.1002589 (PMC3395622; doi:10.1371/journal.pcbi.1002589)
Supplement: Table S1 — Drosophila mRNA vs. protein expression: boundary shifts. This table shows shifts in boundary positions from a starting point at T1, or, from the earliest appearance of the boundary domain. Numbers are in % A–P position. mRNA expression is shown in black, protein expression in grey. (−) indicates a shift to the anterior (+) a shift to the posterior. Drosophila values are calculated from [43]. Single dashes indicate boundaries that are not present at a given time point. Double dashes indicate boundaries that are observable, but were not measured in [43]. (PDF) [file pcbi.1002589.s007.pdf]

Table S1: *Drosophila* mRNA vs. protein expression: boundary shifts. This table shows shifts in boundary positions from a starting point at T1, or, from the earliest appearance of the boundary domain. Numbers are in % A–P position. mRNA expression is shown in black, protein expression in grey. (-) indicates a shift to the anterior (+) a shift to the posterior. *Drosophila* values are calculated from [1]. Single dashes indicate boundaries that are not present at a given time point. Double dashes indicate boundaries that are observable, but were not measured in [1].

**Domain**	***hb* ant**	***gt* anterior**		***Kr* central**		***kni* abdominal**		***gt* posterior**		***hb* posterior**	
**Boundary** / **Time class**	P	A	P	A	P	A	P	A	P	A	P
**T1**	43.8<br>45.9	20.7<br>16.9	39.3<br>38.0	45.3<br>44.3	57.8<br>62.4	56.5<br>58.8	70.1<br>74.0	69.8<br>72.7	82.2<br>90.1	85.0<br>--	-<br>-
**T2**	+0.6<br>-0.9	-0.7<br>+1.5	-1.1<br>-0.1	-1.0<br>-0.5	-0.5<br>-1.1	+1.4<br>-0.2	0.0<br>-2.6	0.0<br>-1.7	-2.8<br>-4.1	-3.8<br>--	89.7<br>--
**T3**	+1.3<br>-0.1	-2.0<br>+1.3	-1.5<br>0	+0.2<br>-0.1	+2.0<br>-2.2	+1.5<br>+0.2	-0.1<br>-3.3	-2.3<br>-3.4	-5.3<br>-7.8	-3.9<br>80.7	-2.9<br>93.0
**T4**	+3.2<br>+1.3	-2.5<br>+1.1	-1.6<br>+0.1	-1.3<br>-0.1	0.0<br>-2.3	+0.4<br>0.0	-3.0<br>-4.2	-2.8<br>-4.0	-5.2<br>-10.3	-6.1<br>-0.5	-4.2<br>-0.6
**T5**	+2.1<br>+0.9	-2.4<br>+1.2	-1.6<br>+0.3	-1.4<br>-0.4	-1.1<br>-2.8	-0.5<br>+0.4	-4.1<br>-4.6	-3.0<br>-4.7	-5.7<br>-11.6	-8.1<br>-1.2	-4.5<br>-1.7
**T6**	+2.0<br>+1.3	-1.9<br>+0.6	-2.0<br>-0.1	-1.8<br>-0.7	-1.4<br>-3.2	-2.0<br>-0.4	-5.3<br>-5.0	-4.2<br>-5.4	-7.1<br>-12.6	-10.0<br>-1.0	-5.6<br>-2.0
**T7**	+2.0<br>+1.2	-1.0<br>-1.6	-1.7<br>-0.4	-2.3<br>-1.1	-5.3<br>-4.5	-2.0<br>-0.9	-7.1<br>-5.7	-5.3<br>-5.9	-10.1<br>-13.7	-9.4<br>-1.4	-6.1<br>-3.5
**T8**	+1.2<br>+0.9	-0.2<br>+1.7	-2.1<br>-0.1	-2.6<br>-1.0	-5.3<br>-5.2	+1.8<br>-1.6	-6.5<br>-6.3	-4.5<br>-6.4	-9.4<br>-15.2	-9.5<br>-2.0	-6.4<br>-4.1

[1] Surkova S, Kosman D, Kozlov K, Manu, Myasnikova E, et al. (2008) Characterization of the *Drosophila* segment determination morphome. Dev Biol 313: 844-862
