# Supplementary material for: Efficient Reverse-Engineering of a Developmental Gene Regulatory Network
Source: PLoS Comput Biol. 2012 Jul 12;8(7):e1002589. doi: 10.1371/journal.pcbi.1002589 (PMC3395622; doi:10.1371/journal.pcbi.1002589)
Supplement: Table S2 — Drosophila mRNA versus protein expression: domain width. This table shows domain widths for trunk gap genes. Numbers are given in % egg length. mRNA expression is shown in black, protein expression in grey. Drosophila values are calculated from [43]. Single dashes indicate boundaries that are not present at a given time point. Double dashes indicate boundaries that are observable, but were not measured in [43]. (PDF) [file pcbi.1002589.s008.pdf]

Table S2: *Drosophila* mRNA versus protein expression: domain width. This table shows domain widths for trunk gap genes. Numbers are given in % egg length. mRNA expression is shown in black, protein expression in grey. *Drosophila* values are calculated from [1]. Single dashes indicate boundaries that are not present at a given time point. Double dashes indicate boundaries that are observable, but were not measured in [1].

**Domain / Time class**	***gt* anterior**	***Kr* central**	***kni* abdominal**	***gt* posterior**	***hb* posterior**
**C13**	20.5<br>--	12.7<br>--	13.7<br>--	26.0<br>--	10.5<br>-
**T1**	18.6<br>21.1	12.5<br>18.1	13.6<br>15.2	12.4<br>17.4	15.0<br>-
**T2**	18.2<br>19.5	13.0<br>17.5	12.2<br>12.8	9.6<br>15.0	11.0<br>--
**T3**	19.1<br>19.8	14.3<br>16.0	12.0<br>11.7	9.4<br>13.0	9.2<br>12.3
**T4**	19.5<br>20	13.8<br>15.9	10.2<br>11.0	10.0<br>11.1	9.1<br>12.2
**T5**	19.4<br>20.2	12.8<br>15.7	10.0<br>11.0	9.7<br>10.5	10.8<br>11.8
**T6**	18.5<br>20.4	12.9<br>15.6	10.3<br>10.6	9.5<br>10.2	11.6<br>11.3
**T7**	17.9<br>19.4	9.5<br>14.7	8.5<br>10.4	7.6<br>9.6	10.5<br>10.2
**T8**	16.7<br>19.6	9.8<br>13.9	8.9<br>10.5	7.5<br>8.6	10.3<br>10.2

[1] Surkova S, Kosman D, Kozlov K, Manu, Myasnikova E, et al. (2008) Characterization of the *Drosophila* segment determination morphome. Dev Biol 313: 844-862.
