# Supplementary material for: Efficient Reverse-Engineering of a Developmental Gene Regulatory Network
Source: PLoS Comput Biol. 2012 Jul 12;8(7):e1002589. doi: 10.1371/journal.pcbi.1002589 (PMC3395622; doi:10.1371/journal.pcbi.1002589)
Supplement: Text S1 — Common patterning defects in Gap Gene Circuits. (PDF) [file pcbi.1002589.s009.pdf]

Text S1: *Drosophila* Segmentation Gene Expression

This file documents the quantified mRNA expression patterns of *Drosophila melanogaster* segmentation genes used in this study. We first show a supplementary figure with expression patterns of maternal and terminal gap genes not presented in the main paper (part 1). Next, we describe maternal and gap gene expression in detail (part 2). We then introduce our scheme for naming and identifying each expression domain boundary (part 3). Finally, part 4 of this document presents tables with the raw data for measured boundary positions.

Table of Contents

1. Additional Expression Patterns: *bcd, cad, tll, hkb*

2. Detailed Description of Gene Expression Patterns

- 1.1. Maternal Expression Patterns
  - 1.1.1 *bicoid (bcd)*
  - 1.1.2 *caudal (cad)*
- 1.2 Terminal Gap Gene Expression
  - 1.2.1 *tailless (tll)*
  - 1.2.2 *huckebien (hkb)*
- 1.3 Trunk Gap Gene Expression
  - 1.3.1 *hunchback (hb)*
  - 1.3.2 *giant (gt)*
  - 1.3.3 *Krüppel (Kr)*
  - 1.3.4 *knirps (kni)*

3. Boundary Naming Scheme

4. Boundary Position Tables

1. Additional mRNA Expression Patterns: *bcd*, *cad*, *tll* and *hkb*

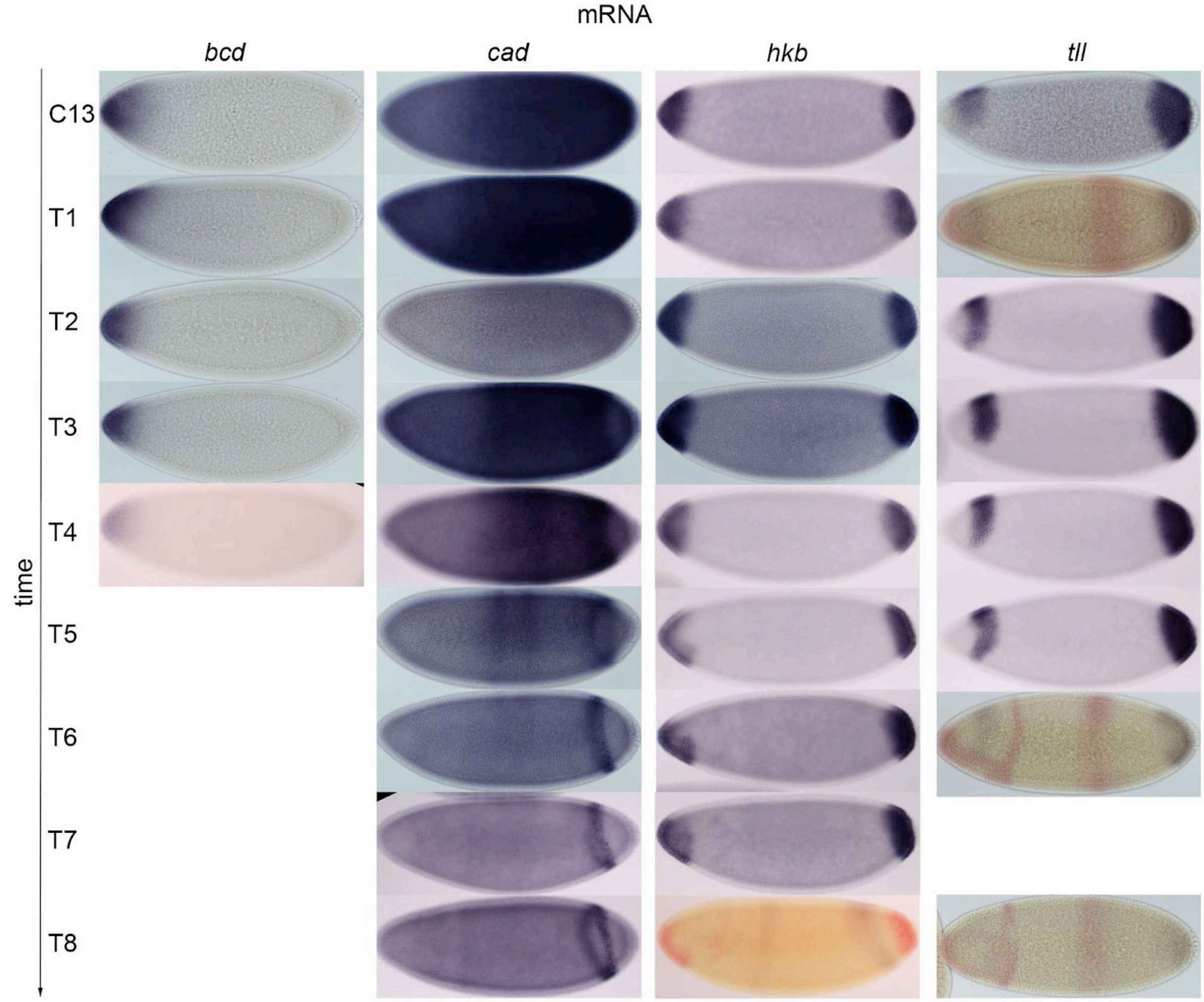

Figure S1.1: Expression data—additional genes in our data set. Time series of mRNA expression patterns of the maternal genes *bcd* and *cad,* as well as the terminal gap genes *hkb* and *tll* at cleavage cycle 14A (C14A) visualised by colorimetric (enzymatic) *in situ* hybridisation using wide-field microscopy. Relevant expression patterns are shown in blue, except for *hkb* at T8, which is shown in red. Red stain in *tll* (T1/6/8) shows *kni.* Single stains are shown for all other genes and time classes. Early stages at the top, time progresses downwards. Embryos are arranged anterior to the left, dorsal up. T1–8 indicate time classes subdividing C14A (see Materials and Methods for details). Expression patterns of *hb, Kr, gt,* and *kni* are shown in Figure 3 of the main paper.

2. Detailed Description of Gene Expression Patterns

1.1. Maternal Expression Patterns

1.1.1 *bicoid (bcd)*

We detect *bcd* transcripts from cleavage cycle 1 (C1) until time class 4 of cleavage cycle 14A (T4). During these stages, *bcd* transcripts show a steep graded expression pattern at the anterior pole. Most transcripts are localised within the anterior-most 14% of the embryo in agreement with [1,2]. We do not detect significant amounts of transcripts in regions further posterior (as previously reported in [3,4]). During C14A, *bcd* transcripts fade and disappear by T5 (Figure S1.1) [2].

1.1.2 *caudal (cad)*

We detect uniform *cad* transcripts throughout the embryo until C13, when they begin to fade in the anterior 34% of the embryo (not shown). During C14A, *cad* transcripts also gradually disappear in most of the central region until they can only be detected posterior of 61% A–P (Figure S1.1). Around T5, expression also retracts from the posterior pole to around 87% A–P position. By T6 a sharp posterior stripe has formed at about 82–87% A–P position (Figure S1.1). This stripe shifts slightly to about 85–89% A–P position at T8 (Figure S1.1). The observed *cad* expression dynamics are consistent with previously published evidence [5–8]. Note that the posterior stripe of *cad* has a homeotic, rather than segment-determination function [8].

1.2 Terminal Gap Gene Expression

1.2.1 *tailless (tll)*

We did not assess *tll* expression before C13. At that stage, it can be detected in an anterior head stripe (8–16% A–P position), and a domain at the posterior pole extending to 84% A–P position. In contrast to earlier reports [9,10], we observe that anterior *tll* has already retracted from the anterior pole by C13. The posterior domain slowly sharpens and narrows to about 89% A–P position during C14A (Figure S1.1). The anterior head stripe retracts from the ventral region and shifts slightly to the posterior until it is found at 15–21% A–P position at T6. Apart from the slight discrepancy concerning the timing of retraction of the anterior domain, our observations are consistent with earlier studies of *tll* expression [9,10].

1.2.2 *huckebein (hkb)*

We did not assess *hkb* expression before C13. At that stage it can be detected at the anterior and posterior pole respectively. The anterior domain extends to 9%, the posterior domain to 91% embryo length (not shown). Expression in the anterior pole narrows slowly to around 6% embryo length by T8 (Figure S1.1). The extent of the posterior pole varies around 88–90% A–P position between T1 and T8, but does not show any systematic shift or narrowing (Figure S1.1). This is consistent with previously published evidence [11,12].

1.3 Trunk Gap Gene Expression

1.3.1 *hunchback (hb)*

We did not assess maternal and zygotic *hb* expression before C12. At that stage, *hb* transcripts can be detected in the anterior of the embryo extending from the pole to 45% A–P position (not shown). By C13 some embryos show a weakening of expression at the anterior pole. This process continues through C14A, and results in the formation of an anterior stripe (the PS4 stripe, [13]) by T3 at 45% A–P position, with diffuse, weaker expression anterior to it (Figure 3 of the main paper). The posterior boundary of this anterior domain shifts slightly during early C14A, but overall remains at roughly the same position through time. At C13 a small posterior domain appears at 90% A–P position. By T2 its anterior border has shifted to 81% A–P position, and the domain has retracted from the posterior pole (to 92% A–P position) forming a band with a width of about 11% embryo length (Figure 3 in the main paper). This posterior domain keeps shifting towards the anterior reaching 75–86% A–P position by T8, and reducing its width slightly to 10% embryo lenght. Our data are in agreement with an earlier qualitative description of *hb* expression [13]. The position of the posterior boundary of anterior *hb* is located slightly further anterior in our data than reported previously [14,15]. This is probably due to the fact that the position of boundaries was measured slightly differently in each study.

1.3.2 *giant (gt)*

We did not assess *gt* expression before C13. At that stage, we detect *gt* transcripts in an anterior stripe at 19–40% A–P position, and a posterior domain extending from 74% A–P position to the posterior pole (not shown). At T1 the posterior domain retracts from the posterior pole forming a band at 70–82% A–P position (with a width of 12% embryo length; see Figure 3 in the main paper). Both of these boundaries shift to the anterior over time until the domain is positioned at 65–73% A–P position (width: 7.5% embryo length) at T8 (Figure 3 in the main paper). At T3 the anterior domain of *gt* starts to split, and forms two separate stripes at C14A-

T4. At T5 an additional band (called the head patch) appears at the anterior pole. These three anterior expression stripes persist until T8 with the posterior-most boundary remaining around 37% A–P position throughout (Figure 3 of the main paper). Our observations are consistent with previously published studies of *gt* expression [16,17], and our measurements of domain boundary positions at C13 correspond to those based on fluorescent staining data [18].

1.3.3 *Krüppel (Kr)*

We did not assess *Kr* expression before C13, when its transcripts can be detected in a central domain between 47 and 59% A–P position (not shown). By T1 this domain has shifted slightly to the anterior to 45–58% A–P position (see Figure 3 of the main paper). By T8 there is a minor shift in the anterior border to 43% A–P position, while a stronger shift is seen at the posterior border to 53% A–P position. This reduces the domain width slightly from 13% to 10% embryo length from C13 to T8. At T5 an anterior head domain arises at 11–17% A–P position, and gradually moves to the posterior occupying 17–22% A–P position by T8 (Figure 3 of the main paper). Finally, at T6, a posterior domain arises in the posterior pole region at 90% A–P position. This domain moves slightly to the anterior until it reaches 87% A–P position by T8 (Figure 3 of the main paper). These data are consistent with earlier reports on *Kr* expression [19,20], and our measurements of domain boundary positions at C13 correspond to those based on fluorescent staining data [18].

1.3.4 *knirps (kni)*

We did not assess *kni* expression before C13. At that stage, *kni* transcripts can be detected at the anterior pole extending to 9% A–P position, and in an abdominal domain at 58–72% A–P position (see Figure 3 of the main paper). Through C14A, the anterior boundary of the abdominal domain shifts to the anterior reaching 55% A–P position at C14A-T8. A larger shift to the anterior is seen for the posterior boundary shifts which moves from from 70% A–P position at T1 to 66% A–P position by T8. This results in a linear narrowing of the boundary from 14% embryo length at C13 to 10% at C14A-T4 and finally 9% at C14A-T8. In the anterior, expression at the pole remains at around 8-10% A–P position while a head stripe appears at C14A-T4 at 27-30% A–P position and is maintained up to C14A-T8. These observations are consistent with earlier studies on *kni* expression [21,22], and our measurements of domain boundary positions at C13 correspond to those based on fluorescent staining data [18].

3. Boundary Naming Scheme

To uniquely identify expression features, we enumerate each expression boundary for each gene starting from 0 at the most anterior boundary. The resulting naming scheme is shown in Figure S1.2. Measured positions of boundaries through time are reported in Part 4 below (see also Table 1 of the main text, and Supplementary Tables 4 and 5 for comparisons to protein expression data).

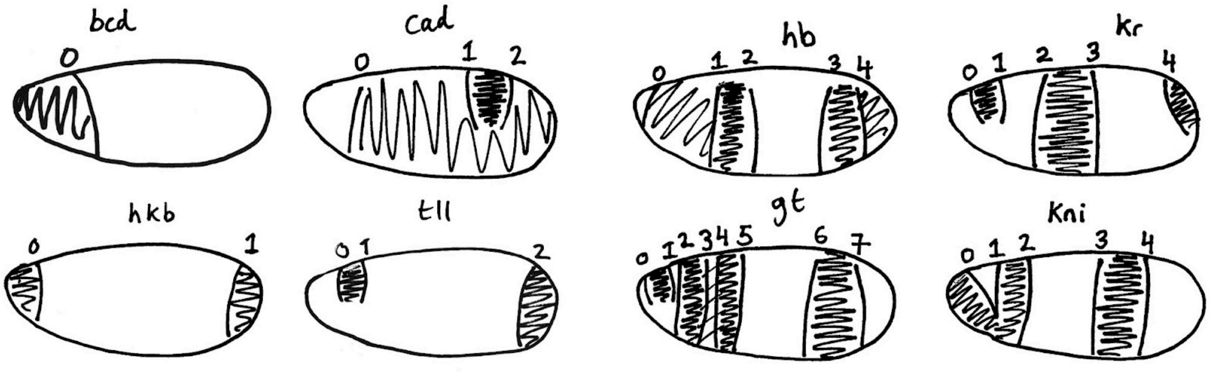

Figure S1.2. Boundary naming scheme. This figure displays schematic drawings of embryos showing expression boundary numbers and relative positions for each gene considered in this study. Maternal inputs (*bcd, cad*) and terminal gap genes (*tll, hkb*) are shown on the left. Trunk gap genes (*hb, Kr, gt, kni*) are shown on the right. Embryos drawn anterior to the left, dorsal up.

4. Boundary Position Tables

These tables show positions of boundaries along the A–P axis (in %, where 0% is the anterior pole) for all expression boundaries in Figure S1.2. Boundary positions correspond to the position of the splines' inflection points (see Materials and Methods). Our data cover cleavage cycles 13 (C13) and C14A. Time points are sorted into canonical time classes C13, and C14A-T1 to T8 (see Materials and Methods in the main text for details). We also show time in minutes since the beginning of C13. See Table 1 of the main text, and Supplementary Tables 4 and 5 for comparisons to protein expression data.

**Gene: *bcd***		
Time class	time (min)	0
c13	10.55	14.294
T1	24.225	12.986
T2	30.475	11.42
T3	36.725	12.386
T4	42.975	13.66

**Gene: *cad***				
Time class	time (min)	0	1	2
c13	10.55	34.373	na	na
T1	24.225	29.138	na	na
T2	30.475	38.721	na	na
T3	36.725	37.794	na	na
T4	42.975	39.762	na	na
T5	49.225	39.052	na	86.725
T6	55.475	na	81.945	86.575
T7	61.725	na	82.131	86.008
T8	67.975	na	85.15	88.609

**Gene: *tll***				
Time class	time (min)	0	1	2
c13	10.55	8.287	16.143	84.214
T1	24.225	na	na	89.224
T2	30.475	11.578	18.976	86.589
T3	36.725	12.044	19.886	83.231
T4	42.975	14.554	20.431	84.663
T5	49.225	15.404	22.98	87.718
T6	55.475	15.022	20.703	88.08

**Gene: *hkb***			
Time class	time (min)	0	1
c13	10.55	8.916	90.662
T1	24.225	9.881	89.796
T2	30.475	9.61	89.014
T3	36.725	11.403	88.127
T4	42.975	10.477	89.05
T5	49.225	9.348	89.24
T6	55.475	9.825	88.992
T7	61.725	9.547	89.802
T8	67.975	6.436	90.147

Gene: ***hb***						
Time class	time (min)	0	1	2	3	4
c13	10.550	na	na	45.777	89.521	na
T1	24.225	na	na	43.812	85.021	na
T2	30.475	11.927	na	44.375	81.199	92.213
T3	36.725	13.681	na	45.124	80.096	89.258
T4	42.975	12.310	41.730	47.057	78.851	87.973
T5	49.225	na	40.697	45.865	76.916	87.732
T6	55.475	na	40.932	45.753	75.001	86.637
T7	61.725	na	40.851	45.758	75.633	86.094
T8	67.975	na	40.466	45.024	75.476	85.75

Gene: ***gt***									
Time class	time (min)	0	1	2	3	4	5	6	7
c13	10.55	na	na	19.294	na	na	39.755	73.977	na
T1	24.225	na	na	20.679	na	na	39.301	69.814	82.19
T2	30.475	na	na	20.025	na	na	38.239	69.816	79.43
T3	36.725	na	na	18.668	na	29.592	37.789	67.453	76.937
T4	42.975	na	na	18.211	22.819	29.166	37.691	66.948	77.042
T5	49.225	5.696	10.142	18.28	23.748	30.004	37.726	66.84	76.481
T6	55.475	5.955	10.297	18.826	24.284	31.244	37.309	65.596	75.076
T7	61.725	7.172	11.631	19.735	24.537	31.299	37.626	64.513	72.078
T8	67.975	8.4	13.043	20.539	25.001	32.857	37.224	65.346	72.824

Gene: ***Kr***						
Time class	time (min)	0	1	2	3	4
c13	10.55	na	na	46.729	59.368	na
T1	24.225	na	na	45.269	57.817	na
T2	30.475	na	na	44.251	57.334	na
T3	36.725	na	na	45.471	59.756	na
T4	42.975	na	12.714	44.024	57.777	na
T5	49.225	11.059	17.112	43.9	56.655	na
T6	55.475	13.112	18.166	43.461	56.405	90.385
T7	61.725	13.981	19.204	42.977	52.498	87.963
T8	67.975	16.743	22.444	42.685	52.509	87.364

Gene: ***kni***						
Time class	time (min)	0	1	2	3	4
c13	10.55	9.369	na	na	58.277	71.951
T1	24.225	9.139	na	na	56.481	70.132
T2	30.475	10.545	na	na	57.915	70.087
T3	36.725	10.235	na	na	58.04	70.003
T4	42.975	10.37	26.518	30.453	56.936	67.057
T5	49.225	9.222	25.647	29.812	55.997	66.021
T6	55.475	7.8	26.968	31.091	54.522	64.767
T7	61.725	8.113	27.387	31.552	54.469	62.97
T8	67.975	8.526	27.971	30.847	54.693	63.61

References

1. Berleth T, Burri M, Thoma G, Bopp D, Richstein S, Frigerio G, Noll M, Nüsslein-Volhard C (1988). The role of localization of *bicoid* RNA in organizing the anterior pattern of the *Drosophila* embryo. EMBO J 7: 1749–56.
2. Little SC, Tkacik G, Kneeland TB, Wieschaus EF, Gregor T (2011). The formation of the Bicoid morphogen gradient requires protein movement from anteriorly localized mRNA. PLoS Biol 9: e1000596.
3. Frigerio G, Burri M, Bopp D, Baumgartner S, Noll M (1986). Structure of the segmentation gene *paired* and the Drosophila PRD gene set as part of a gene network. Cell 47: 735–45.
4. Spirov A, Fahmy K, Schneider M, Frei E, Noll M, Baumgartner S (2009). Formation of the *bicoid* morphogen gradient: an mRNA gradient dictates the protein gradient. Development 136: 605–14.
5. Mlodzik M, Fjose A, Gehring WJ (1985). Isolation of *caudal*, a *Drosophila* homeo box-containing gene with maternal expression, whose transcripts form a concetration gradient at the pre-blastoderm stage. EMBO J 4: 2691–9.
6. Mlodzik M, Gehring WJ (1987). Expression of the *caudal* gene in the germ line of Drosophila: formation of an RNA and protein gradient during early embryogenesis. Cell 48: 465–478.
7. Zygotic *caudal* regulation by *hunchback* and its role in abdominal segment formation of the *Drosophila* embryo. Development 121: 1023–8.
8. Moreno E, Morata G (1999). *Caudal* is the Hox gene that specifies the most posterior *Drosophila* segment. Nature 400: 873–7.
9. Pignoni F, Baldarelli RM, Steingrimsson E, Diaz RJ, Patapoutian A, Merriam JR, Lengyel JA (1990). The Drosophila gene *tailless* is expressed at the embryonic termini and is a member of the steroid receptor superfamily. Cell 62: 151–63.
10. Pignoni F, Steingrimsson E, Lengyel JA (1992). *bicoid* and the terminal system activate *tailless* expression in the early *Drosophila* embryo. Development 115: 239–51.
11. Brönner G, Jäckle H (1991). Control and function of terminal gap gene activity in the posterior pole region of the *Drosophila* embryo. Mech Dev 35: 205–211.
12. Brönner G, Chu-LaGraff Q, Doe CQ, Cohen B, Weigel D, Taubert H, Jäckle H (1994). Sp1/egr-like zinc-finger protein required for endoderm specification and germ-layer formation in *Drosophila.* Nature 369: 664–8.
13. Tautz D, Lehmann R, Schnürch H, Schuh R, Seifert E, Kienlin A, Jones K, Jäckle H (1987). Finger protein of novel structure encoded by *hunchback*, a second member of the gap class of *Drosophila* segmentation genes. Nature 327: 383–9.
14. Houchmandzadeh B, Wieschaus E, Leibler S (2002). Establishment of developmental precision and proportions in the early *Drosophila* embryo. Nature 415: 798–802.
15. Crauk O, Dostatni N (2005). Bicoid determines sharp and precise target gene expression in the *Drosophila* embryo. Curr Biol 15: 1888–98.
16. Mohler J, Eldon ED, Pirrotta V (1989). A novel spatial transcription pattern associated with the segmentation gene, *giant,* of *Drosophila.* EMBO J 8: 1539–48.
17. Kraut R, Levine M (1991). Spatial regulation of the gap gene *giant* during *Drosophila* development. Development 111: 601–9.
18. Jaeger J, Sharp DH, Reinitz J (2007) Known maternal gradients are not sufficient for the establishment of gap domains in *Drosophila melanogaster*. Mech Dev 124: 108–128.
19. Knipple DC, Seifert E, Rosenberg UB, Preiss A, Jäckle H (1985). Spatial and temporal patterns of *Krüppel* gene expression in early *Drosophila* embryos. Nature 317: 40–4.
20. Harding K, Levine M (1988). Gap genes define the limits of Antennapedia and Bithorax gene expression during early development in *Drosophila.* EMBO J 7: 205–14.
21. Rothe M, Nauber U, Jäckle H (1989). Three hormone receptor-like *Drosophila* genes encode an identical DNA-binding finger. EMBO J 8: 3087–94.
22. Rothe M, Wimmer EA, Pankratz MJ, González-Gaitán M, Jäckle H (1994). Identical transacting factor requirement for *knirps* and *knirps-related* gene expression in the anterior but not in the posterior region of the *Drosophila* embryo. Mech Dev 46: 169–81.
