# Supplementary material for: Efficient Reverse-Engineering of a Developmental Gene Regulatory Network
Source: PLoS Comput Biol. 2012 Jul 12;8(7):e1002589. doi: 10.1371/journal.pcbi.1002589 (PMC3395622; doi:10.1371/journal.pcbi.1002589)
Supplement: Text S2 — Drosophila Segmentation Gene Expression. (PDF) [file pcbi.1002589.s010.pdf]

Text S2: Common Patterning Defects in Gap Gene Circuits

Gene circuit models were selected for further analysis as follows (see Materials and Methods for details): after passing several numerical stability tests, circuits were checked for patterning defects by means of visual inspection. Circuits showing any obvious and drastic deviation between model output and data were discarded. The five most commonly observed defects are shown in Figure S2.1. Below, we focus on fits to the full data set (either mRNA or protein) using OLS and WLS cost functions. The same classes of defects also occurred in fits to reduced data sets (not shown).

OLS solutions exhibit a wide range of very frequent defects. One very common problem consists of the late ectopic expression of Kr and/or kni at the posterior end (Figure S2.1, rows 1 and 2). The ectopic Kr domain has a very small inhibitory effect on the posterior domain of hb, while kni causes a strong decrease in hb activation in the posterior. In some of the solutions, this leads to significant hb down-regulation at the late blastoderm stage (T6–T8). This defect can also be observed in protein-based gene circuits fit with the OLS cost function [1].

In addition, we often observe bimodal expression domains for kni, gt and posterior hb. These domains show two peaks of maximum expression instead of one (Figure S2.1, row 3).

Another defect consists of low expression of Kr and gt at late blastoderm stage (Figure S2.1, row 4). Unlike other defects described here, this problem can vary very strongly in its severity. Only circuits that show a significant reduction of these two domains were excluded from further analysis.

Finally, two minor defects consisted of slightly extended expression domains and/or late low expression of Kr and gt. Depending on the severity of these two defects in a gene circuit, we accepted or discarded the circuit for further analysis (Figure S2.1, two bottom panels).

In contrast to OLS solutions, we observed much fewer and less frequent defects in gene circuits obtained by WLS fits. Discarded WLS runs mainly show bimodal expression domains, and low levels of expression of Kr and gt at late stages (Figure S2.1, rows 3 and 4). Late ectopic posterior expression domains of Kr and/or kni were very rare. In the few cases that did show low ectopic expression, it did not affect the dynamics in a significant way (data not shown). We did not observe any low-level extensions to expression domains.

Finally we contrasted defects observed in mRNA-based gene circuits to those in circuits based on fits to protein data from [1]. We observe that protein-based circuits consistently show two little defects that are absent from our mRNA circuits: one affects the left shoulder of the abdominal kni domain at time class T1, the other shows a slightly bimodal posterior hb domain from T1 to T5 (see also Figure 5B in the main text).

References

[1] Ashyraliyev M, Siggens K, Janssens H, Blom J, Akam M, et al. (2009) Gene circuit analysis of the terminal gap gene huckebein. PLoS Comput Biol 5: e1000548.

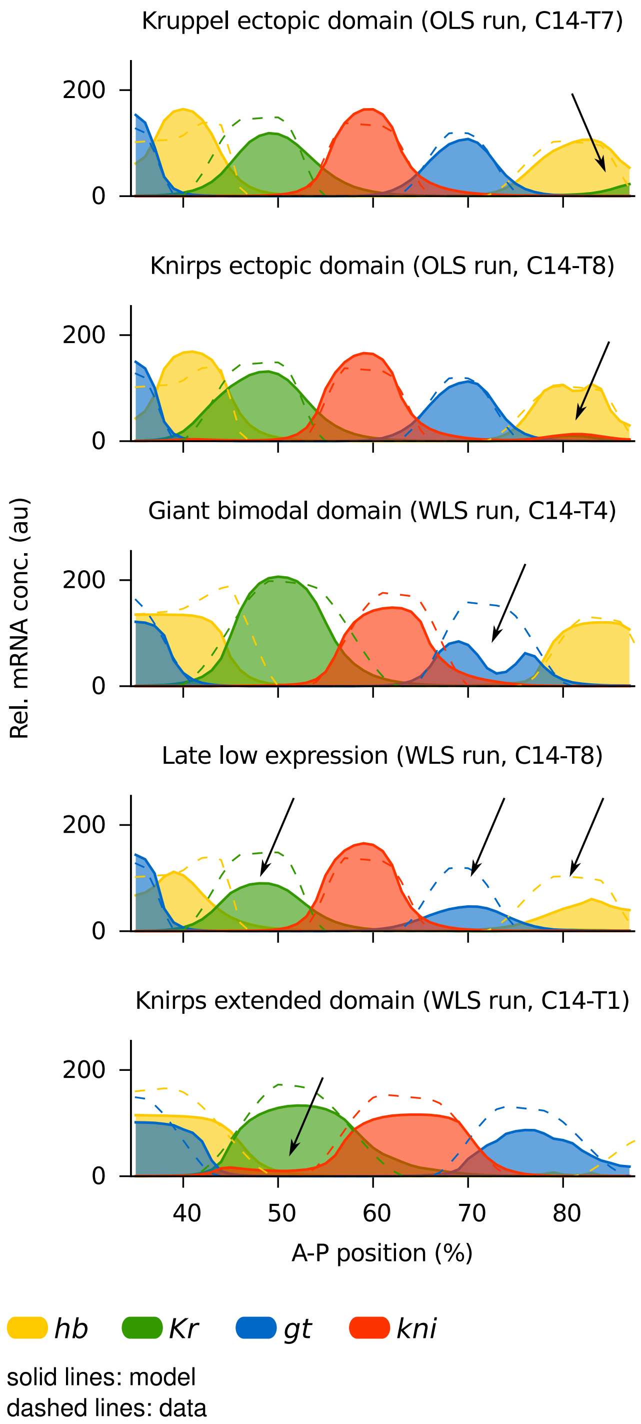

Figure S2.1. The five most commonly observed patterning defects in gene circuits. Deviations in model output from data used for fitting are highlighted by arrows. In the top panel, an ectopic posterior Kr domain (starting at around 85% A–P position) can be seen. The second panel shows an ectopic posterior kni domain (peaking at around 82% A–P position). In the third panel, a bimodal expression domain is shown (the posterior gt domain in this case; bimodal domains are also observed for kni and posterior hb). The fourth panel displays low expression of Kr, posterior gt and hb at late blastoderm stages (shown for T8). The bottom panel shows a low-level anterior extension of the abdominal kni domain (around 50% A–P position). Horizontal axes represent A–P position, where 0% is the anterior pole. Relative mRNA concentrations are in arbitrary units (au).
