# Supplementary material for: Efficient Reverse-Engineering of a Developmental Gene Regulatory Network
Source: PLoS Comput Biol. 2012 Jul 12;8(7):e1002589. doi: 10.1371/journal.pcbi.1002589 (PMC3395622; doi:10.1371/journal.pcbi.1002589)
Supplement: Text S3 — Genetic Interconnectivity Matrices. (PDF) [file pcbi.1002589.s011.pdf]

Text S3: Genetic interconnectivity matrices

Below, we show genetic interconnectivity matrices for the different scenarios of data reduction described in Table 3 of the main text. The tables here form the basis of summary Figure 7 in the main text (with the exception of the interactions involved in the shift mechanism, see main text for more information).

In all figures, triplet numbers (i/n/a) indicate number of gene circuits with inhibiting (red), neutral (blue) and activating (green) interactions for each series of optimisation runs listed in Table 3 of the main text. In case all gene circuits show equal behaviour, a dark colour is used, otherwise a lighter shade is applied.

WLS

	hb	*Kr*	*gt*	*kni*	*bcd*	*cad*	*tll*	*hkb*
hb	1/0/51	52/0/0	51/0/1	52/0/0	0/0/52	1/0/51	0/0/52	52/0/0
Kr	51/1/0	45/7/0	52/0/0	52/0/0	0/0/52	0/0/52	52/0/0	–
gt	52/0/0	52/0/0	0/0/52	22/30/0	0/0/52	0/0/52	52/0/0	–
kni	52/0/0	52/0/0	52/0/0	0/0/52	0/0/52	0/1/51	52/0/0	–

OLS

	hb	*Kr*	*gt*	*kni*	*bcd*	*cad*	*tll*	*hkb*
hb	0/0/10	10/0/0	9/0/1	10/0/0	1/0/9	1/4/5	0/0/10	9/0/1
Kr	3/5/2	3/4/3	10/0/0	10/0/0	0/0/10	0/0/10	10/0/0	–
gt	10/0/0	10/0/0	0/0/10	0/1/9	0/0/10	0/0/10	10/0/0	–
kni	10/0/0	10/0/0	10/0/0	0/0/10	1/0/9	0/0/10	6/3/1	–

Figure S3.1. Weighted Least Squares (WLS) and Ordinary Least Squares (OLS) genetic interconnectivity matrices (as shown in Figure 6B of the main paper).

80% data

	hb	*Kr*	*gt*	*kni*	*bcd*	*cad*	*tll*	*hkb*
hb	1/2/31	34/0/0	34/0/0	34/0/0	0/0/34	0/1/33	0/0/34	33/1/0
Kr	33/0/1	27/4/3	34/0/0	34/0/0	0/1/33	0/0/34	34/0/0	–
gt	34/0/0	34/0/0	0/0/34	13/19/2	0/0/34	0/0/34	33/1/0	–
kni	34/0/0	34/0/0	34/0/0	0/0/34	1/0/33	0/1/33	33/0/1	–

60% data

	hb	*Kr*	*gt*	*kni*	*bcd*	*cad*	*tll*	*hkb*
hb	0/0/37	37/0/0	37/0/0	37/0/0	1/0/36	0/1/36	1/0/36	36/0/1
Kr	35/1/1	26/5/6	37/0/0	37/0/0	0/0/37	0/0/37	36/0/1	–
gt	37/0/0	37/0/0	0/1/36	14/21/2	0/0/37	0/0/37	36/1/0	–
kni	37/0/0	37/0/0	37/0/0	0/0/37	2/0/35	0/0/37	36/0/1	–

40% data

	hb	*Kr*	*gt*	*kni*	*bcd*	*cad*	*tll*	*hkb*
hb	0/0/21	21/0/0	19/0/2	21/0/0	0/0/21	2/1/18	0/1/20	19/0/2
Kr	17/3/1	7/1/13	21/0/0	21/0/0	2/0/19	0/0/21	21/0/0	–
gt	21/0/0	21/0/0	0/0/21	6/14/1	0/0/21	0/0/21	21/0/0	–
kni	21/0/0	21/0/0	21/0/0	0/0/21	5/1/15	0/1/20	20/1/0	–

20% data – no results

Figure S3.2. Genetic interconnectivity matrices for the data reduction scenarios. Individual boundaries have been removed such that 80, 60, 40 and 20% of the original data remain, with the requirement that expression domains remain intact. That is to say, at least one individual boundary is present to generate a correct integrated gene expression pattern.

7 time classes

	hb	*Kr*	*gt*	*kni*	*bcd*	*cad*	*tll*	*hkb*
hb	2/1/37	40/0/0	40/0/0	40/0/0	0/0/40	0/0/40	0/0/40	40/0/0
Kr	39/1/0	33/7/0	40/0/0	40/0/0	0/0/40	0/0/40	40/0/0	–
gt	40/0/0	40/0/0	0/0/40	12/28/0	0/0/40	0/1/39	40/0/0	–
kni	40/0/0	40/0/0	40/0/0	0/0/40	2/0/38	0/0/40	40/0/0	–

5 time classes

	hb	*Kr*	*gt*	*kni*	*bcd*	*cad*	*tll*	*hkb*
hb	0/1/23	24/0/0	23/0/1	24/0/0	0/0/24	1/0/23	0/0/24	24/0/0
Kr	24/0/0	21/3/0	24/0/0	24/0/0	0/0/24	0/0/24	23/1/0	–
gt	24/0/0	24/0/0	0/2/22	14/10/0	0/0/24	0/0/24	24/0/0	–
kni	24/0/0	24/0/0	24/0/0	0/0/24	3/1/20	0/0/24	24/0/0	–

3 time classes

	hb	*Kr*	*gt*	*kni*	*bcd*	*cad*	*tll*	*hkb*
hb	1/1/11	13/0/0	13/0/0	13/0/0	0/0/13	0/0/13	1/0/12	12/0/1
Kr	13/0/0	6/3/4	13/0/0	13/0/0	0/0/13	0/1/12	12/1/0	–
gt	13/0/0	13/0/0	0/2/11	10/3/0	0/0/13	0/0/13	13/0/0	–
kni	13/0/0	10/3/0	13/0/0	0/4/9	7/0/6	0/1/12	13/0/0	–

Figure S3.3. Genetic interconnectivity matrices for the time class removal scenarios. From the 9 time classes that the original data set is compromised of, we randomly remove time classes – with the constraint that the starting and ending time class remain – such that 7, 5 and 3 time classes remain. In this manner we test how the reverse engineering protocol depends on the number of data points over time.

maternal

	hb	*Kr*	*gt*	*kni*	*bcd*	*cad*	*tll*	*hkb*
hb	0/1/26	27/0/0	27/0/0	27/0/0	0/0/27	0/0/27	0/0/27	27/0/0
Kr	26/1/0	26/1/0	27/0/0	27/0/0	0/0/27	0/0/27	27/0/0	–
gt	27/0/0	27/0/0	11/13/3	27/0/0	0/0/27	0/0/27	27/0/0	–
kni	27/0/0	27/0/0	27/0/0	1/6/20	1/0/26	0/0/27	27/0/0	–

terminal

	hb	*Kr*	*gt*	*kni*	*bcd*	*cad*	*tll*	*hkb*
hb	2/3/16	21/0/0	19/2/0	21/0/0	0/0/21	0/0/21	3/5/13	21/0/0
Kr	21/0/0	19/2/0	21/0/0	21/0/0	0/0/21	0/0/21	15/3/3	–
gt	21/0/0	21/0/0	0/0/21	1/18/2	0/0/21	0/0/21	2/17/2	–
kni	21/0/0	21/0/0	21/0/0	0/0/21	6/0/15	0/0/21	21/0/0	–

materminal

	hb	*Kr*	*gt*	*kni*	*bcd*	*cad*	*tll*	*hkb*
hb	0/0/19	19/0/0	19/0/0	19/0/0	0/0/19	0/0/19	11/7/1	19/0/0
Kr	19/0/0	16/3/0	19/0/0	19/0/0	0/0/19	0/0/19	19/0/0	–
gt	19/0/0	19/0/0	0/9/10	18/1/0	0/0/19	0/0/19	18/1/0	–
kni	19/0/0	19/0/0	19/0/0	0/0/19	9/1/9	0/0/19	19/0/0	–

Figure S3.4. Genetic interconnectivity matrices for the scenarios with external inputs substituted with artificial inputs. 'maternal' refers to the substitution of Bcd and Cad expression patterns with artificially generated expression profiles. 'terminal' refers to the replacement of Tll and Hkb protein expression patterns by the mRNA expression patterns. 'materminal' replaces all four external inputs – Bcd, Cad, Tll and Hkb – by their artificial versions.
