# Supplementary material for: Efficient Reverse-Engineering of a Developmental Gene Regulatory Network
Source: PLoS Comput Biol. 2012 Jul 12;8(7):e1002589. doi: 10.1371/journal.pcbi.1002589 (PMC3395622; doi:10.1371/journal.pcbi.1002589)
Supplement: Text S4 — Dependent and independent parameter confidence intervals. (PDF) [file pcbi.1002589.s012.pdf]

Text S4: Dependent and independent parameter confidence intervals

Below we show plots with confidence intervals that form the basis of our parameter determinability analysis (summarised in Table 4 of the main text). We studied five scenarios that differ in data origin (protein or mRNA), data processing methods, weight computations for WLS, and/or the size of the embryo region being modelled. The five scenarios can be summarised as follows:

1. We approximated the protein data from [1] by processing them in a manner similar to our mRNA data. Splines were fit interactively to expression domain boundaries in integrated protein data (see Materials and Methods, and Figure 2 of the main paper). The resulting expression profiles were rescaled in space and time (post-processing steps as described in Materials and Methods, and Supplementary Figure 1). Weights for the WLS scoring function were then calculated from rescaled protein profiles, as described for mRNA data in Materials and Methods. This data set covers 58 nuclei from 35 to 92% A–P position. We performed 90 optimisation runs using the WLS cost function with this data set. 12 solutions were selected for further analysis (see Figures S12.1 and S12.2).

2. We used the protein data set from [1], replacing the original weights–derived from the measured variance of protein expression–by approximated weights as calculated for mRNA data (see Materials and Methods). This data set covers 58 nuclei from 35 to 92% A–P position. We performed 90 optimisation runs using the WLS cost function with this data set. 10 solutions were selected for further analysis (see Figures S12.3 and S12.4).

3. This is the full mRNA data set, fit with the OLS cost function (see main text for details). It covers 53 nuclei from 35 to 87% A–P position. 18 solutions were analysed (see Figures S12.5 and S12.6).

4. This is the full mRNA data set, fit with the WLS cost function (see main text for details). It covers 53 nuclei from 35 to 87% A–P position. 32 solutions were analysed (see Figures S12.7 and S12.8).

5. We extended the full mRNA data set by 5 additional nuclei at the posterior end. Thus, this data set covers 58 nuclei (from 35 to 92% A–P position) instead of the 53 nuclei (from 35 to 87% A–P position) included in (3) and (4). We performed 60 optimisation runs using the WLS cost function with this data set. 32 solutions were selected for further analysis (see Figures S12.9 and S12.10).

References

[1] Ashyraliyev M, Siggens K, Janssens H, Blom J, Akam M, et al. (2009) Gene circuit analysis of the terminal gap gene huckebein. PLoS Comput Biol 5: e1000548.

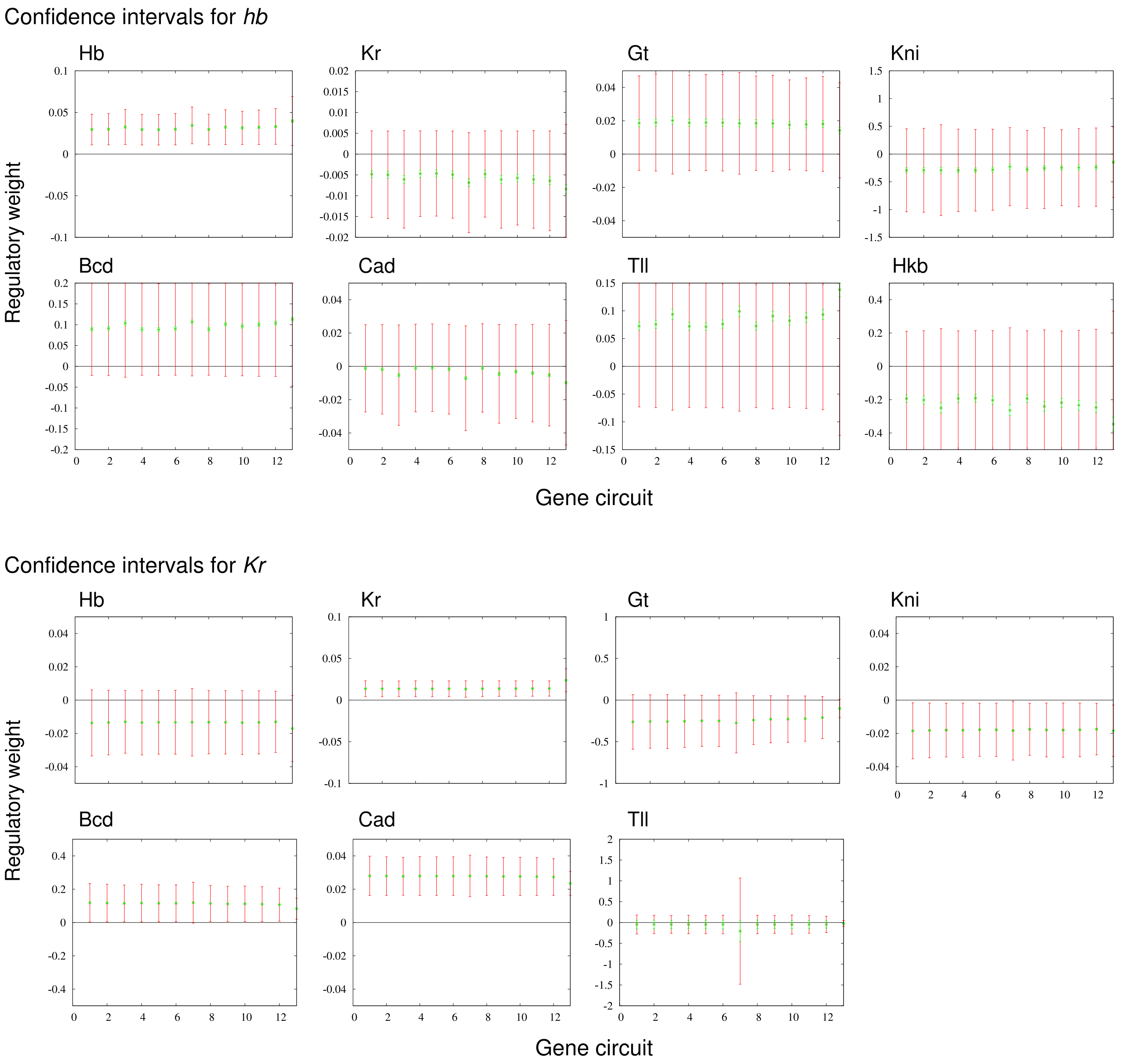

Figure S4.1. Confidence intervals for parameter determinability analysis. Fits to protein data approximated by our mRNA data processing method (data set 1). Plots show confidence intervals for all regulatory interactions of Hb and Kr. Panel titles indicate regulatory targets. X-axes indicate number of individual fitting solutions. Dependent intervals are shown in green, independent intervals in red.

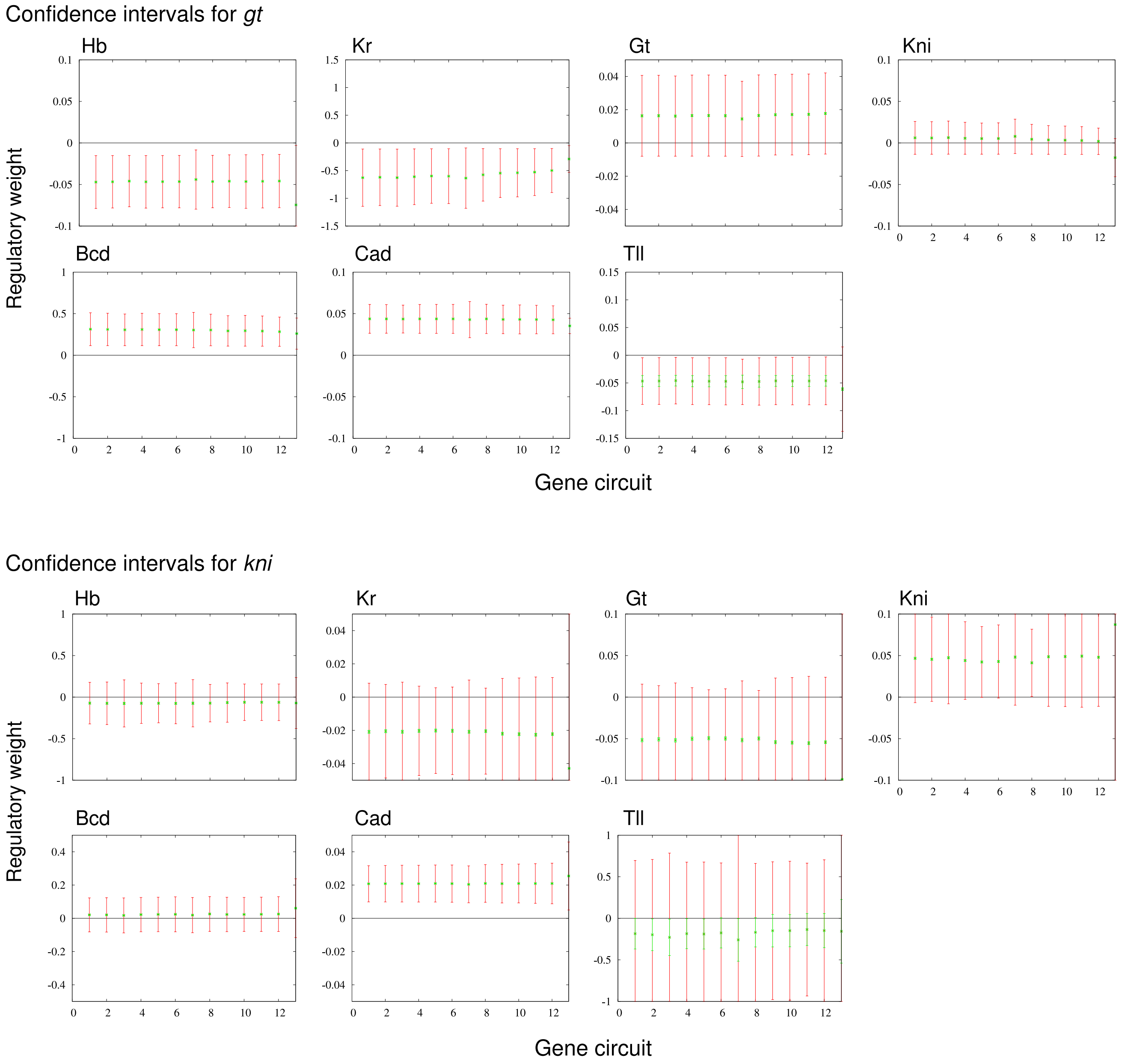

Figure S4.2. Confidence intervals for parameter determinability analysis. Fits to protein data approximated by our mRNA data processing method (data set 1). Plots show confidence intervals for all regulatory interactions of Gt and Kni. Panel titles indicate regulatory targets. X-axes indicate number of individual fitting solutions. Dependent intervals are shown in green, independent intervals in red.

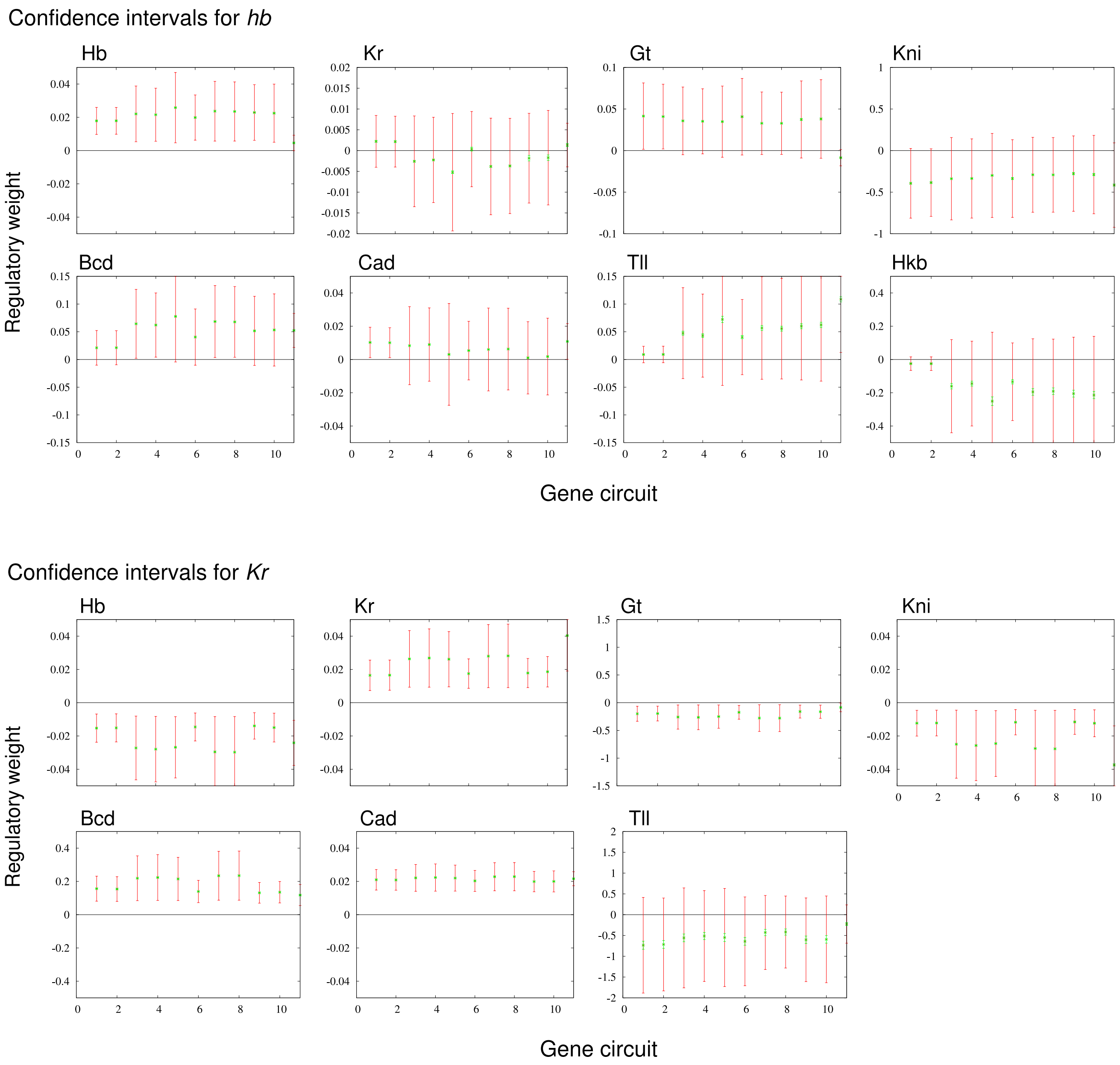

Figure S4.3. Confidence intervals for parameter determinability analysis. Fits to protein data with approximated weights for WLS (data set 2). Plots show confidence intervals for all regulatory interactions of Hb and Kr. Panel titles indicate regulatory targets. X-axes indicate number of individual fitting solutions. Dependent intervals are shown in green, independent intervals in red.

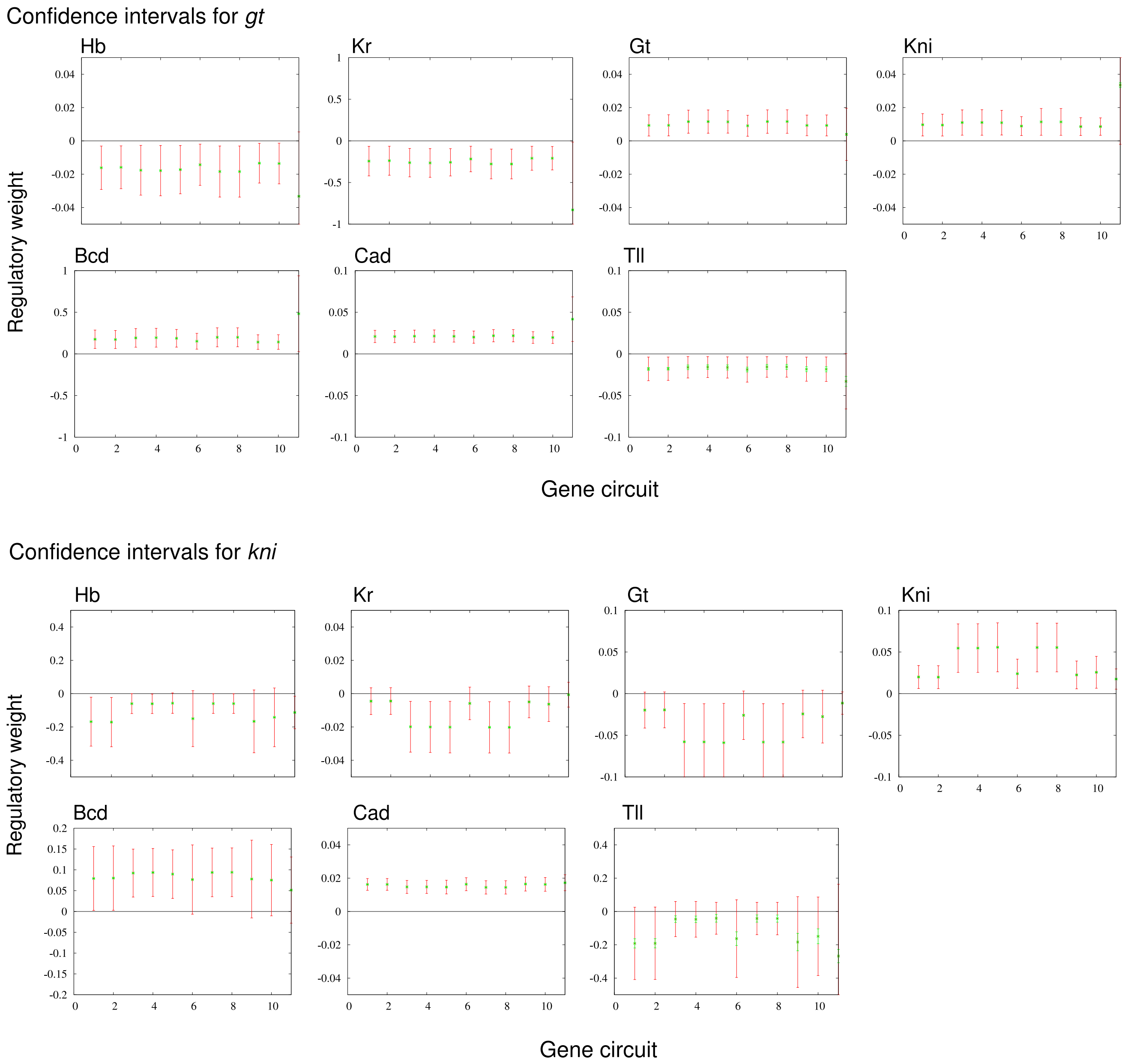

Figure S4.4. Confidence intervals for parameter determinability analysis. Fits to protein data with approximated weights for WLS (data set 2). Plots show confidence intervals for all regulatory interactions of Gt and Kni. Panel titles indicate regulatory targets. X-axes indicate number of individual fitting solutions. Dependent intervals are shown in green, independent intervals in red.

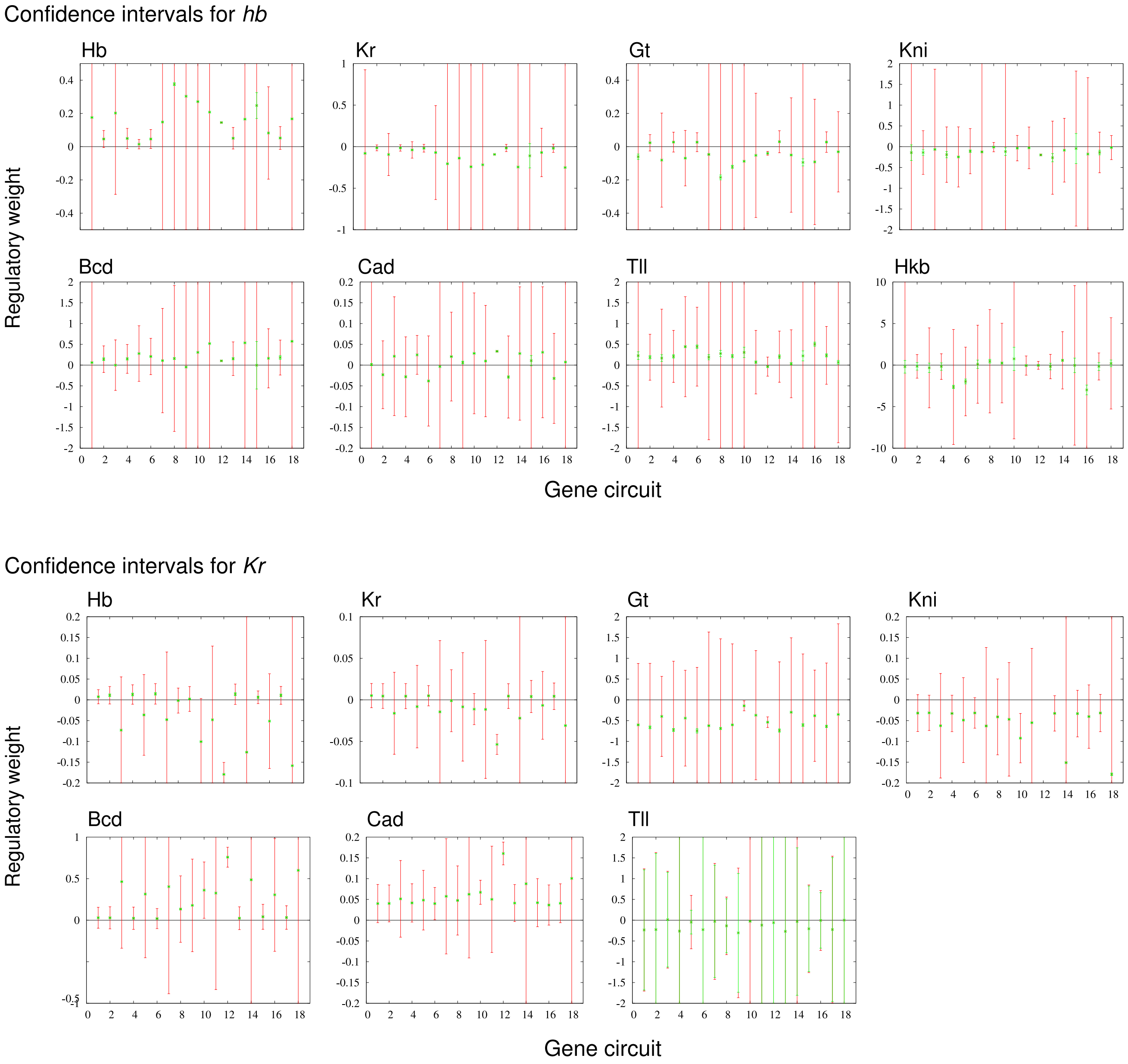

Figure S4.5. Confidence intervals for parameter determinability analysis. Fits to mRNA data using OLS cost function (data set 3). Plots show confidence intervals for all regulatory interactions of Hb and Kr. Panel titles indicate regulatory targets. X-axes indicate number of individual fitting solutions. Dependent intervals are shown in green, independent intervals in red.

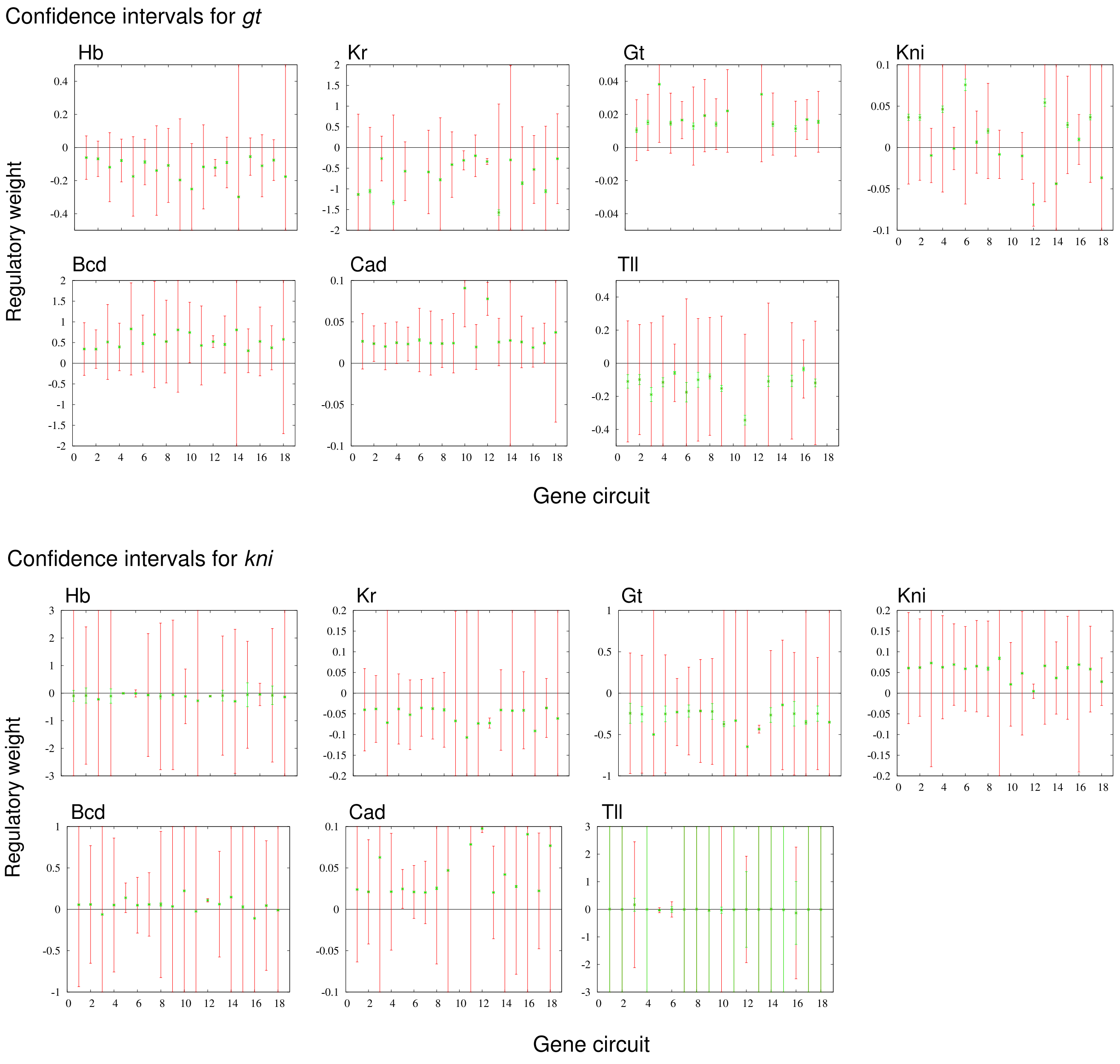

Figure S4.6. Confidence intervals for parameter determinability analysis. Fits to mRNA data using OLS cost function (data set 3). Plots show confidence intervals for all regulatory interactions of Gt and Kni. Panel titles indicate regulatory targets. X-axes indicate number of individual fitting solutions. Dependent intervals are shown in green, independent intervals in red.

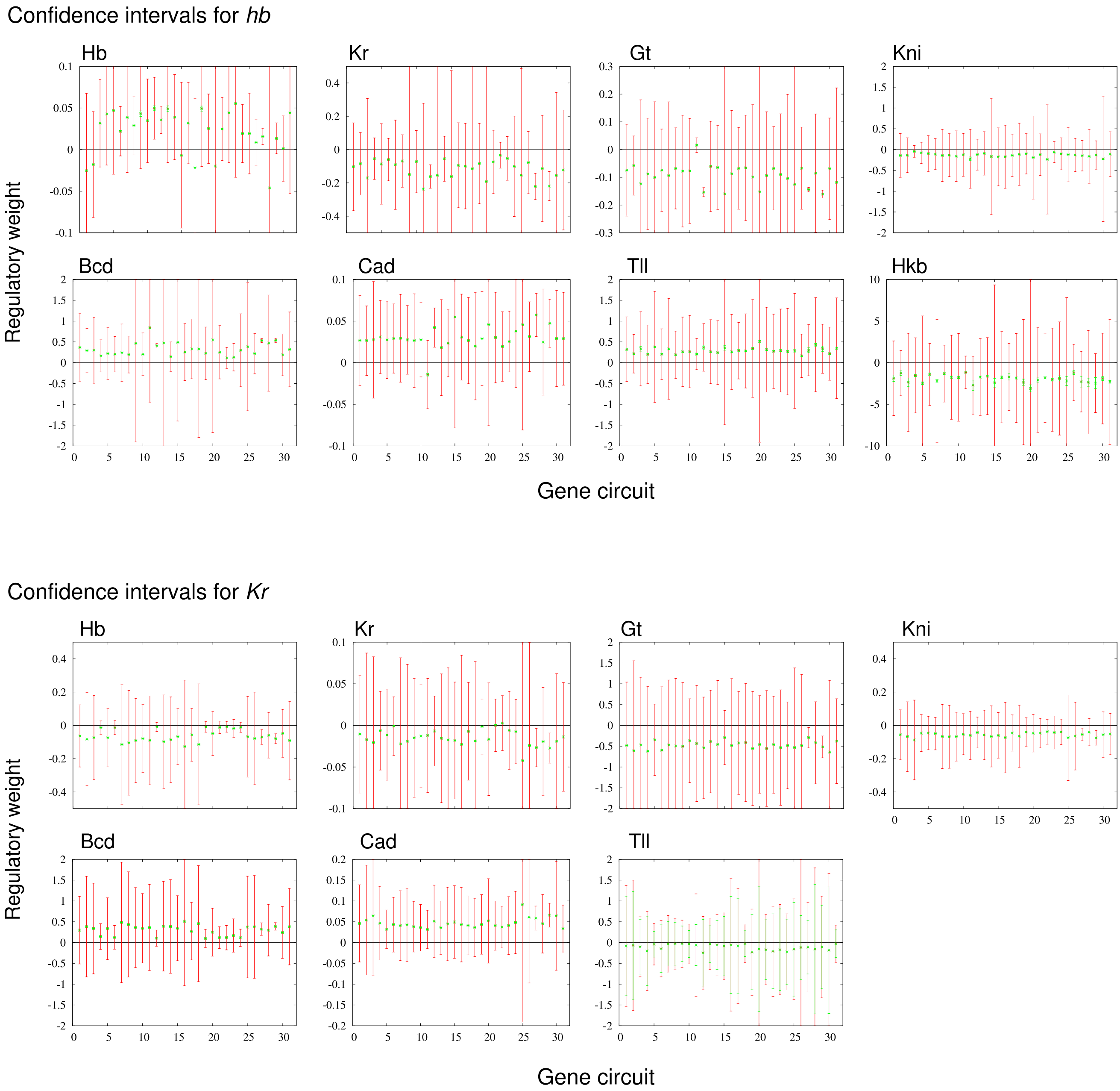

Figure S4.7. Confidence intervals for parameter determinability analysis. Fits to mRNA data using WLS cost function (data set 4). Plots show confidence intervals for all regulatory interactions of Hb and Kr. Panel titles indicate regulatory targets. X-axes indicate number of individual fitting solutions. Dependent intervals are shown in green, independent intervals in red.

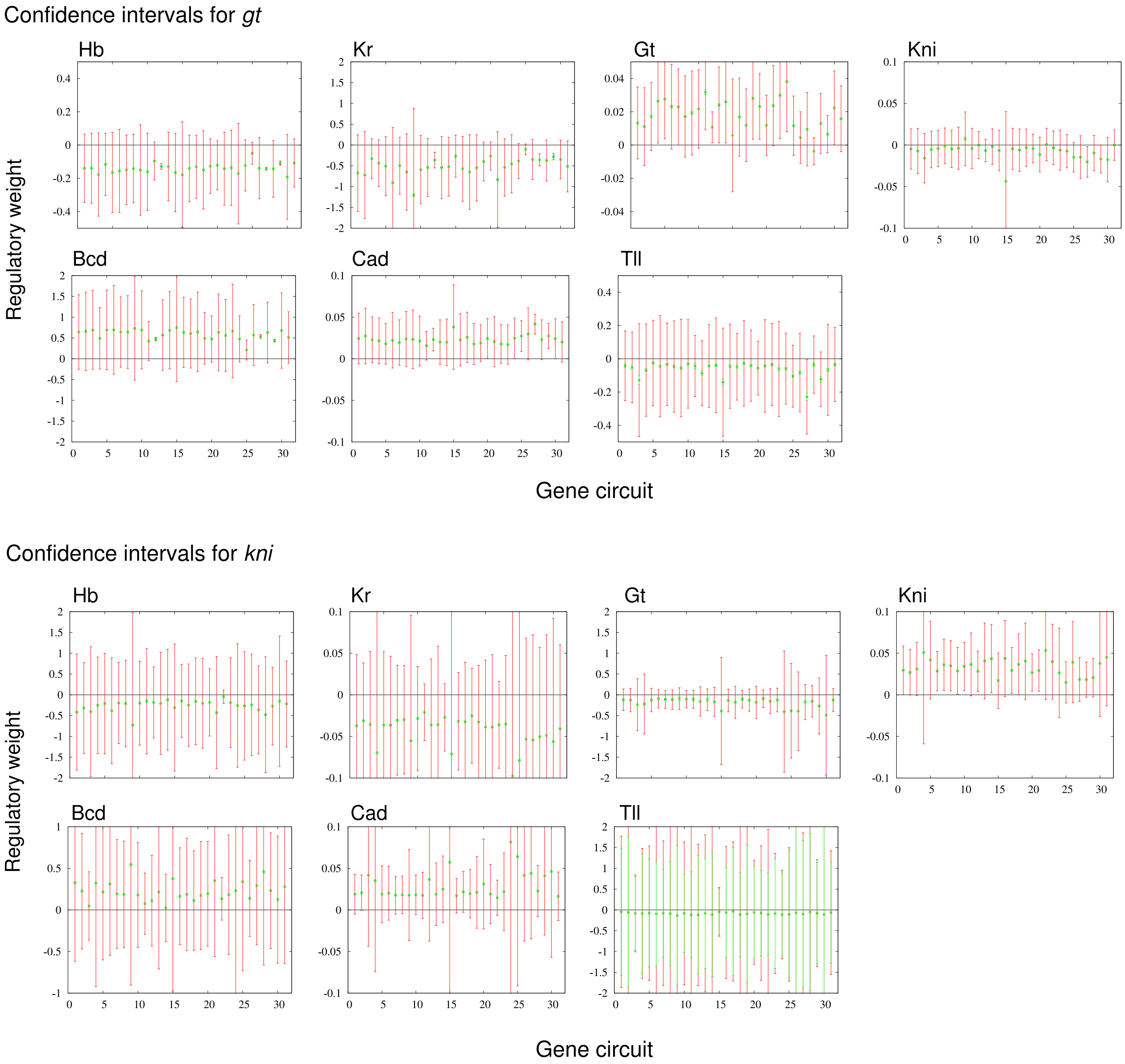

Figure S4.8. Confidence intervals for parameter determinability analysis. Fits to mRNA data using WLS cost function (data set 4). Plots show confidence intervals for all regulatory interactions of Gt and Kni. Panel titles indicate regulatory targets. X-axes indicate number of individual fitting solutions. Dependent intervals are shown in green, independent intervals in red.

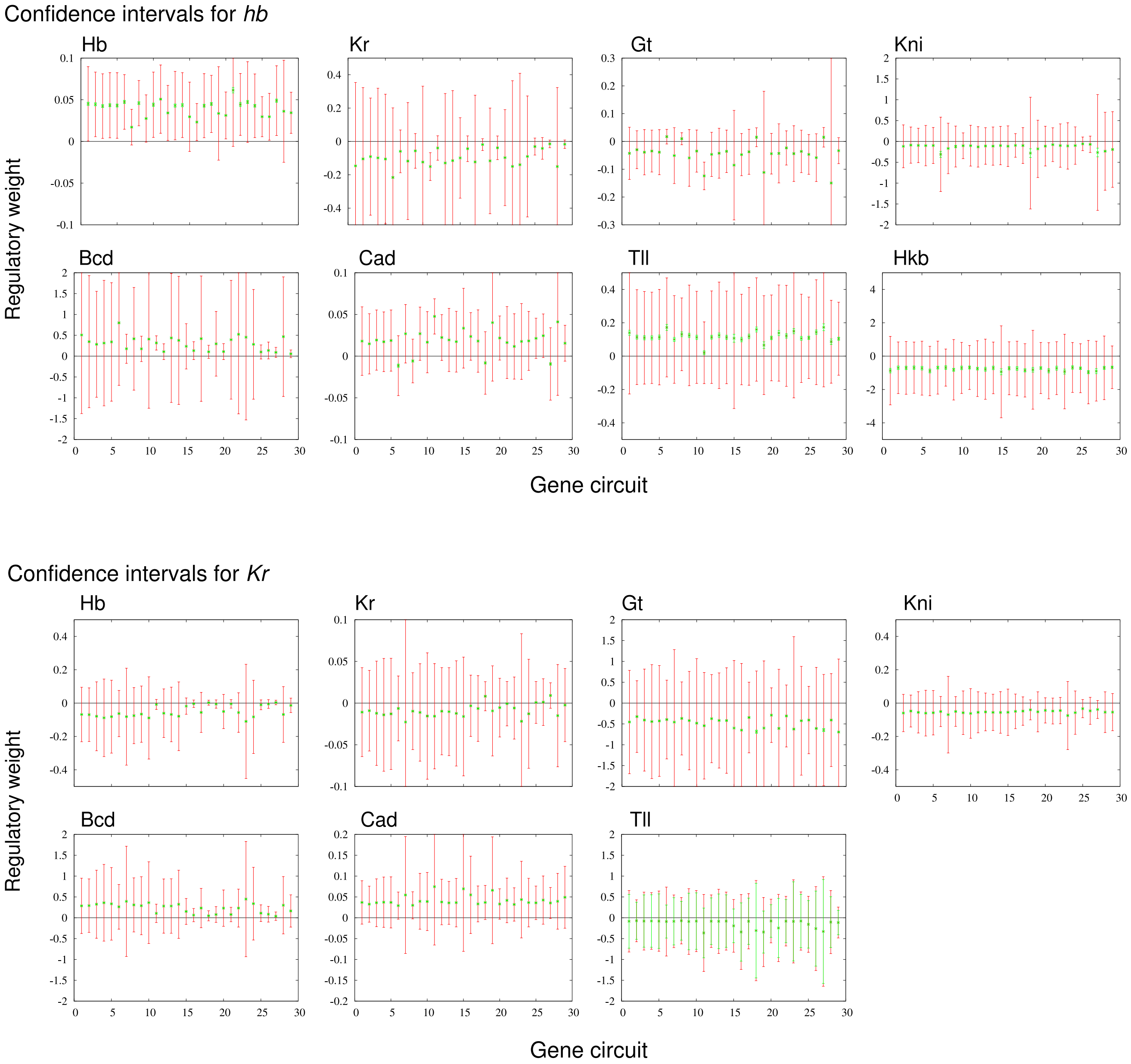

Figure S4.9. Confidence intervals for parameter determinability analysis. WLS fits to extended mRNA data covering 58 nuclei (data set 5). Plots show confidence intervals for all regulatory interactions of Hb and Kr. Panel titles indicate regulatory targets. X-axes indicate number of individual fitting solutions. Dependent intervals are shown in green, independent intervals in red.

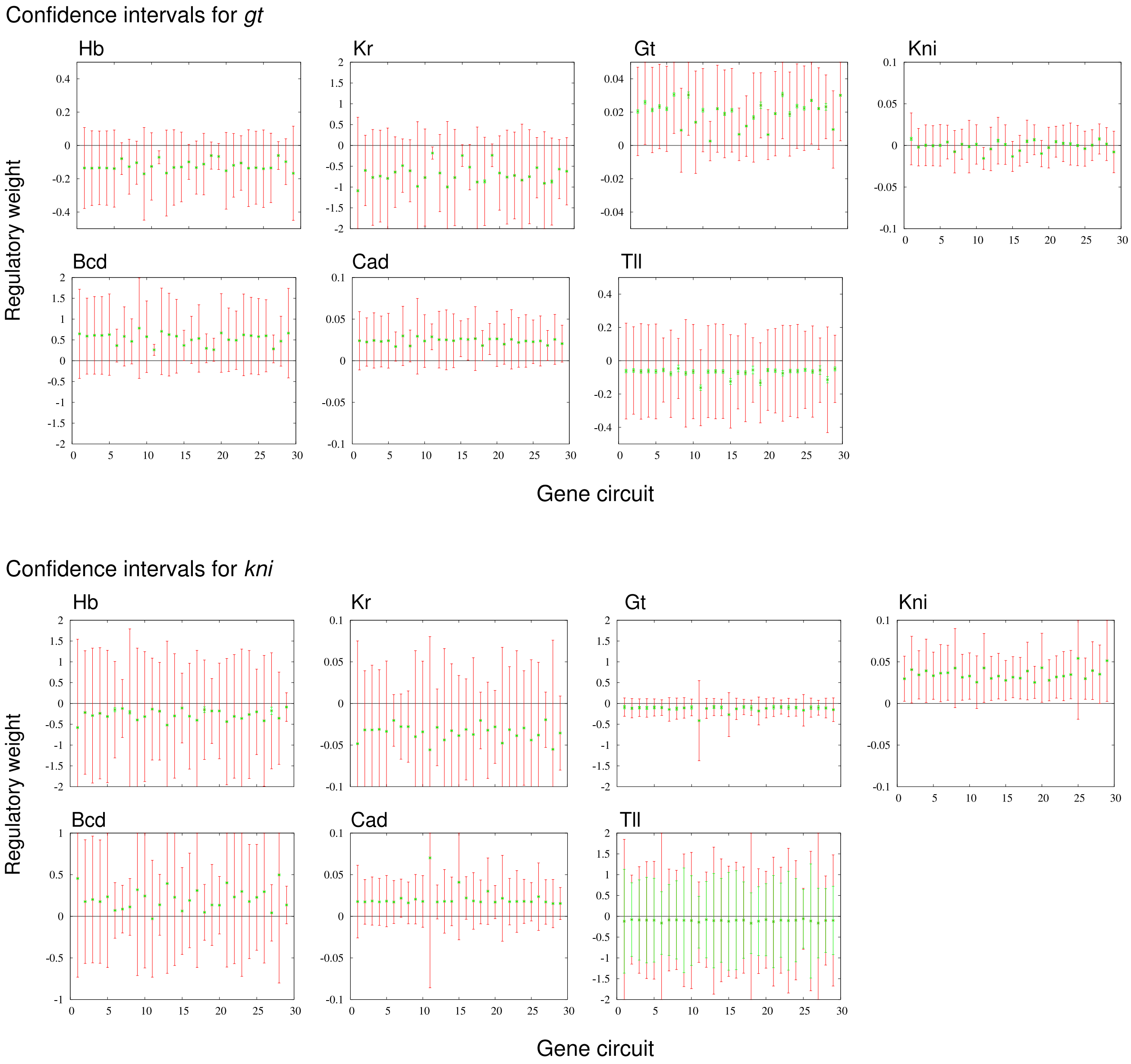

Figure S4.10. Confidence intervals for parameter determinability analysis. WLS fits to extended mRNA data covering 58 nuclei (data set 5). Plots show confidence intervals for all regulatory interactions of Gt and Kni. Panel titles indicate regulatory targets. X-axes indicate number of individual fitting solutions. Dependent intervals are shown in green, independent intervals in red.
